# A morphometric system to distinguish sheep and goat postcranial bones

**DOI:** 10.1371/journal.pone.0178543

**Published:** 2017-06-08

**Authors:** Lenny Salvagno, Umberto Albarella

**Affiliations:** Department of Archaeology, University of Sheffield, Northgate House, Sheffield, United Kingdom; New York Institute of Technology, UNITED STATES

## Abstract

Distinguishing between the bones of sheep and goat is a notorious challenge in zooarchaeology. Several methodological contributions have been published at different times and by various people to facilitate this task, largely relying on a macro-morphological approach. This is now routinely adopted by zooarchaeologists but, although it certainly has its value, has also been shown to have limitations. Morphological discriminant criteria can vary in different populations and correct identification is highly dependent upon a researcher’s experience, availability of appropriate reference collections, and many other factors that are difficult to quantify. There is therefore a need to establish a more objective system, susceptible to scrutiny. In order to fulfil such a requirement, this paper offers a comprehensive morphometric method for the identification of sheep and goat postcranial bones, using a sample of more than 150 modern skeletons as a basis, and building on previous pioneering work. The proposed method is based on measurements—some newly created, others previously published–and its use is recommended in combination with the more traditional morphological approach. Measurement ratios, used to translate morphological traits into biometrical attributes, are demonstrated to have substantial diagnostic potential, with the vast majority of specimens correctly assigned to species. The efficacy of the new method is also tested with Discriminant Analysis, which provides a successful verification of the biometrical indices, a statistical means to select the most promising measurements, and an additional line of analysis to be used in conjunction with the others.

## 1. Introduction

Despite being closely related, sheep (*Ovis aries*) and goat (*Capra hircus*) differ in many aspects, ranging from their behaviour to the products that they can provide us with. The distinction between sheep and goat bones in archaeology is important in order to clarify core aspects of human-animal relationships and the uses to which these animals were put in the past and in different parts of the world. As pointed out in a seminal paper on the subject, this is not an easy task: “It is well known that to distinguish between the bones of sheep and goat presents great difficulties” [1: 331]. In addition, some disagreement exists among zooarchaeologists regarding which are the morphological criteria that are most useful for the distinction of these two species (e.g. [2]; [3]; [4]).

Boessneck’s contribution [1], alongside other pioneering works (*i*.*e*. [5]; [6]; [7]) paved the way for the development of several other studies. Some focused on providing new diagnostic morphological traits, as well as checking their reliability on a variety of modern and archaeological samples ([1]; [8]; [9]; [4]). Others, aware of the limitations of a purely morphological approach, introduced a biometrical perspective to the problem (e.g. [10]; [11]; [12]; [13]; [14]). This paper expands substantially the approach proposed by these latter authors by using a great variety of skeletal parts and a substantial modern sample to monitor the effectiveness of different morphometric criteria. The morphometric approach not only has the advantage of providing another identification tool to be used alongside the more established morphological criteria, but, most importantly, constitutes a more objective method, as identifications can be subjected to scrutiny.

The main aims of this paper are:

- To carry out a morphometric evaluation of the distinction between post-cranial bones of sheep and goat based on a sample of modern skeletons mainly deriving from central and northern Europe (an area largely untapped by previous studies)
- To propose a set of measurements that can be used effectively to address the issue of sheep/goat distinction
- To propose a set of analytical tools that can make best use of these measurements.

## 2. Materials and methods

We visited different institutions in order to collect data from a large sample of modern sheep and goat skeletons. Most of the sheep derive from the Historic England collection held at Fort Cumberland, Portsmouth, UK. This collection has provided a large number of specimens of different age and sex–mainly unimproved Shetland and Soay breeds. These breeds were considered most suitable for archaeological purposes because of their retention of primitive traits ([15]; [16]). Sheep specimens of other breeds have, however, also been recorded. These include a few Mediterranean specimens kept at the Zooarchaeology Laboratory of the University of Sheffield and some German, Alpine and Near Eastern breeds from the Natural History Museum of Berlin and the Zooarchaeology Laboratory of the University of Kiel (Germany).

Most of the modern goat skeletons derive from the Museum für Haustierkunde ‘Julius Kühn’ in Halle (Germany), the Zoarchaeology Laboratory of the University of Kiel (Germany) and the Natural History Museum in Berlin (Germany), with additional specimens from the Zooarchaeology Laboratory of the University of Sheffield, the Zooarchaeology Laboratory of the University of York and the Barbara Noddle collection of English goats kept at the National Museum in Cardiff. Table 1 summarises the number of specimens that comprise the modern sample. For a more detailed list of the modern specimens see Tables A-B in S1 Table. Taxonomic, age, and sex details of these specimens are those provided in the collections’ databases. This information should be reliable, as in many cases the life history of the animals was known, and there were no cases, during recording, in which the attribution of specimens to one or the other species, looked suspect. All specimens are available and accessible in the above mentioned permanent repositories.

**Table 1:** Total number of sheep and goat specimens included in the study. ‘Almost complete’ specimens only have one or two missing bones, while ‘incomplete’ specimens had more than two missing bones.

Species	Total Number	Complete	Almost complete	Incomplete
*Ovis aries*	78	37	41	0
*Capra hircus*	79	28	47	4
	Female	Male	Wether	Sex unknown
*Ovis aries*	29	14	17	18
*Capra hircus*	31	23	-	25

The sample is not entirely even in terms of sex and age distribution. For both taxa, particularly sheep, there is a predominance of females, while castrates are only represented in the sheep group (Table 1).

Although the age at death was known for a few specimens, this was also assessed using the Payne’s criteria ([17]; [18]), to evaluate the whole sample with the same system. The youngest stages (mandibular wear stages A to C *sensu* Payne [17]) are under-represented in both sheep and goat, and there are more old goats than old sheep (H and I *sensu* Payne [17]).

A study of the reliability and visibility of the morphological traits according to the age and sex of the animals was undertaken by Salvagno ([19]) and the results have confirmed what was previously observed ([15]; [4]; [20]), namely that sex has little influence on the visibility and reliability of the traits, whereas some morphological characteristics may vary with age. This will have to be considered in the interpretation, though we need to accept that it is virtually impossible to find samples of skeletons of the two species which have exactly the same sex and age distributions.

- A selection of morphological criteria that could relatively easily be translated into measurements was based on observations made by previous studies. Whenever possible, measurements proposed and defined by von den Driesch ([21]) were taken, to enhance comparability with other studies. To these we added measurements specifically designed for the sheep/goat distinction recommended by others ([10]; [11]; [13]), as well as ourselves. S2 Table provides the list of selected bones and measurements. The anatomical elements were selected according to two main criteria:
- their degree of survivability in the archaeological context (for example the femur was disregarded as it tends to survive poorly)
- their morphological distinctiveness (based on the authors’ experience and previous literature)

Illustrations on how to take the newly defined measurements can be found in Figs 1–13. All measurements were taken using digital callipers and were approximated to the tenth of millimetre.

**Fig 1:**
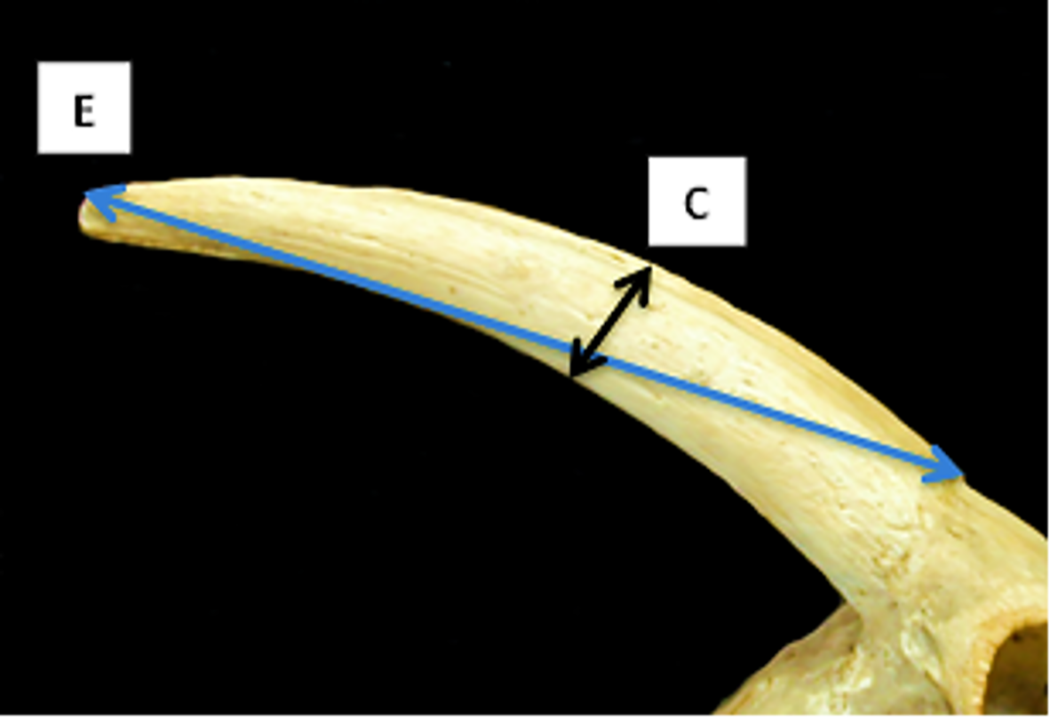
Horncore: Newly introduced measurements C (maximum diameter taken midway the horncore length) and E (length of the horncore from the antero-medial edge of the base to the tip).

**Fig 2:**
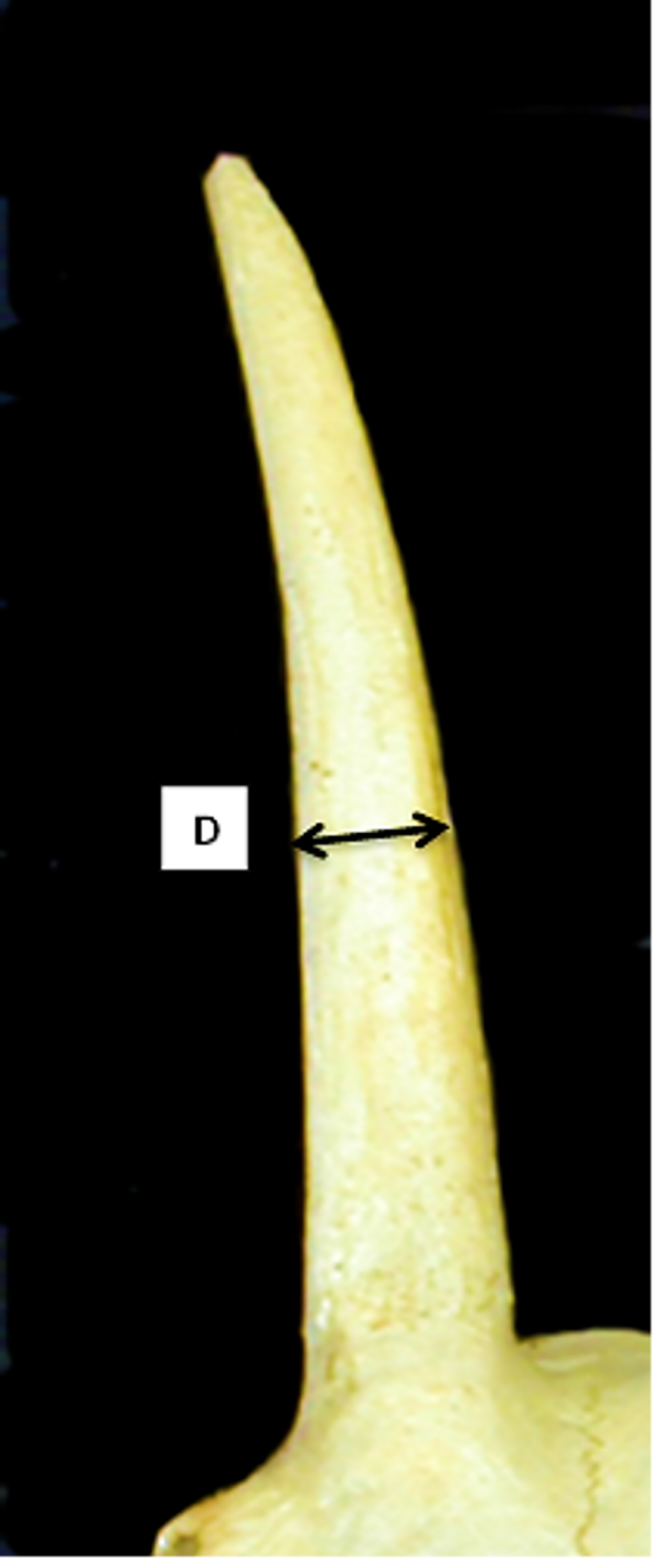
Horncore: Newly introduced measurement D (minimum diameter taken midway the horncore length).

**Fig 3:**
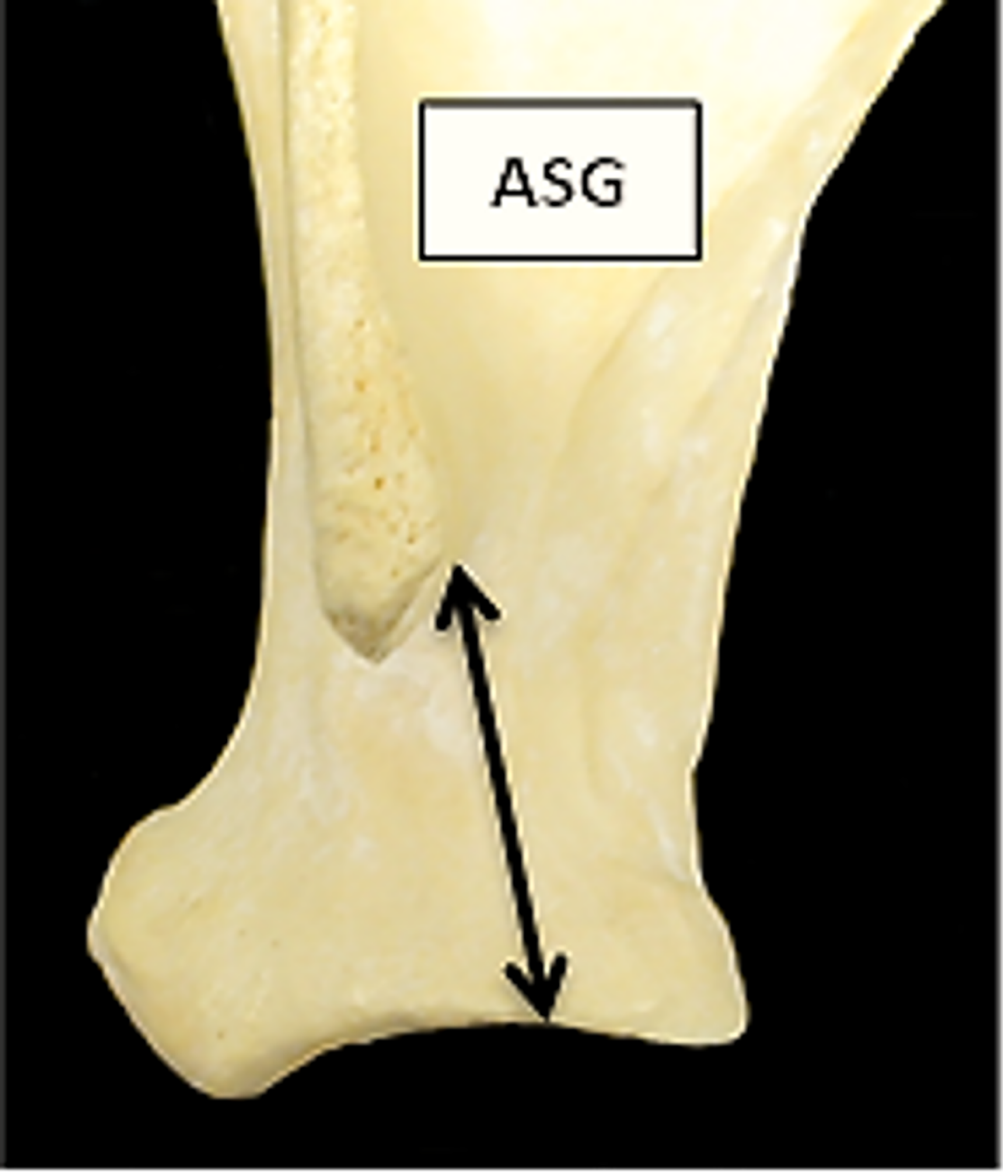
Scapula: ASG (shortest distance from the base of the spine to the edge of the glenoid cavity). This measurement has been slightly modified from previous literature.

**Fig 4:**
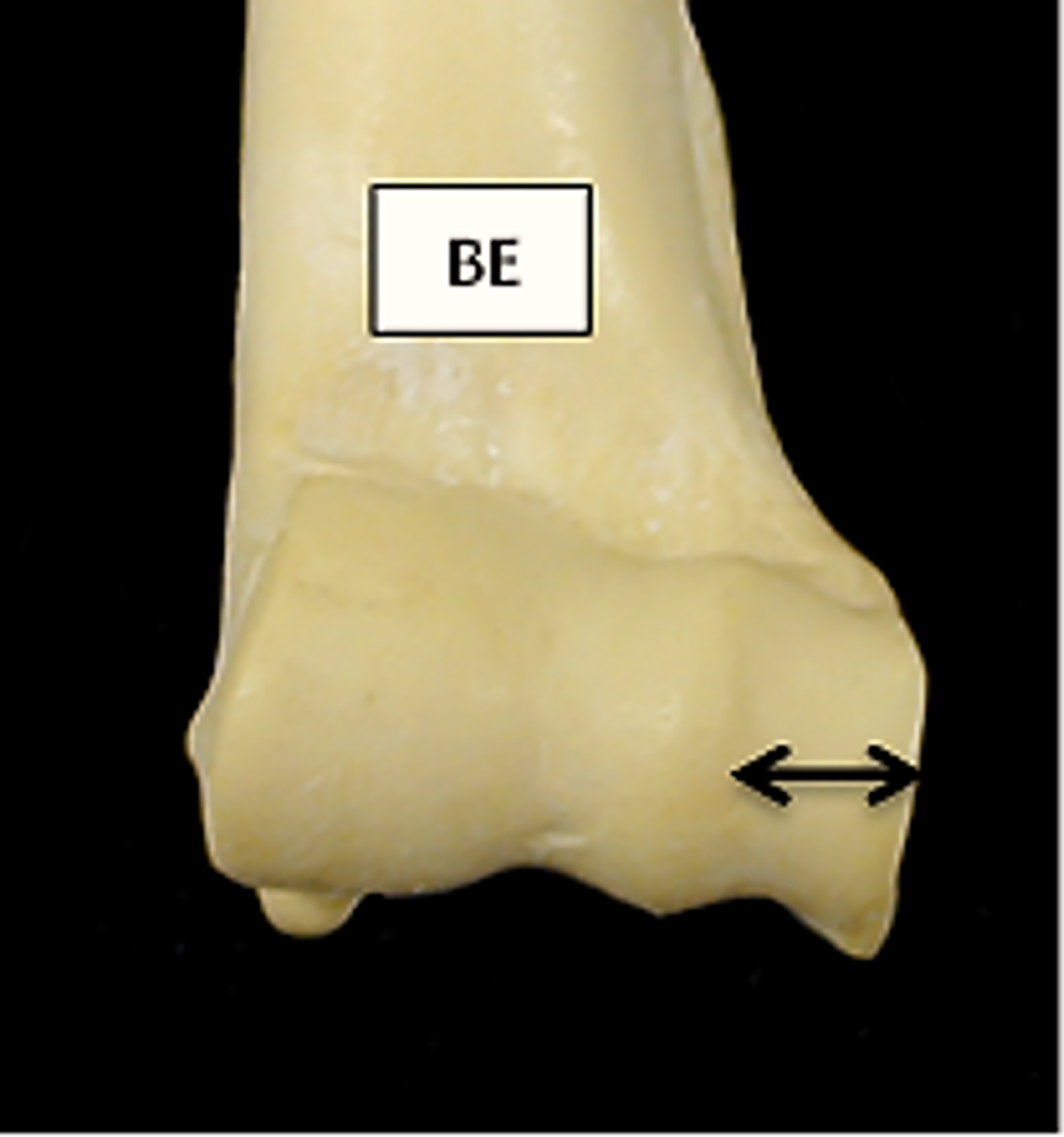
Humerus: Newly introduced measurement BE (breadth of the *capitulum*, measured along the trochlear axis).

**Fig 5:**
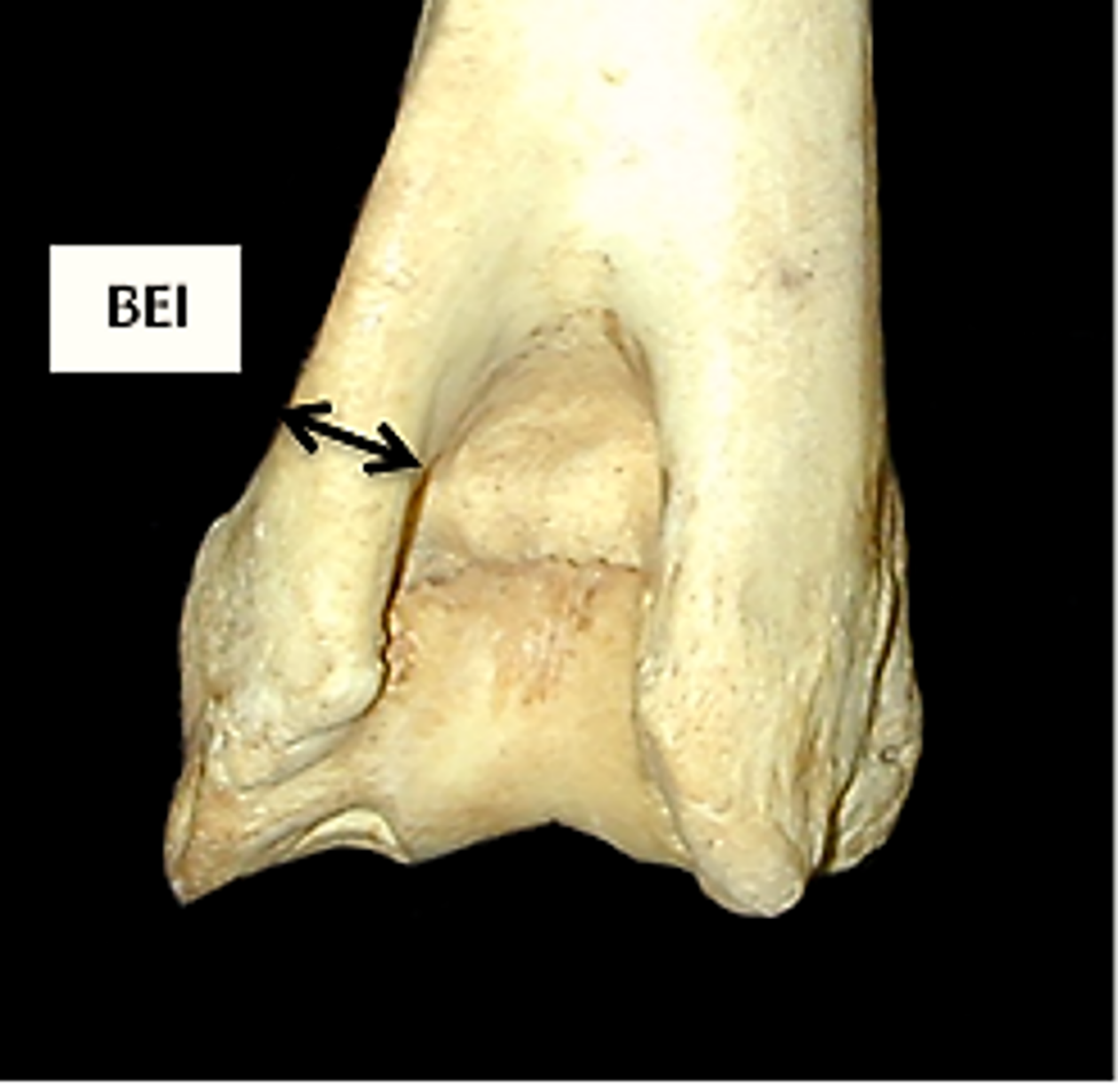
Humerus: Newly introduced measurement BEI (breadth of the epicondyle *lateralis* taken at a depth of 2–3 mm from the lateral margin).

**Fig 6:**
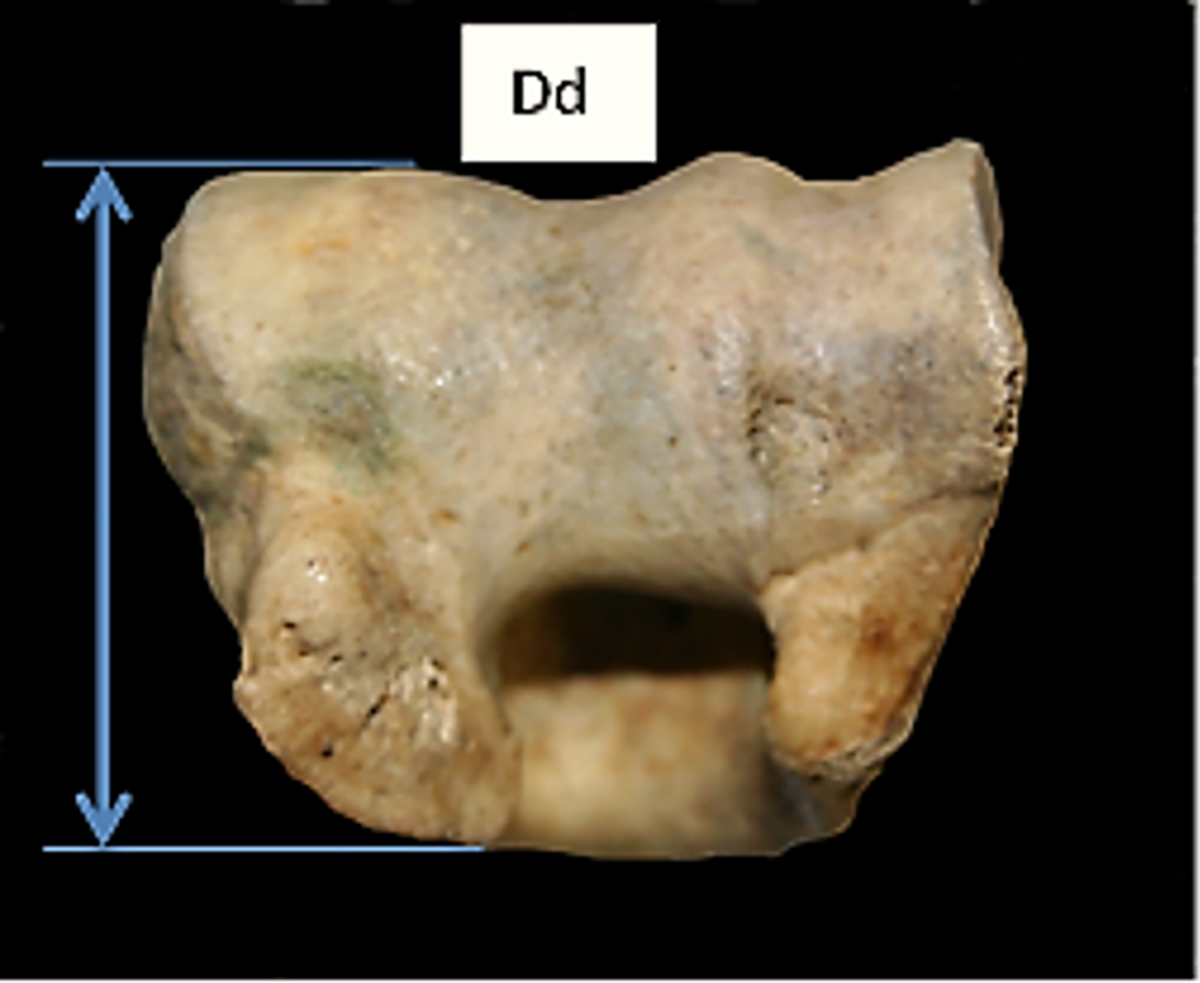
Humerus: Dd (depth of the distal end). This measurement has been slightly modified from previous literature.

**Fig 7:**
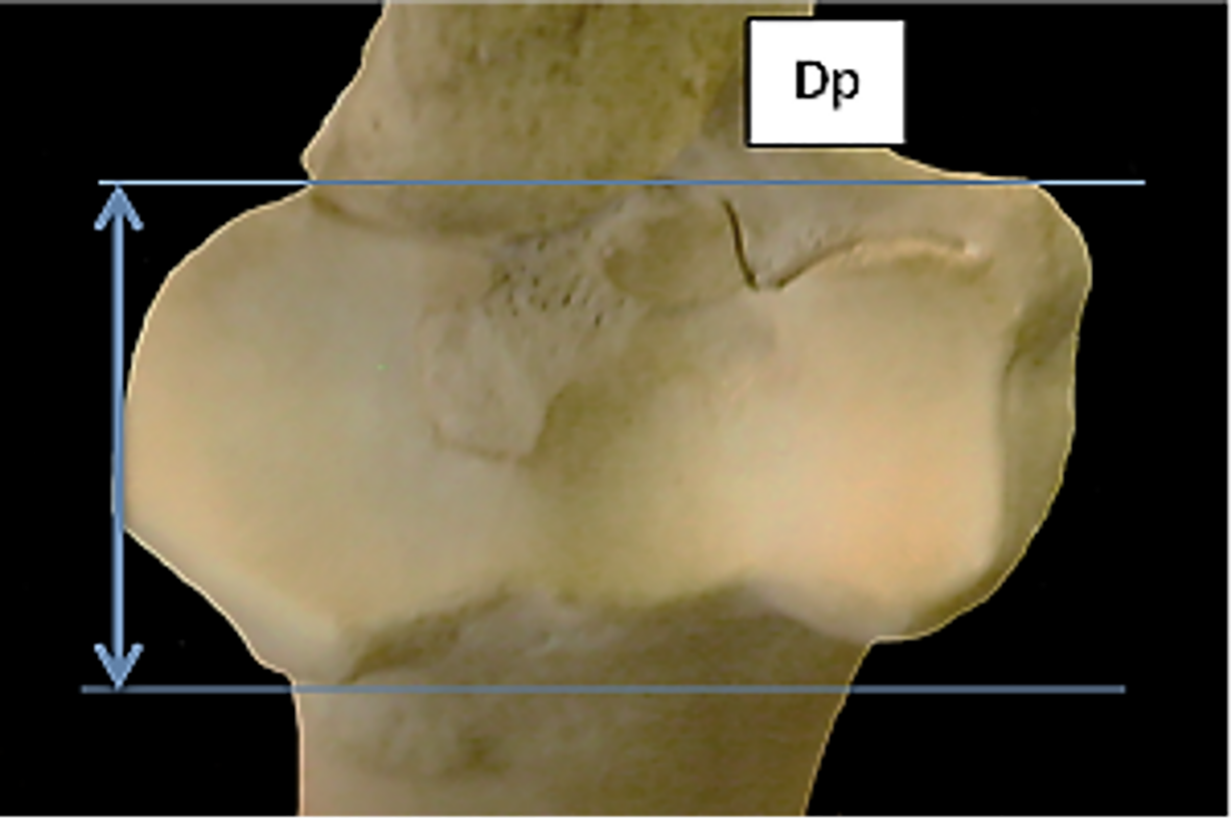
Radius: Dp (depth of the proximal end). This measurement has been slightly modified from previous literature.

**Fig 8:**
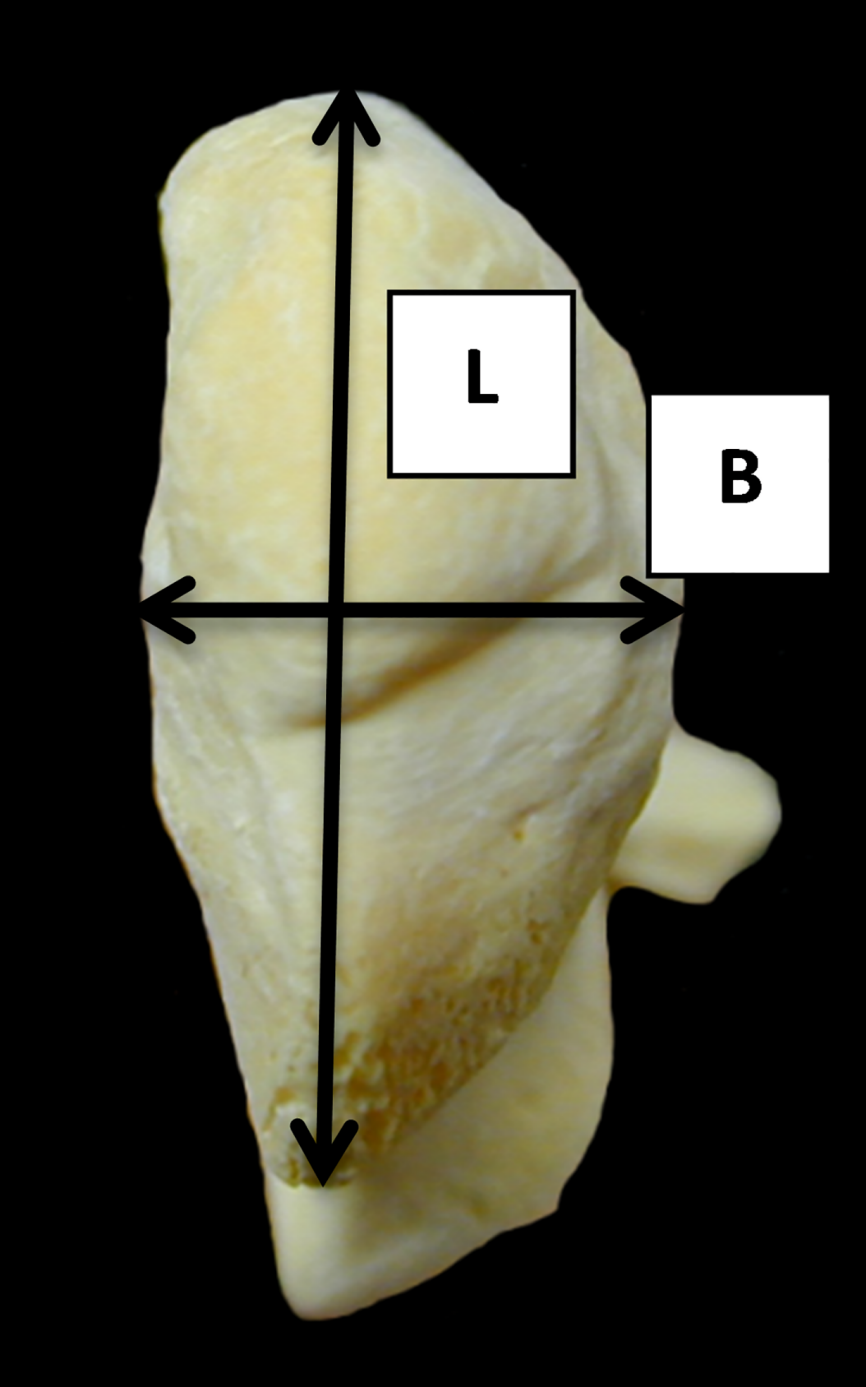
Ulna: B (breadth of the *olecranon* taken by keeping the arms of the callipers parallel to the medial face) and L (length of the *olecranon*). Both measurements have been slightly modified from previous literature.

**Fig 9:**
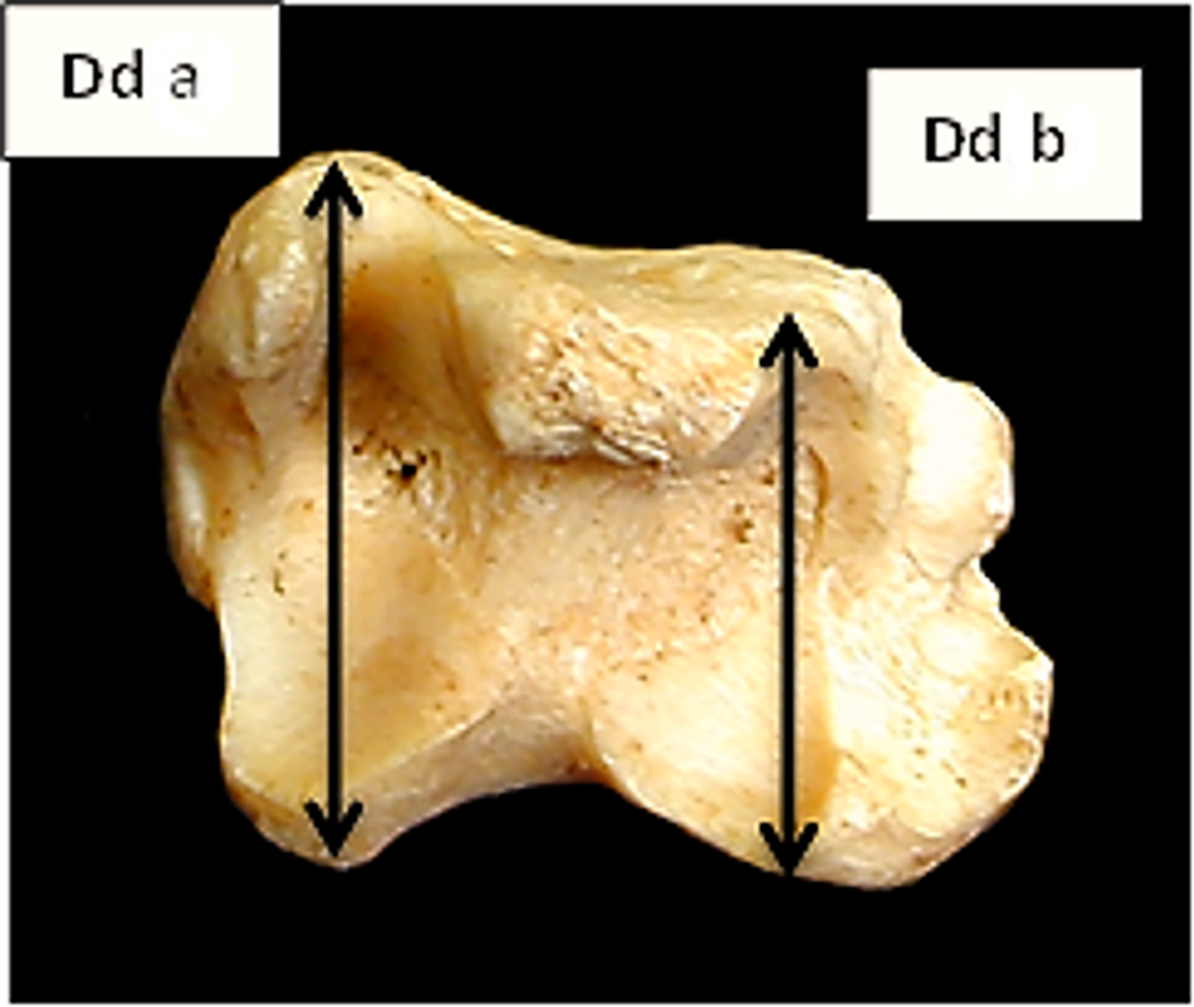
Tibia: Dda (depth of the distal end of the medial side). This measurement has been slightly modified from previous literature. Ddb (depth of the distal end of the lateral side) a newly introduced measurement.

**Fig 10:**
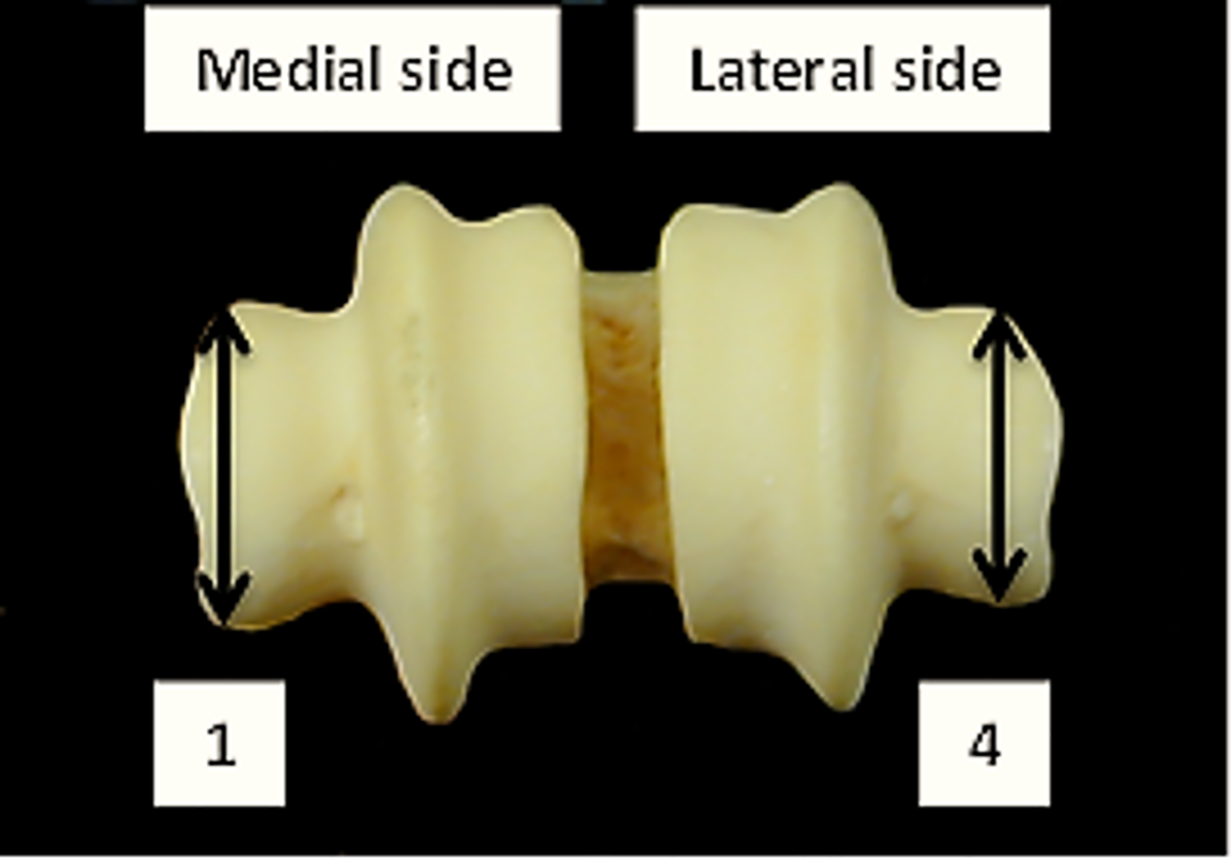
Metapodials: 1 (diameter of the external trochlea of the medial condyle. Callipers need to be positioned at the external edge of the trochlea); 4 (diameter of the external trochlea of the lateral condyle. Callipers need to be positioned at the external edge of the trochlea). Both measurements have been slightly modified from previous literature.

**Fig 11:**
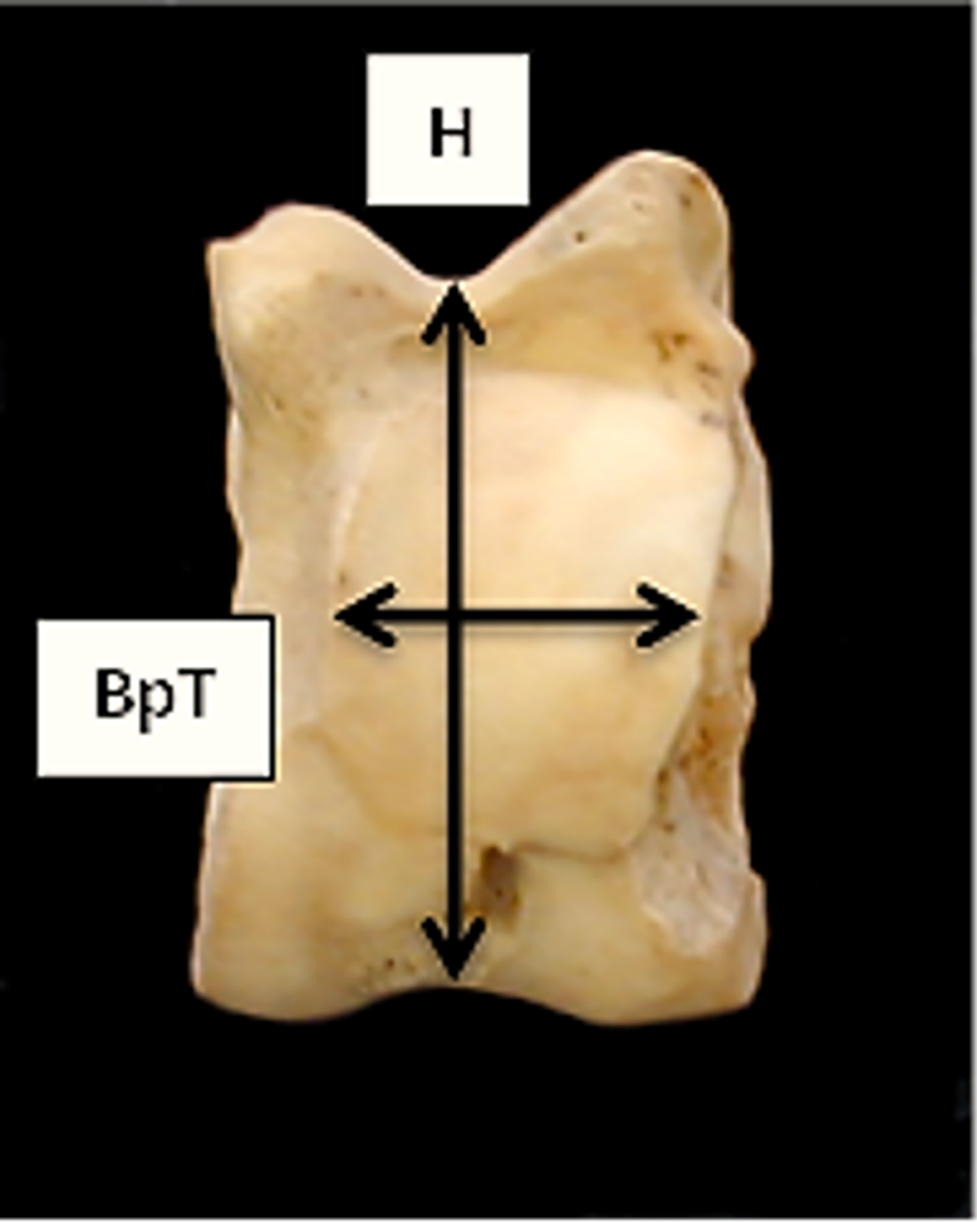
Astragalus: Newly introduced measurements H (height of the central constriction, i.e. minimum length) and BpT (smallest breadth of the plantar trochlea).

**Fig 12:**
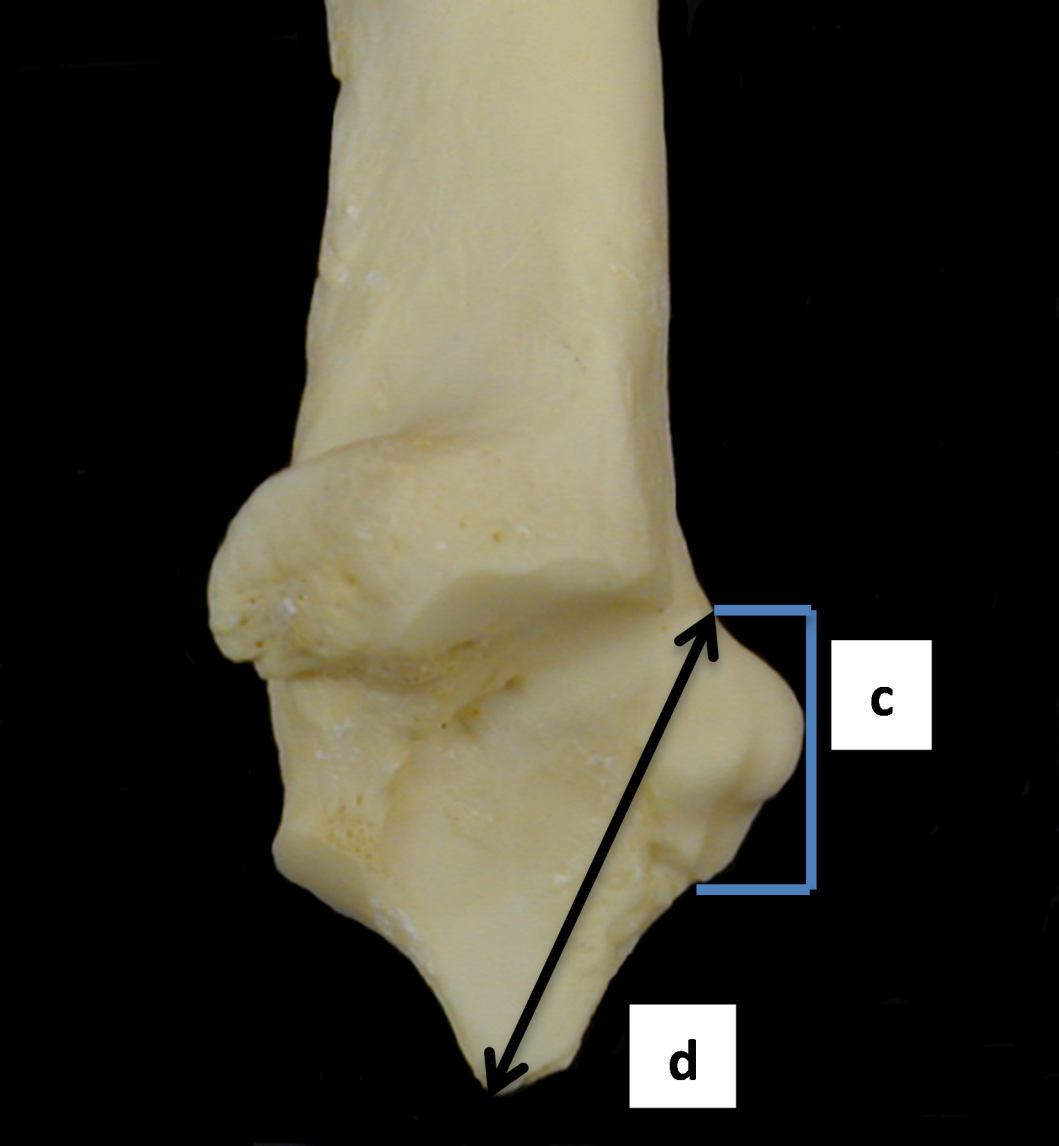
Calcaneum: c (length of the articular facet on the calcaneum taken where the articular facet starts to project out. This measurement can be tricky to take as in some specimens the articular facet coincides with the area that projects out, forming the *os malleolare*, in others the beginning of the articular facet is visible before it starts projecting out. For sake of consistency we decided to take it where the articular facet starts projecting out) and d (length from the articular facet to the articulation-free part of the process). Both measurements have been slightly modified from previous literature.

**Fig 13:**
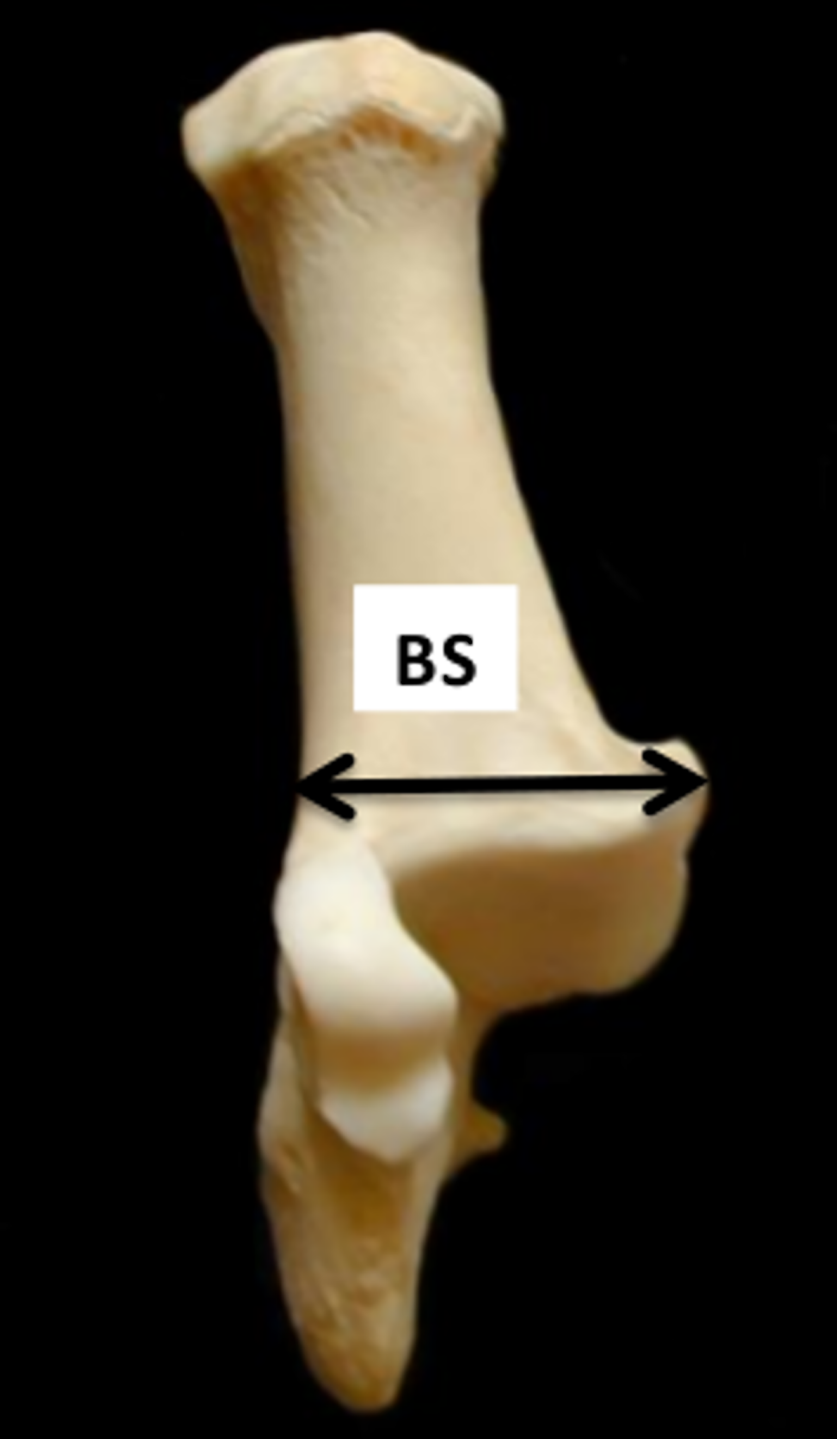
Calcaneum: BS (breadth taken at the height of the *substentaculum tali)*. This measurement has been slightly modified from previous literature.

Our original recording protocol included measurements of teeth as well as postcranial bones. However, many measurements could not be taken on teeth embedded in jaws, and this resulted in a too small sample of dental measurements. For this reason this study focuses upon postcranial bones.

All collected data were statistically tested. These analyses were performed with the use of SPSS Statistics, including: inter and intra observer error tests, Mann Whitney test of significance (along with a Bonferroni adjustment), Manova test and Discriminant Analysis (DA). DA was run for each element individually, using species as grouping variables and the chosen measurements as independent variables.

Since the new protocol included several new and revised measurements, and given that we all tend to take measurements in a slightly different way, inter- and intra-observer error studies were conducted. For the inter-observer error, the new recording protocol was presented to a group of eight colleagues, including the writers, all experienced zooarchaeologists. The trial required the measuring of two sheep and two goat skeletons. To test reliability, an Interclass Correlation Coefficient (ICC) was adopted, as this is commonly used to establish and quantify reproducibility ([22]: 187–199) as well as estimate inter-observer reliability on quantitative data ([23]: 96). The results revealed that, in general, the proposed measurements were taken consistently. The less consistent measurements were mainly those previously described in the literature, rather than the new ones. Measurements of radius, ulna, tibia and 3^rd^ phalanx (aka terminal phalanx) provided the most consistent results ([19]). For the intra-observer error, the same four specimens were repeatedly measured over several days by one of us (LS). The results revealed similar trends to those detected through the inter-observer error test. Notably all measurements, even though to a different degree, gave higher ICC values compared to the values given by the Inter-Observer Error, confirming what was observed by previous researchers ([24]; [25]; [26]; [27]), namely that the intra-observer error is generally lower than the inter-observer error (see Table A in S1 File for more details). In conclusion, both tests indicate a high degree of repeatability of the measurements included in the recording protocol.

## 3. Results

### 3.1 Biometrical indices

Biometrical indices were plotted to emphasise potential *shape* differences. Although in some populations one of the two species may have larger body *size* than the other, their variability is such that neither can universally be characterised as being larger (or smaller), therefore absolute size is of limited interest for this analysis. In a few cases, however, a linear measurement (as opposed to a ratio) was retained on one of the two axes, which means that, along that axis, the distribution will reflect size. Although, due to allometric development, shape is not entirely independent from size, the emphasis in the analysis of metric ratios is on shape. Should shape differences, to some extent, be the product of variability in size, this would not invalidate the comparison, particularly when a mix of different populations/breeds is considered.

**Fig 14:**
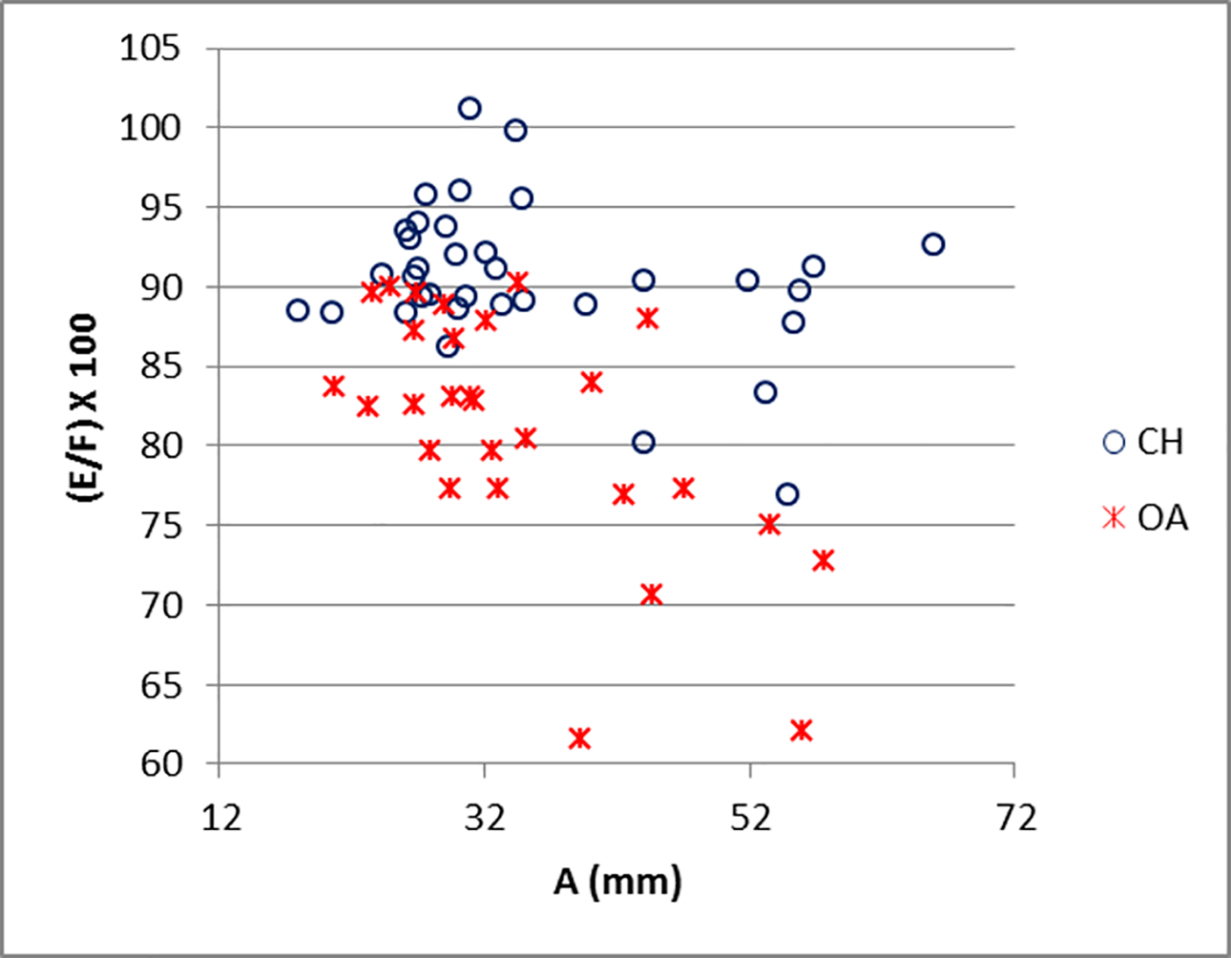
HORNCORE: Maximum diameter taken at the base (A) plotted against a ratio between length (E) and length of the outer curvature (F) of the horncore. CH = *Capra hircus*; OA = *Ovis aries*.

**Fig 15:**
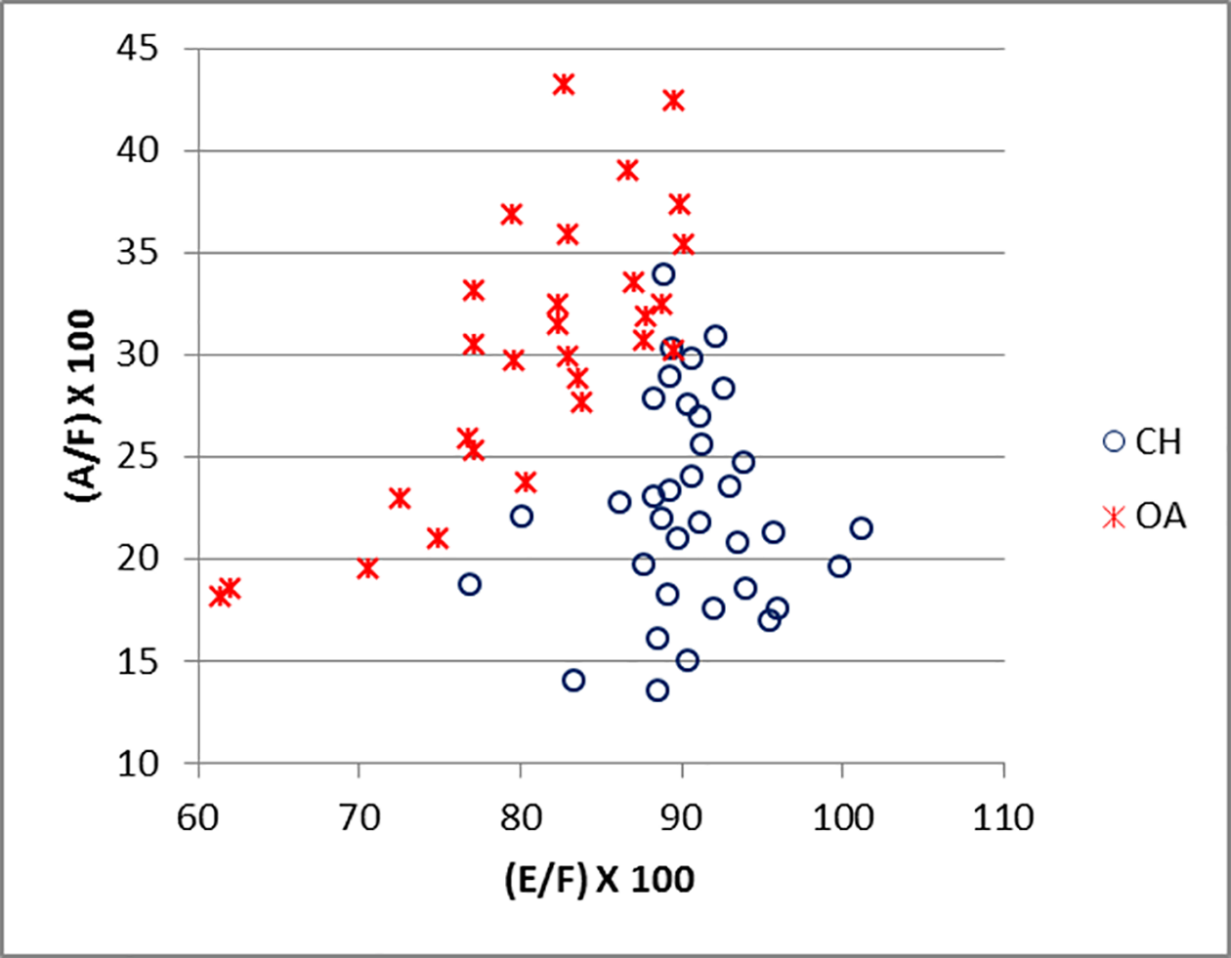
HORNCORE: Ratio between length (E) and length of the outer curvature (F) plotted against the ratio between maximum diameter taken at the base (A) and length of the outer curvature of the horncore (F). CH = *Capra hircus*; OA = *Ovis aries*.

Fig 14 shows how, by plotting the ratios E/F against measurement A, the separation between the horncores of the two species is clearly visible with a minimum amount of overlap. The ratio between the length of the horncore (E) and the length of its outer curvature (F) is the most useful characteristic to discriminate between the two groups. Sheep and goat in Fig 1A have similar maximum diameters (A) but very different E/F ratios. By plotting the ratio E/F against the ratio A/F (Fig 15), the separation between the two species is almost complete. Sheep, whose horncores are more curved and shorter, have a higher A/F value compared to goats. On the other hand, goats, which have longer, sharper and less curved horncores, have a higher E/F value. Horncores are generally easily distinguishable through simple visual observations, but this method allows us to demonstrate identifications and provide an indication of the degree of variability.

**Fig 16:**
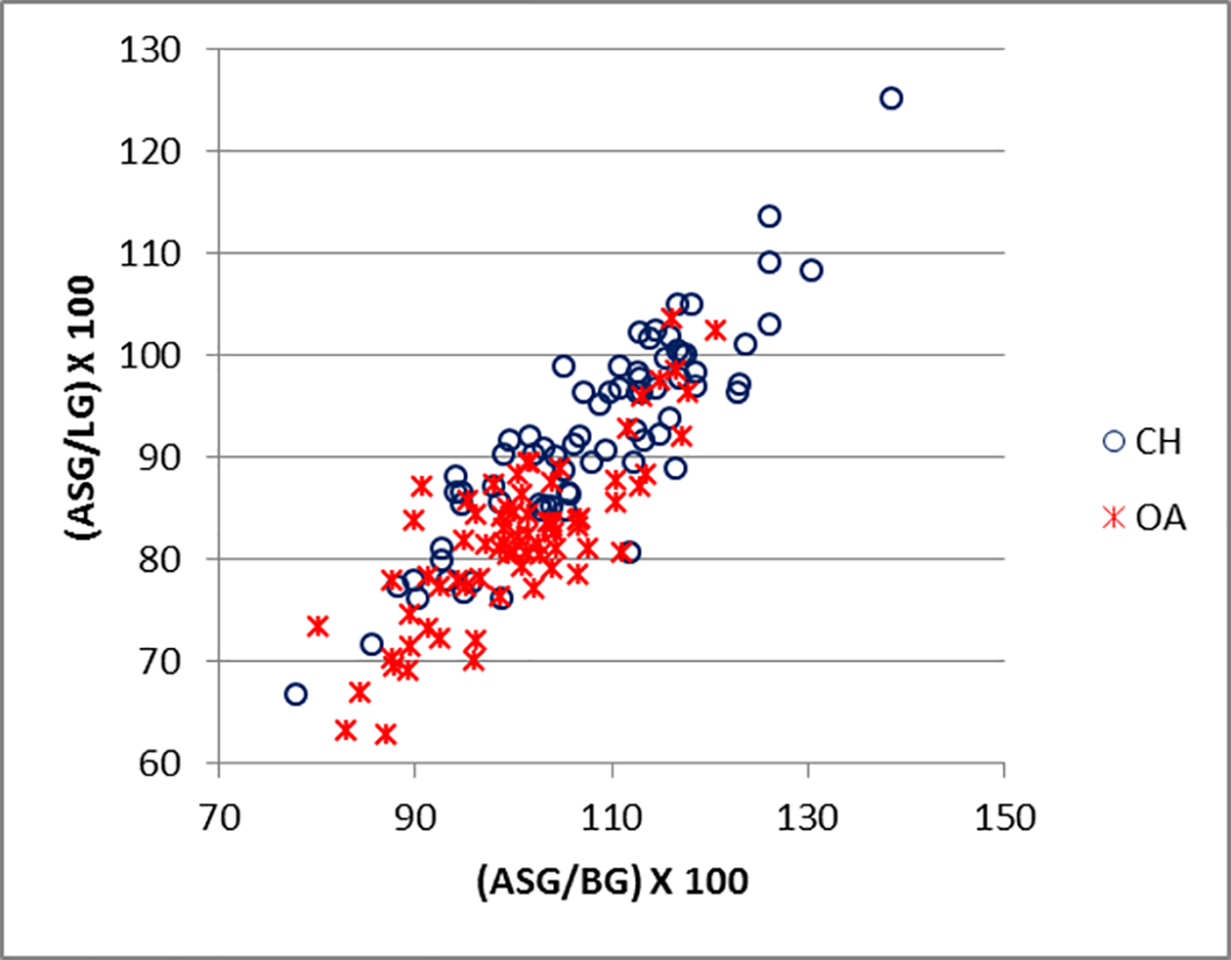
SCAPULA: Ratio between shortest distance from the base of spine to the edge of the glenoid cavity (ASG) and breadth of the glenoid cavity (BG) plotted against the ratio between the shortest distance from the base of the spine to the edge of the glenoid cavity (ASG) and length of the glenoid cavity (LG). CH = *Capra hircus*; OA = *Ovis aries*.

**Fig 17:**
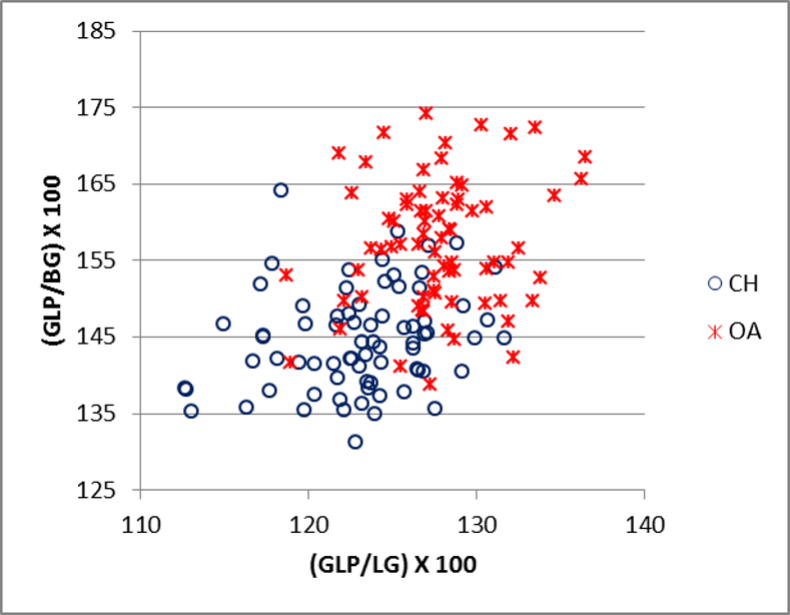
SCAPULA: Ratio between greatest length of the *processus articularis* (GLP) and length of the glenoid cavity (LG) plotted against the ratio between greatest length of the *processus articularis* (GLP) and breadth of the glenoid cavity (BG). CH = *Capra hircus*; OA = *Ovis aries*.

**Fig 18:**
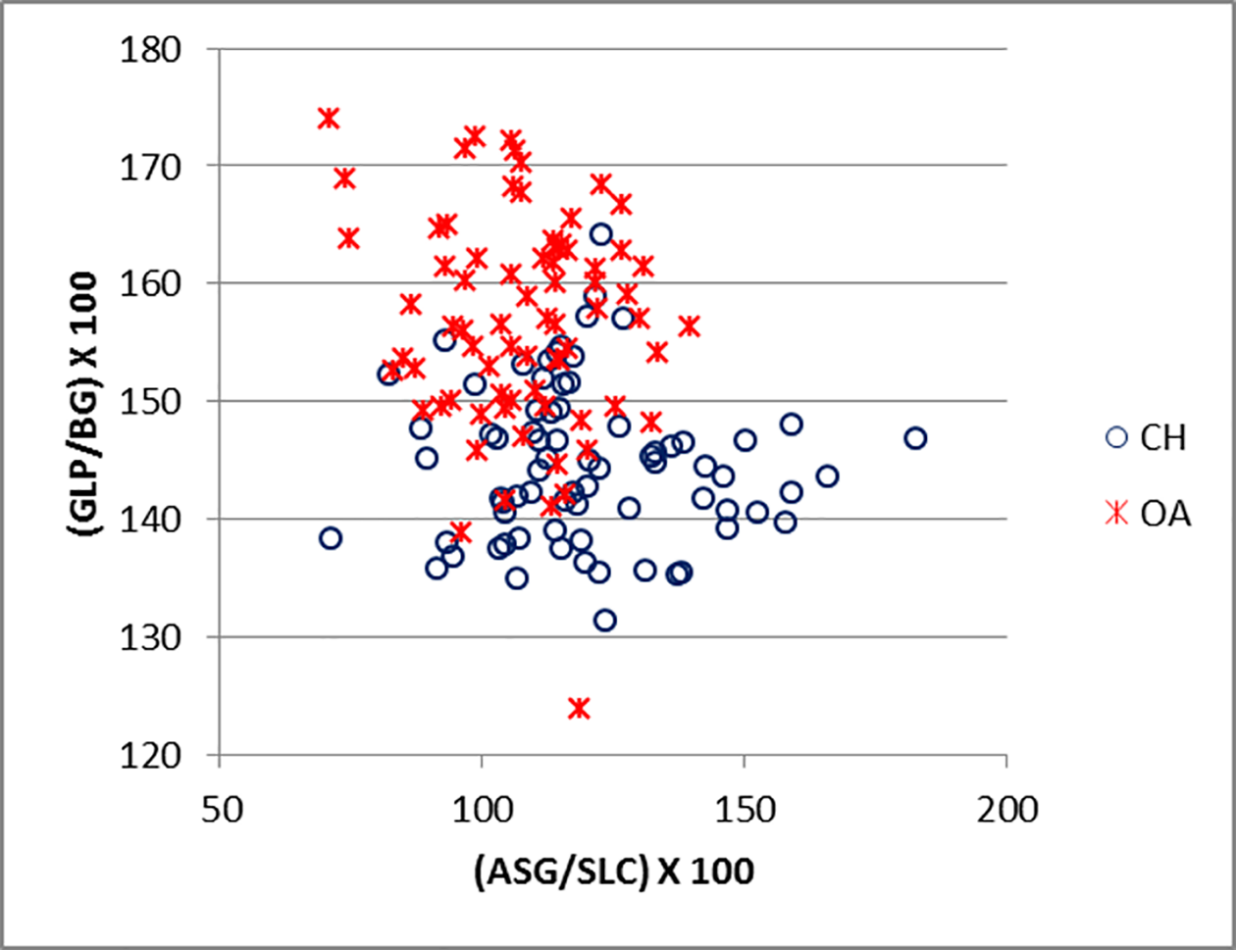
SCAPULA: Ratio between shortest distance from the base of the spine to the edge of the glenoid cavity (ASG) and smallest length of the *collum scapulae* (SLC) plotted against the ratio between greatest length of the *processus articularis* (GLP) and breadth of the glenoid cavity (BG). CH = *Capra hircus*; OA = *Ovis aries*.

Fig 16 plots the ratio ASG/BG and ASG/LG for the scapula. There is a significant amount of overlap between the two groups. Goats tend to plot in the upper part of the diagram, reflecting the greater distance between the spine-edge and the glenoid cavity in this species ([28]; [29]). Fig 17 describes the area of the *processus articolaris* and the articulation of the scapula (GLP/LG plotted versus GLP/BG). This combination shows a relatively well-defined separation between the two species, despite some overlap. Fig 18 shows the combination of ASG/SLC with GLP/BG. The goat cluster has lower GLP/BG and higher ASG/SLC values, reflecting the more slender *collum scapulae* of this species, as well as the greater ASG as mentioned above ([28]).

**Fig 19:**
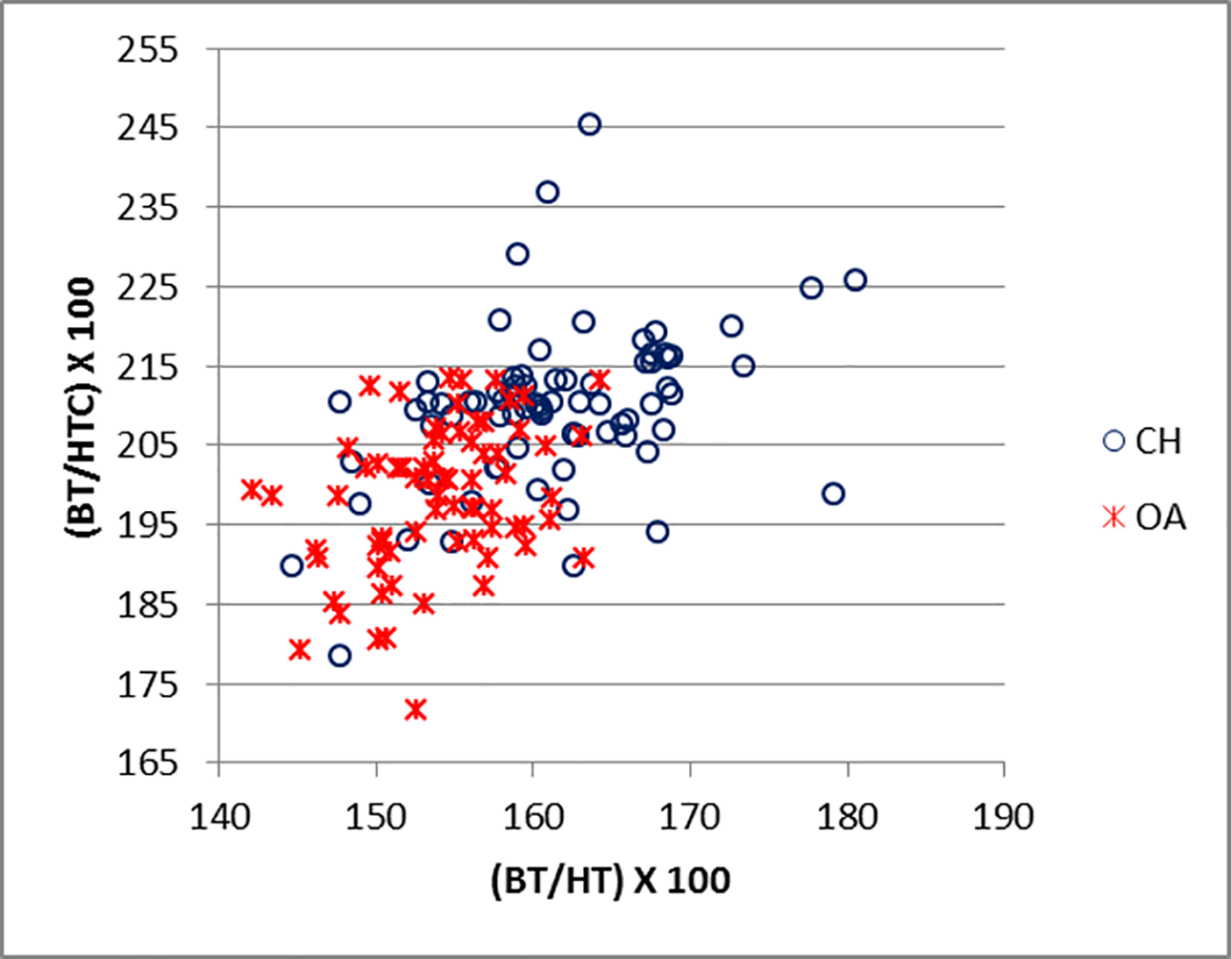
HUMERUS: Ratio between medio lateral breadth of the trochlea (BT) and its height (HT) plotted against medio lateral width of the trochlea (BT) and diameter of the trochlear constriction (HTC). CH = *Capra hircus*; OA = *Ovis aries*.

**Fig 20:**
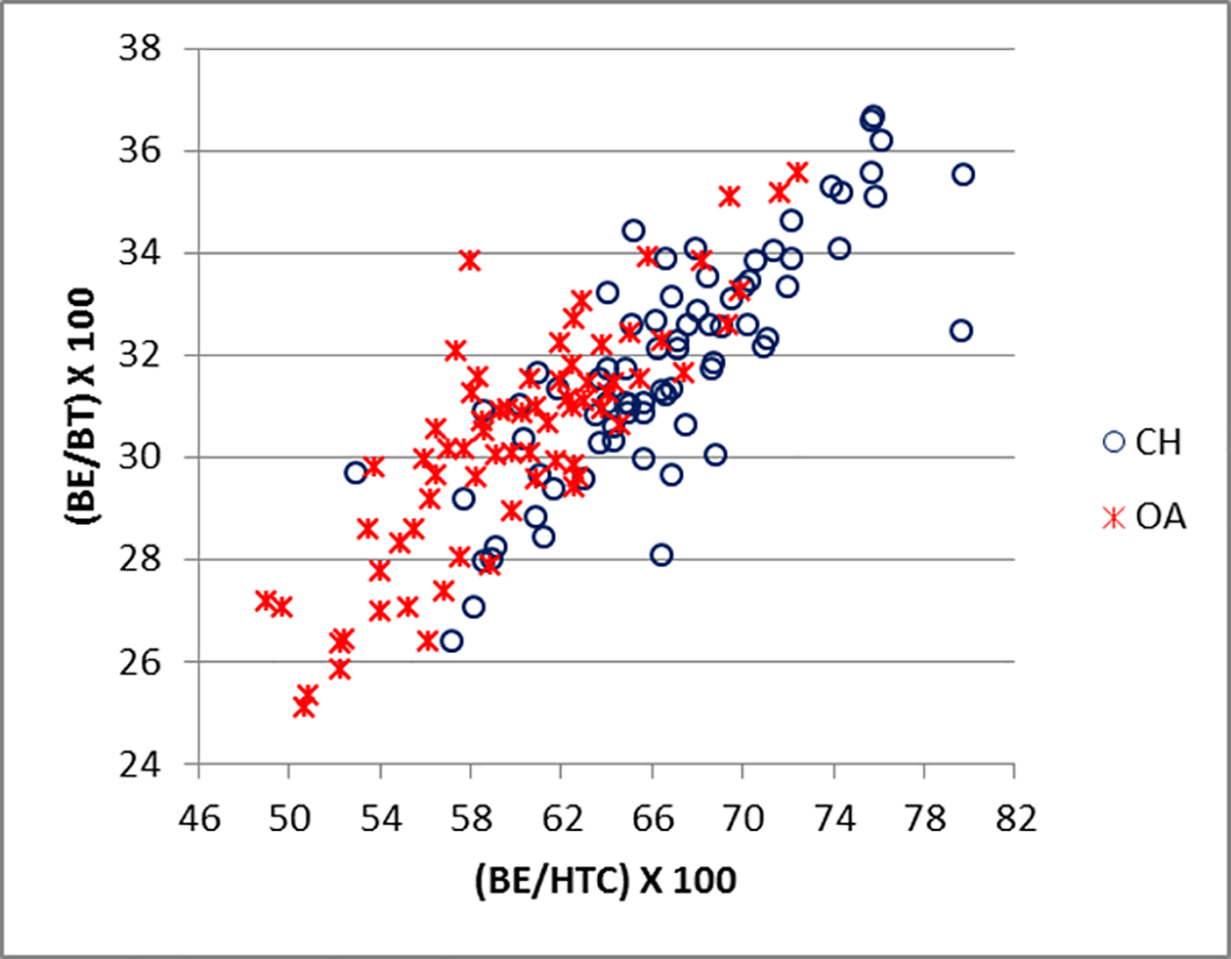
HUMERUS: Ratio between breadth of the *capitulum* (BE) and diameter of the trochlea constriction (HTC) plotted against the ratio between breadth of the *capitulum* (BE) and medio lateral breadth of the trochlea (BT). CH = *Capra hircus*; OA = *Ovis aries*.

**Fig 21:**
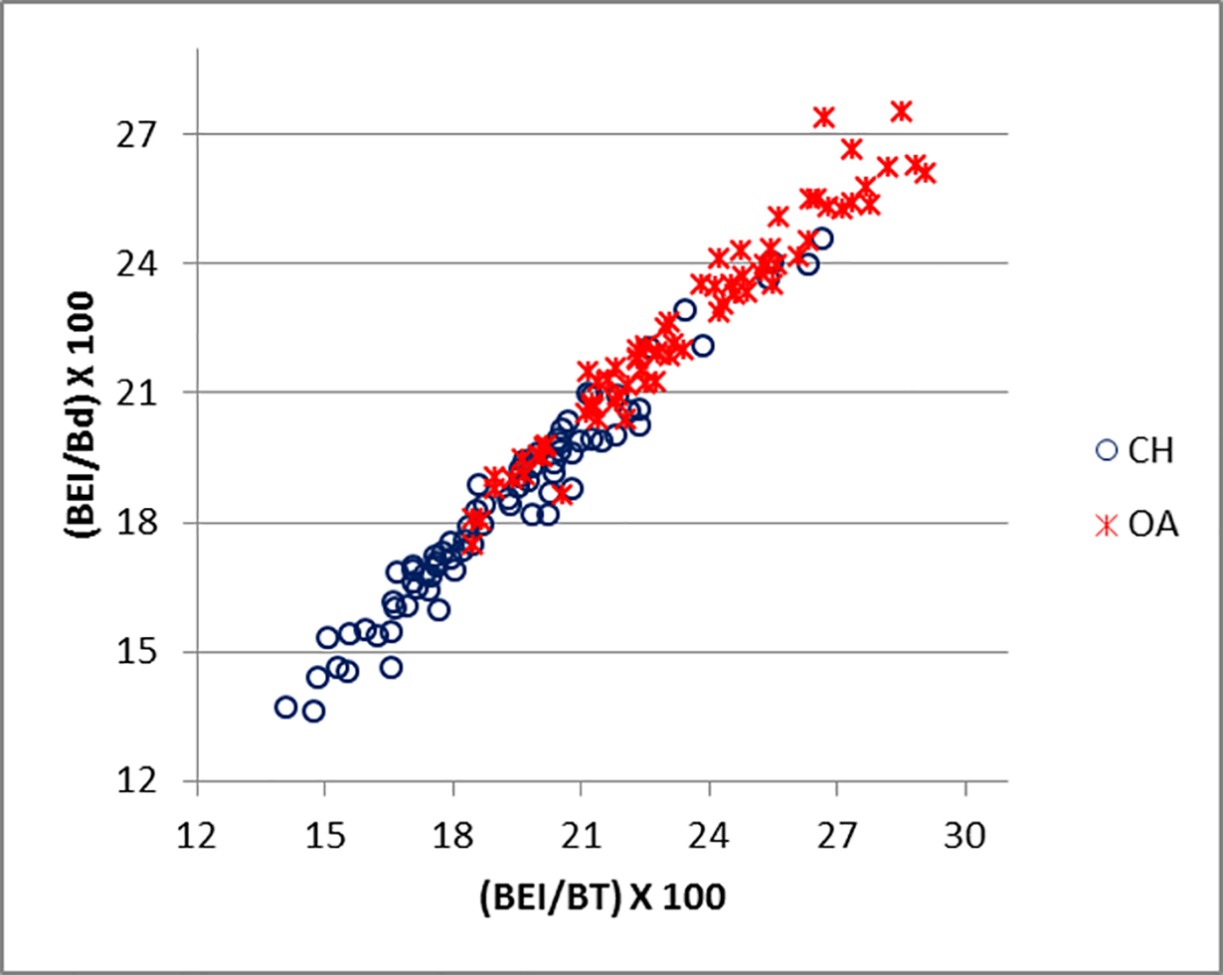
HUMERUS: Ratio between breadth of the *epicondyle lateralis* (BEI) and medio lateral breadth of the trochlea (BT) plotted against the ratio between breadth of the *epicondyle lateralis* (BEI) and breadth of the distal end (Bd). CH = *Capra hircus*; OA = *Ovis aries*.

Fig 19 compares BT/HT and BT/HTC ratios; these describe the shape of the distal trochlea of the humerus. Goats show a greater width of the trochlea in relation to its height: in both species the medial part of the trochlea is higher, but in sheep more so than in goat, giving the goat trochlea an overall more cylindrical shape ([28]; [29]). The ratio BE/HTC plotted against BE/BT (BT was preferred to Bd as this latter has been demonstrated to be an age dependent measurement in pigs [30]) provides a fair separation between the two species (Fig 20), despite some significant overlap. The combination BEI/BT against BEI/Bd (Fig 21) seems to give the best results, probably reflecting the fact that BEI describes a morphological trait considered as a good indicator for sheep/goat discrimination ([28]; [29]; [8]; [4]). The only reason why this may not seem so obvious in Fig 3C is that the two ratios are highly correlated, resulting in a very dense distribution of points; yet almost all goats plot in the lower part of the diagram.

**Fig 22:**
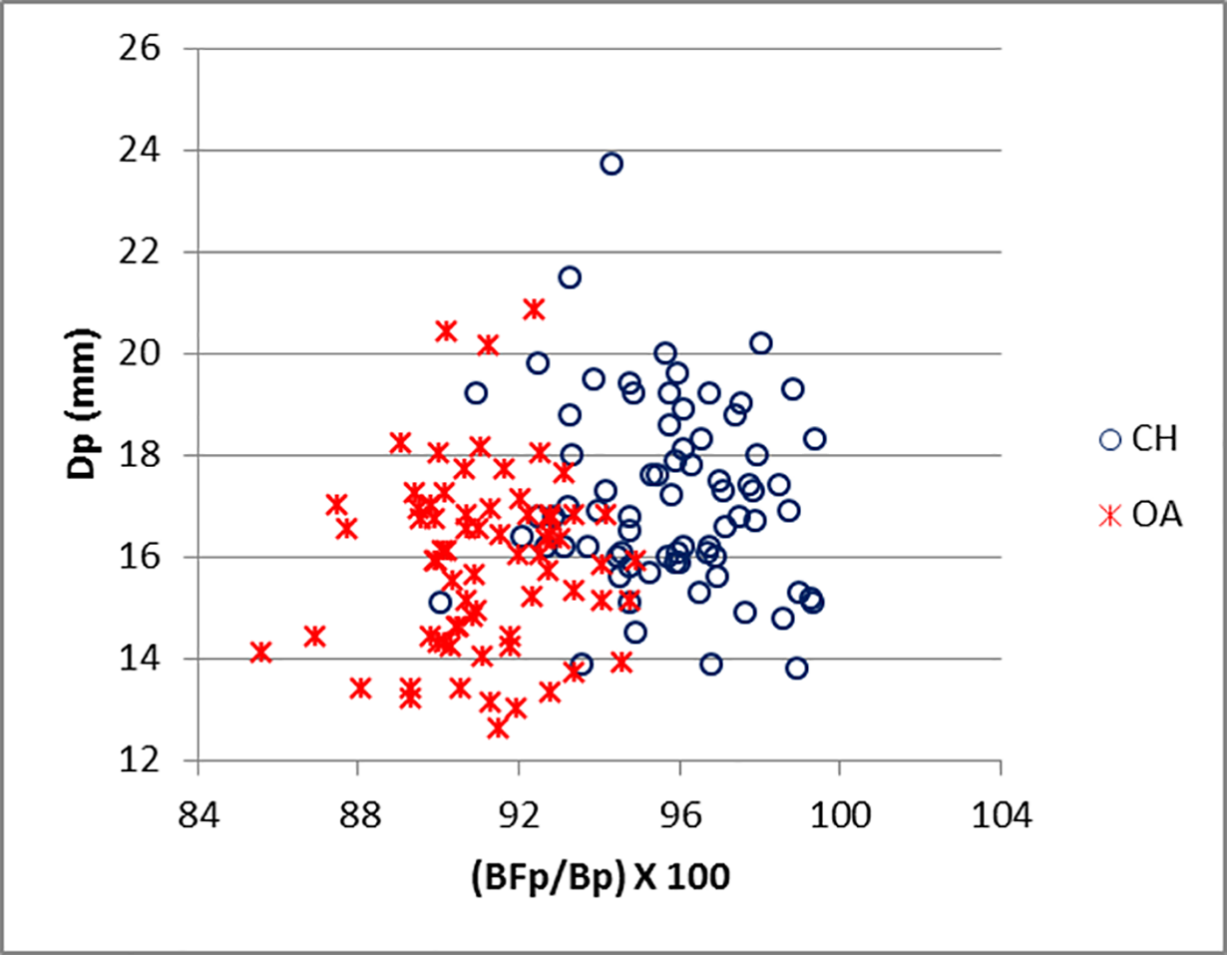
RADIUS: Ratio between breadth of the *facies articularis proximalis* (BFp) and greatest breadth of the proximal end (Bp) plotted against depth of the proximal end (Dp). CH = *Capra hircus*; OA = *Ovis aries*.

The ratio BFp/Bp works well for discriminating between sheep and goat radii (Fig 22). The measurements efficiently describe the presence of a well-developed (in sheep) or less developed (sometimes even absent in goat) lateral bicipital tuberosity on the lateral side of the proximal articular surface ([28]; [8]; [4]).

**Fig 23:**
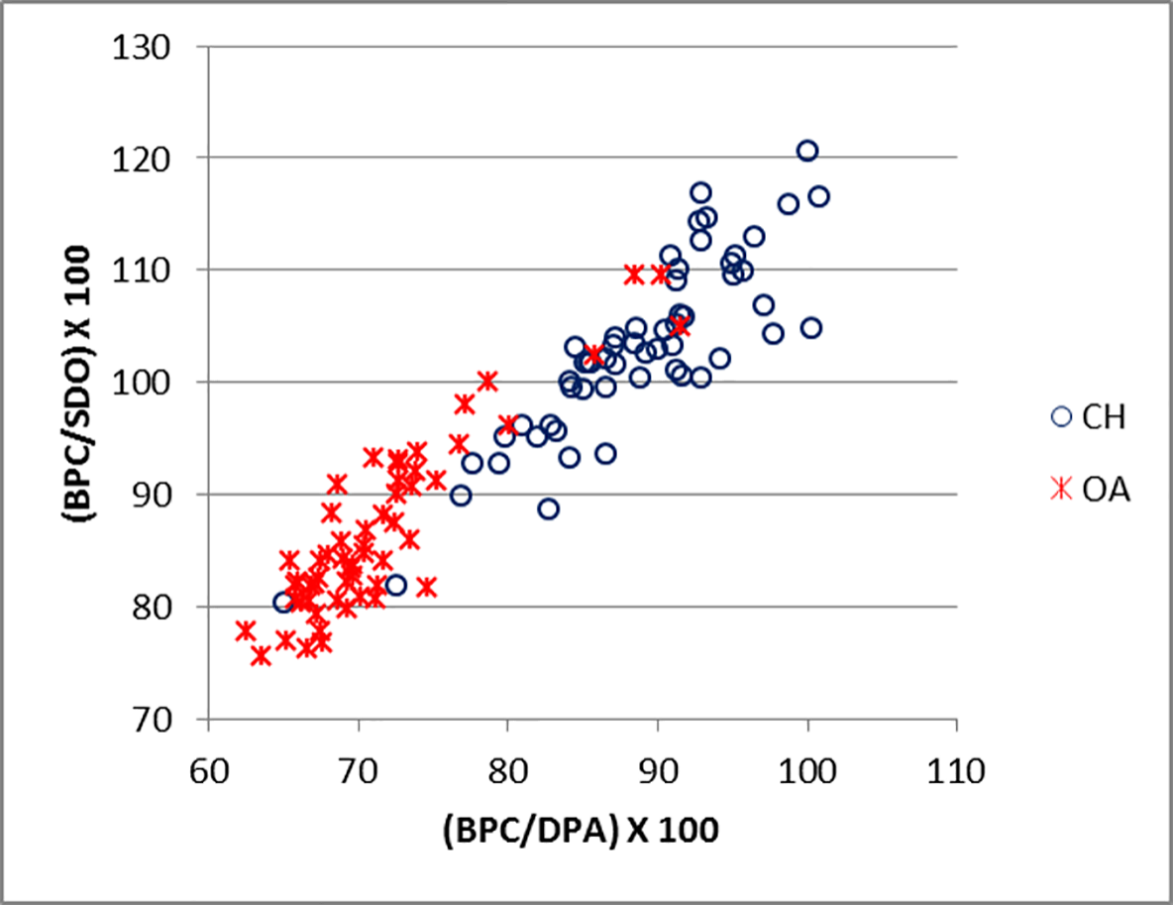
ULNA: Ratio between breadth of the coronoid process (BPC) and depth of the *processus anconaeus* (DPA) plotted against the ratio between the breadth of the coronoid process (BPC) and smallest depth of the olecranon (SDO). CH = *Capra hircus*; OA = *Ovis aries*.

Equally promising are the results obtained from the ulna. The BPC/DPA ratio in particular seems to be useful as it describes the shape of the *anconaeus* process. Fig 23 shows two distinct groups with a small degree of overlap. Goats show higher values in both indices, reflecting how the lateral coronoid process projects more laterally than in sheep ([28]; [1]). Some sheep outliers, however, plot in the middle of the goat distribution. Although these represent a distinct minority, they warn us that identifications must focus on the spread of the distributions rather than individual points, and that multiple lines of evidence and analysis must be used whenever possible.

**Fig 24:**
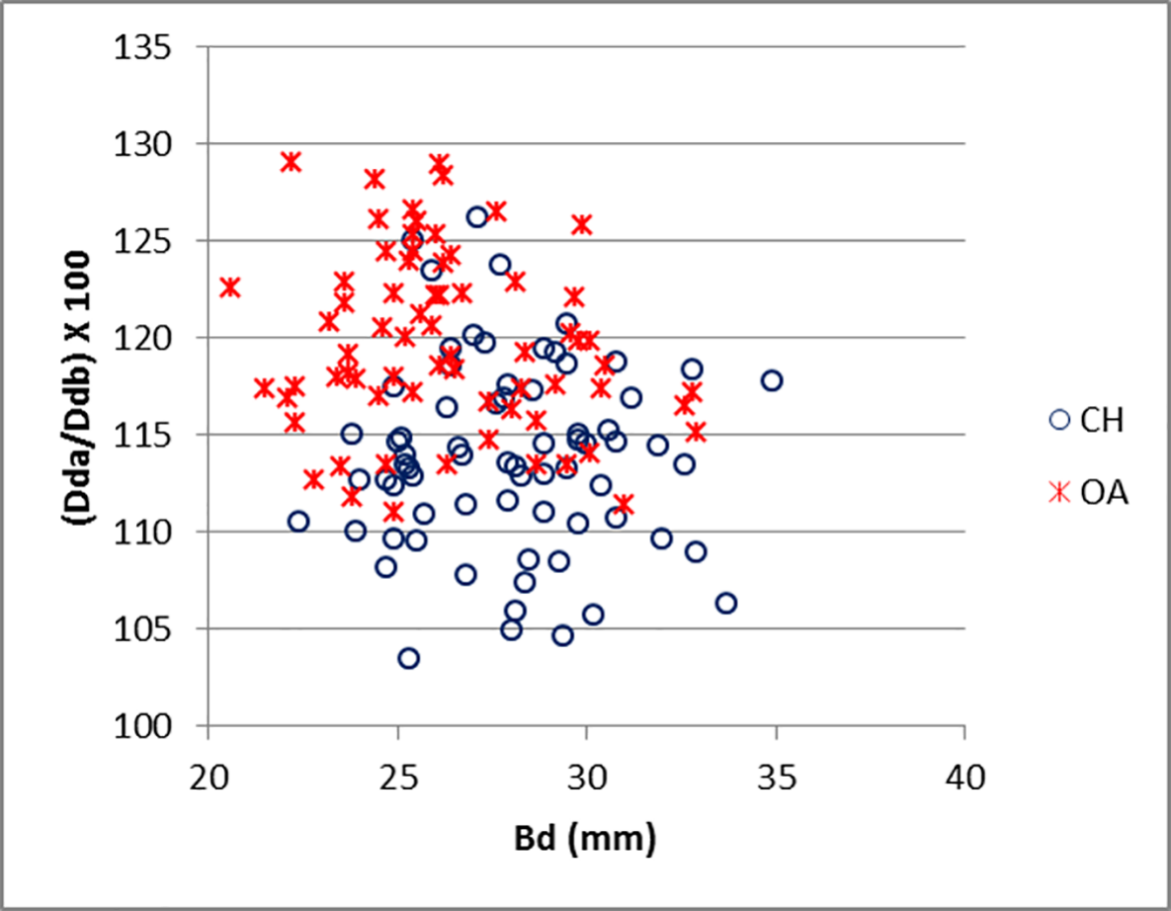
TIBIA: Breadth of distal end (Bd) plotted against the ratio between depth of medial (Dda) and lateral (Ddb) sides. CH = *Capra hircus*; OA = *Ovis aries*.

The results obtained from the distal tibia, disregarded by Boessneck [1] as a useful element for the sheep/goat distinction, confirm the solidity of Kratochvíl’s observations [9] on its diagnostic potential. Fig 24 describes the shape of the distal articulation (though the horizontal axis only expresses size). A certain degree of overlap is present, but the distribution reflects the differences in shape of the distal articulation. As the sheep distal tibia is described as a trapezium and the goat as rectangular ([9]; [8]), the difference between the two measurements is expected to be more marked in sheep, providing a higher ratio value (Dda/Ddb).

**Fig 25:**
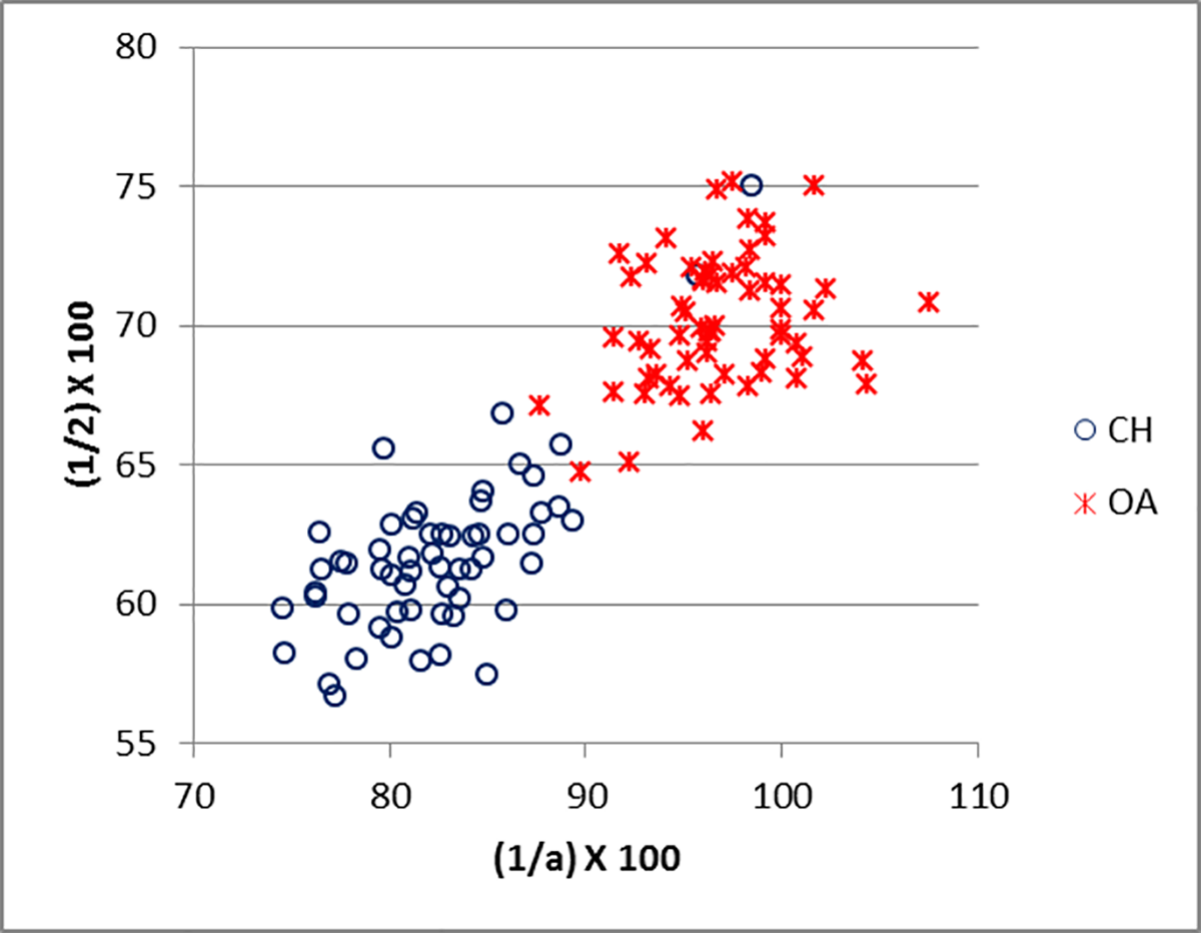
METACARPAL: Ratio between diameter of the medial trochlea (1) and width of the medial condyle (a) plotted against the ratio between diameter of the medial trochlea (1) and diameter of the *verticillus* of the medial condyle (2). The goat outlier is a pigmy goat, as such might have a different morphology. CH = *Capra hircus*; OA = *Ovis aries*.

**Fig 26:**
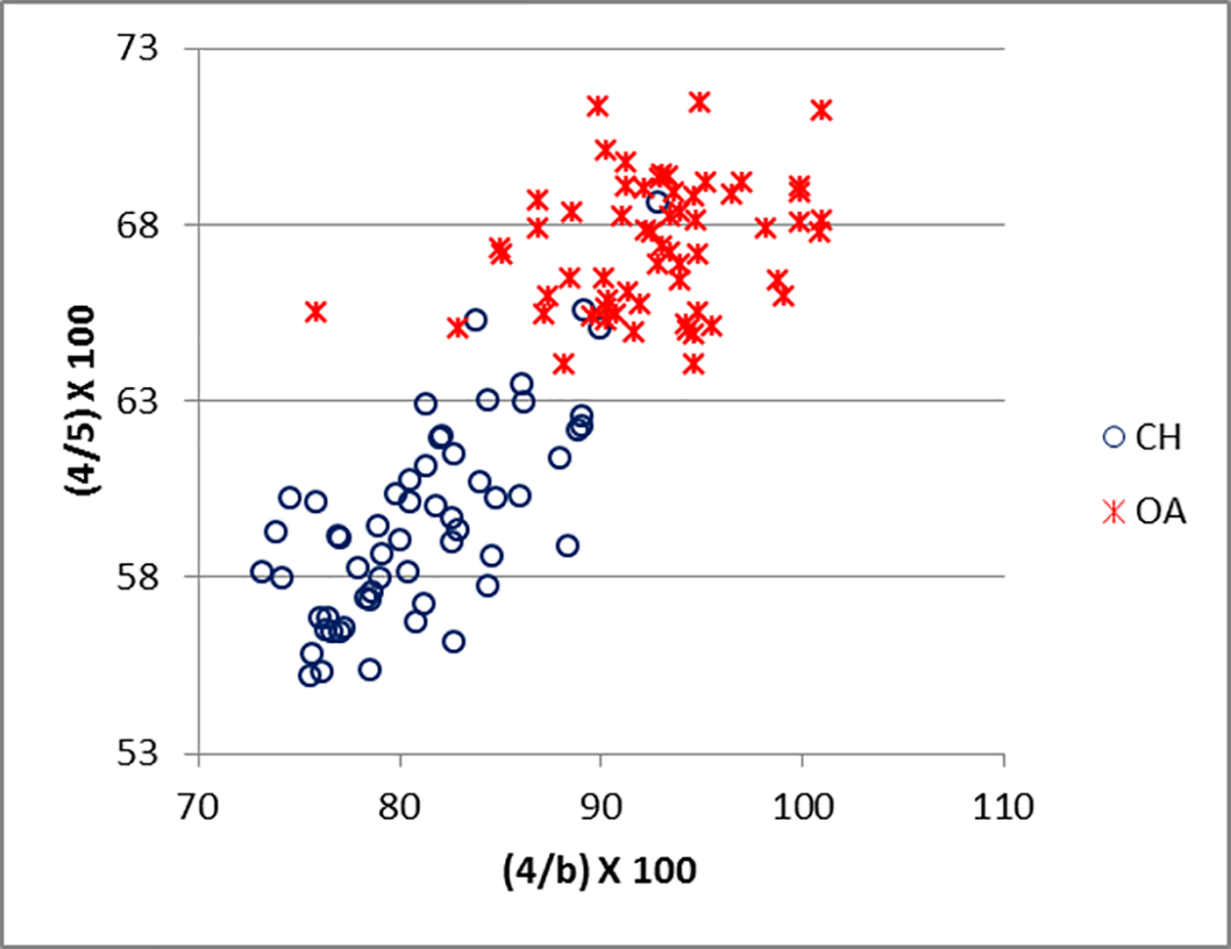
METACARPAL: Ratio between diameter of the lateral trochlea (4) and the width of the lateral condyle (b) plotted against the ratio between diameter of the lateral condyle (4) and diameter of the *verticillus* of the lateral condyle (5). CH = *Capra hircus*; OA = *Ovis aries*.

**Fig 27:**
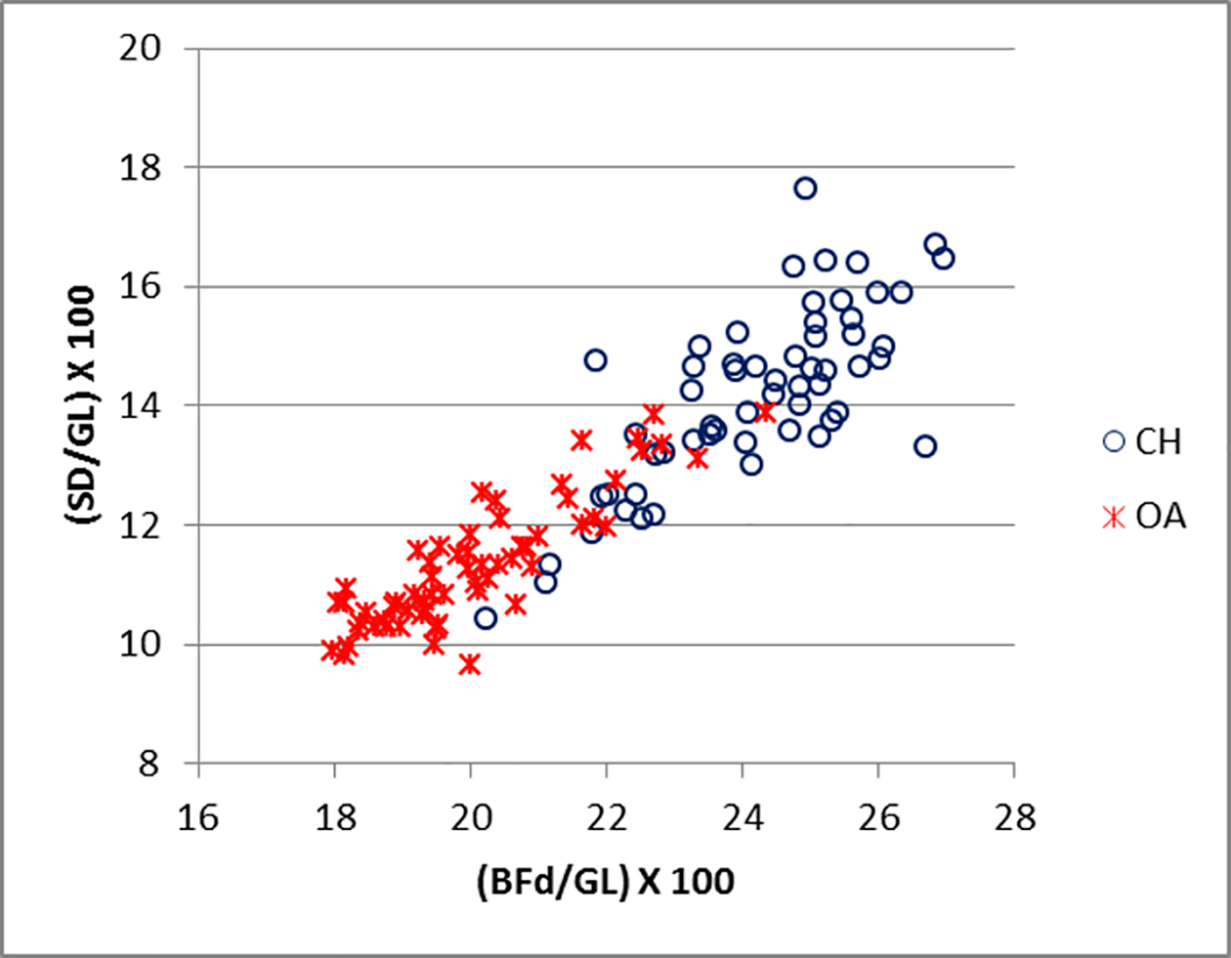
METACARPAL: Ratio between greatest breadth of the distal end (BFd) and greatest length (GL) plotted against the ratio between smallest width of the shaft (SD) and greatest length (GL). CH = *Capra hircus*; OA = *Ovis aries*.

**Fig 28:**
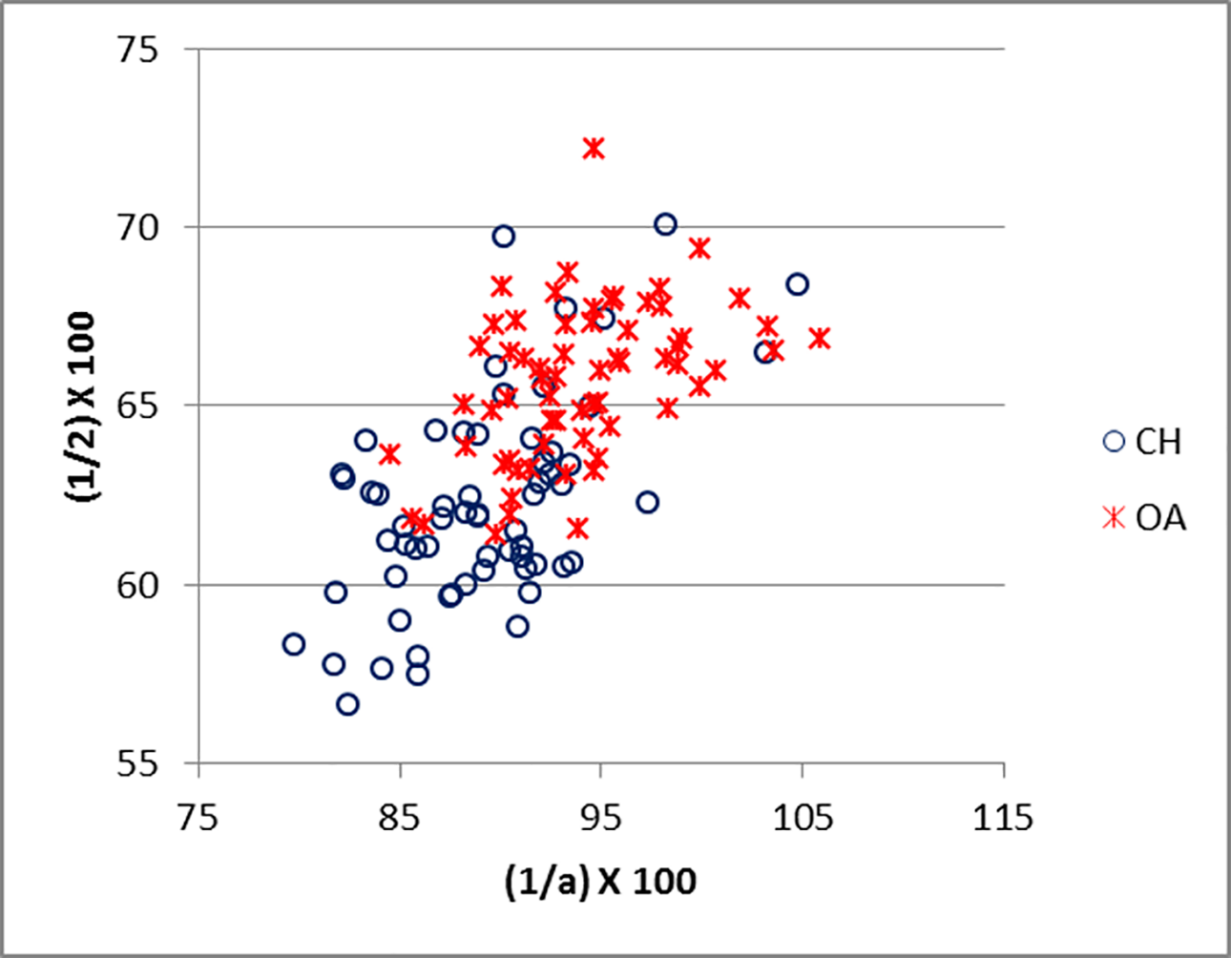
METATARSAL: Ratio between diameter of the medial trochlea (1) and width of the medial condyle (a) plotted against the ratio between diameter of the medial trochlea (1) and diameter of the *verticillus* of the medial condyle (2). CH = *Capra hircus*; OA = *Ovis aries*.

**Fig 29:**
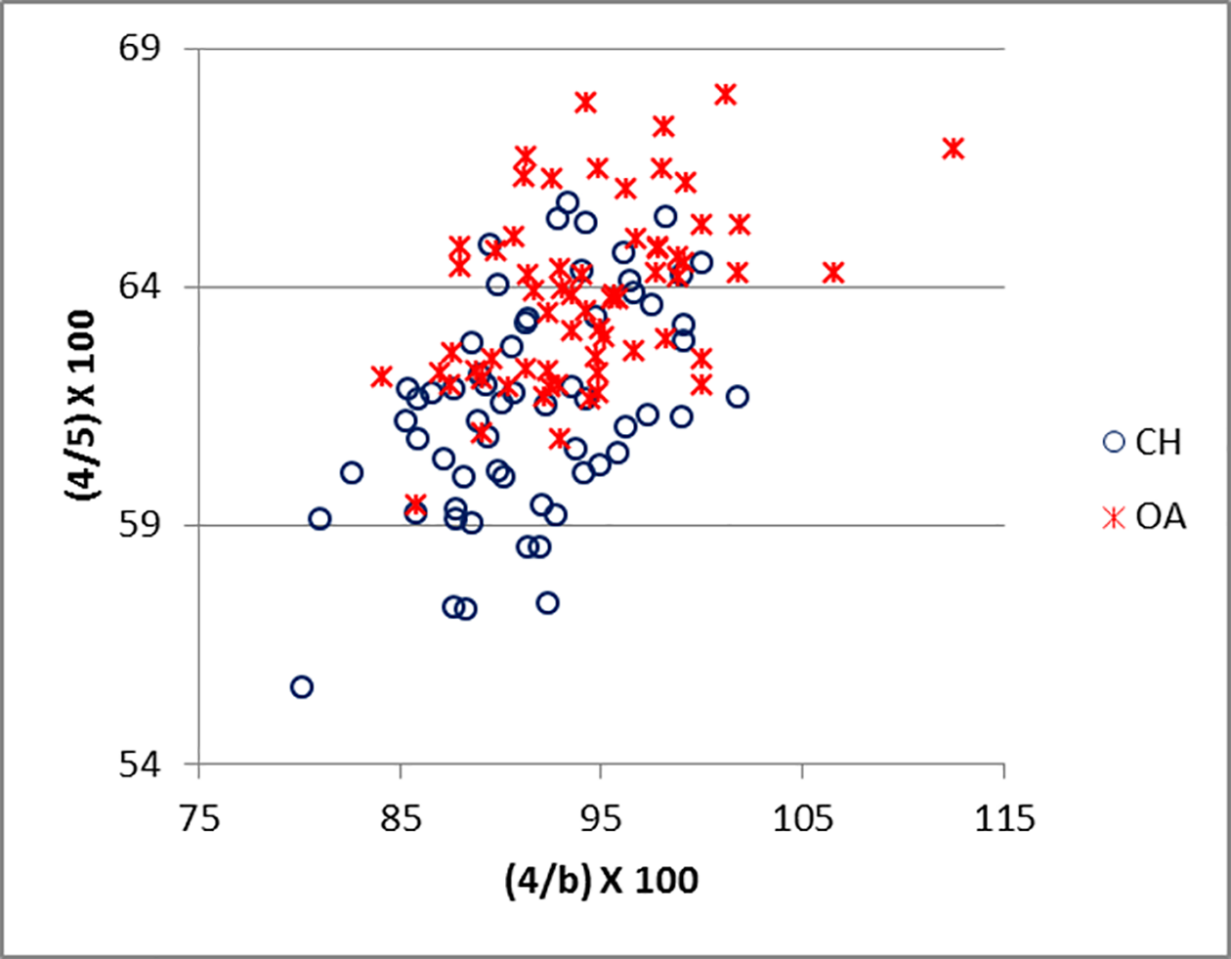
METATARSAL: Ratio between the diameter of the lateral trochlea (4) and the width of the lateral condyle (b) plotted against the ratio between diameter of the lateral condyle (4) and diameter of the *verticillus* of the lateral condyle (5). CH = *Capra hircus*; OA = *Ovis aries*.

**Fig 30:**
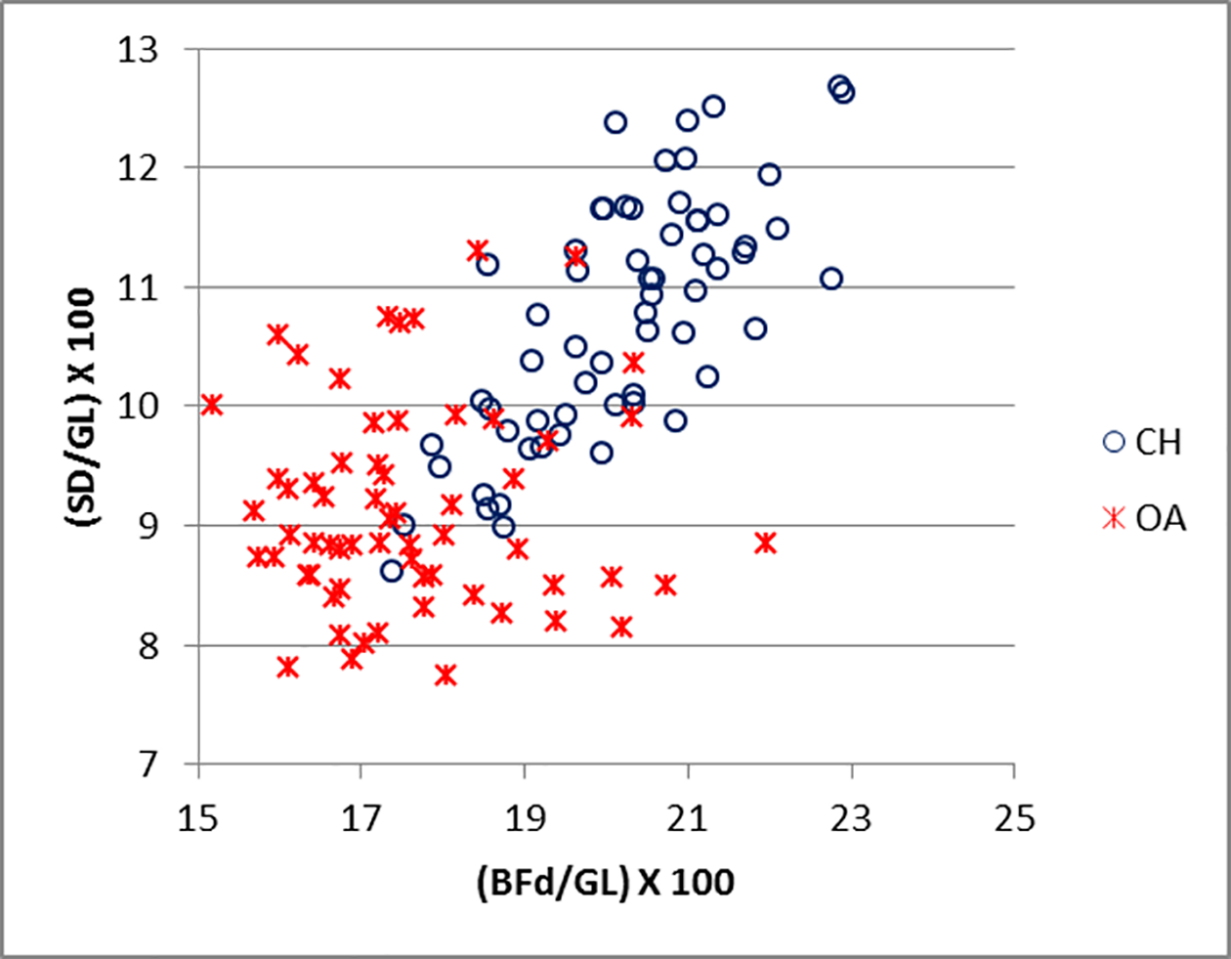
METATARSAL: Ratio between greatest breadth of the distal end (BFd) with greatest length (GL) plotted against the ratio between smallest width of the shaft (SD) and greatest length (GL). CH = *Capra hircus*; OA = *Ovis aries*.

Good results were obtained for metapodials. The separation is clearer for metacarpals (Figs 25–27) than metatarsal (Figs 28–30), confirming previous observations by Boessneck [28] and Payne [13].

For both metapodials the most diagnostic measurements are those taken on the condyles and the *verticilli* of the condyles. A closer look at Figs 25 and 26 and Figs 28 and 29 reveals that the medial condyle measurements (1/a versus 1/2) are slightly more successful in discriminating than the lateral measurements (4b versus 4/5) in both metapodials, confirming what had already been pointed out by previous studies ([28]; [13]; [14]). Nevertheless, when both combinations (1/a *versus* 1/2 and 4/b *versus* 4/5) are used, therefore reflecting the well-known morphological difference of the peripheral part of the trochlear condyles between the two species ([1]; [13]; [4]), two very distinct groups can be recognized. Figs 27 and 30 compare measurements which describe the overall shape of the bone. Two groups are visible, but the separation is less clear-cut: the goat group falls in the upper right part of the graph while the sheep group is located at the bottom right; this distribution reflects the greater slenderness of sheep metapodials compared to goats’ ([28]; [8]).

**Fig 31:**
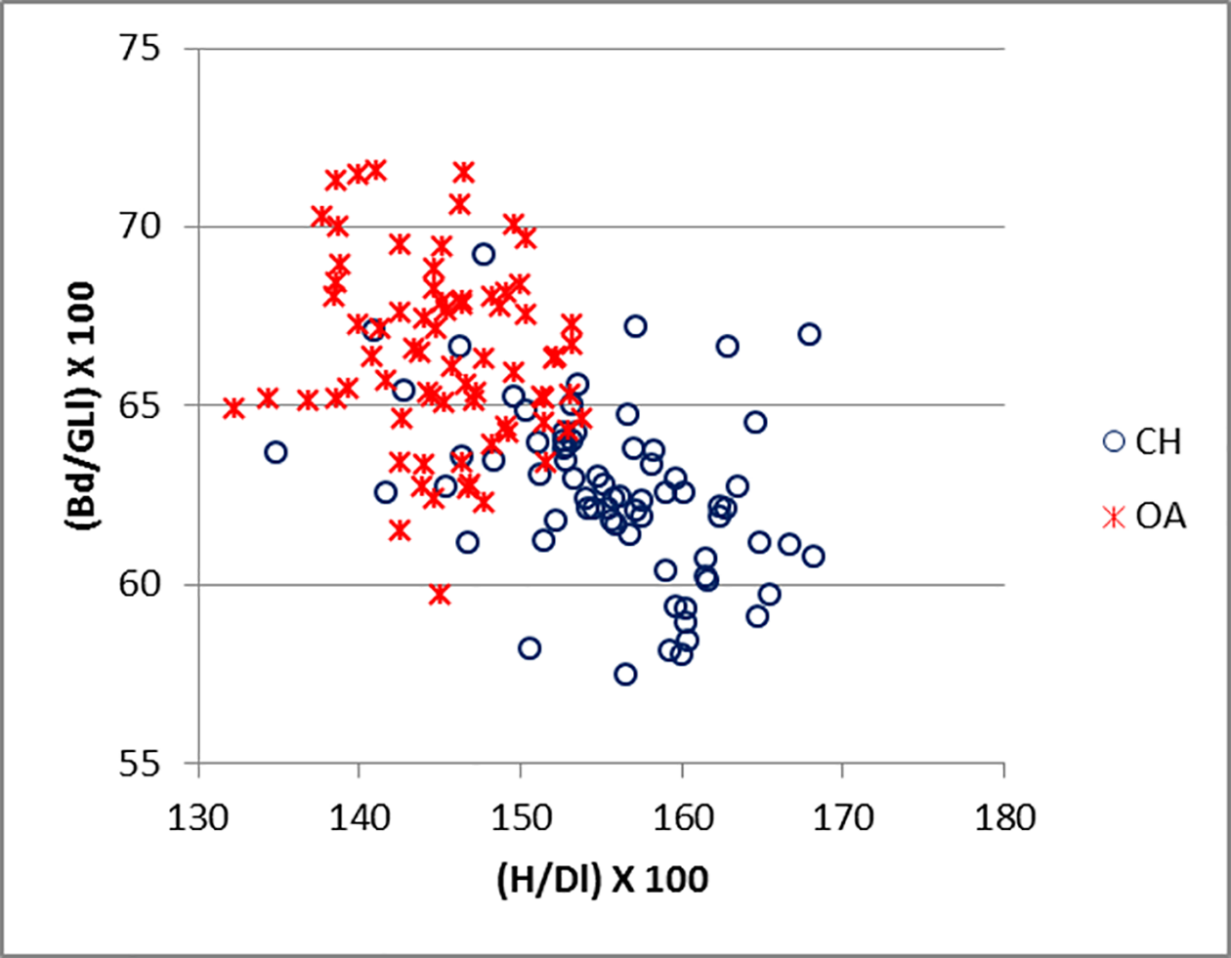
ASTRAGALUS: Ratio between height of the central constriction (H) and greatest depth of the lateral half (Dl) plotted against a ratio between breadth of the distal end (Bd) and greatest length of the lateral half (GLl). CH = *Capra hircus*; OA = *Ovis aries*.

**Fig 32:**
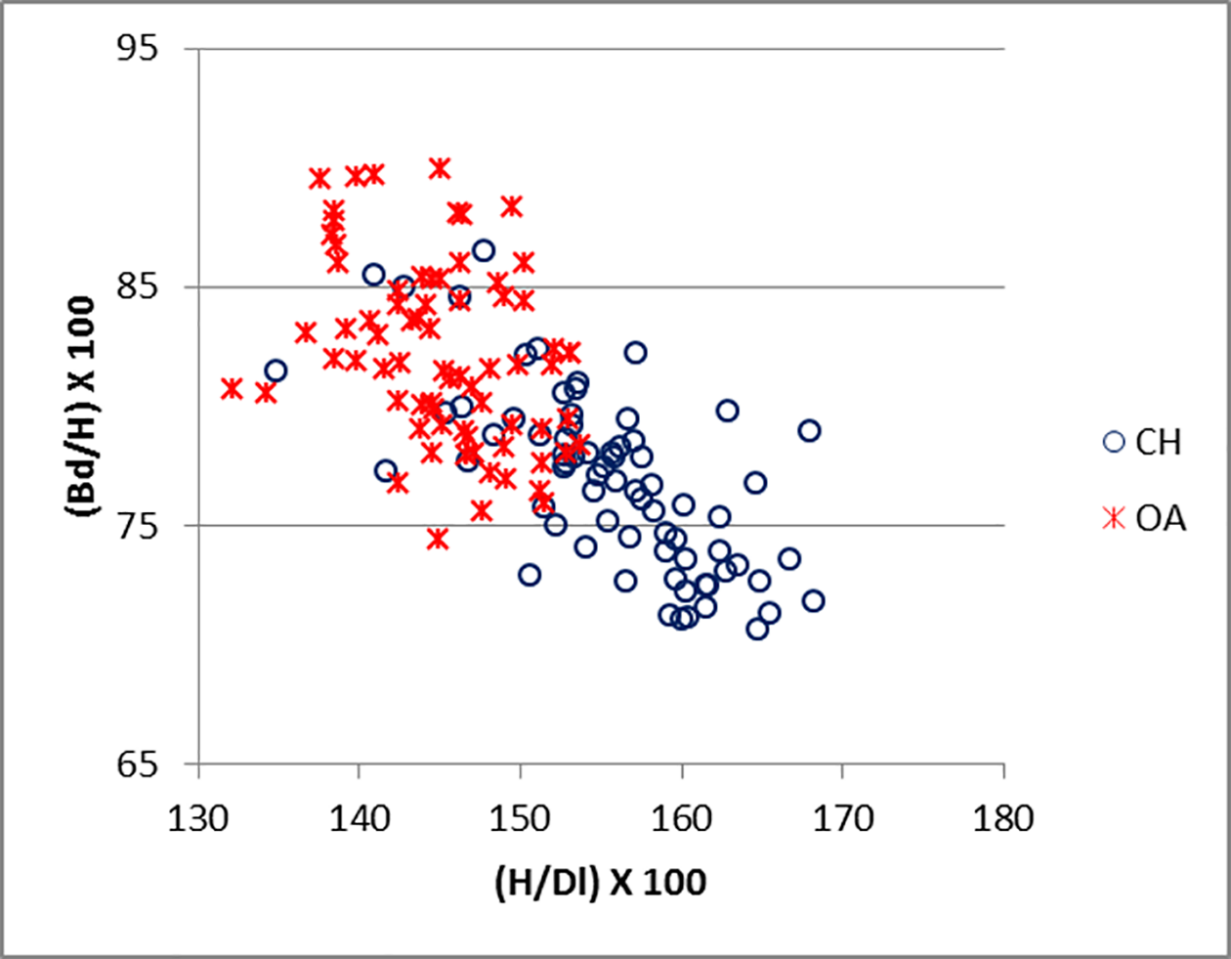
ASTRAGALUS: Ratio between height of the central constriction (H) and greatest depth of the lateral half plotted (Dl) against the ratio between breadth of the distal end (Bd) and height at the central constriction (H). CH = *Capra hircus*; OA = *Ovis aries*.

**Fig 33:**
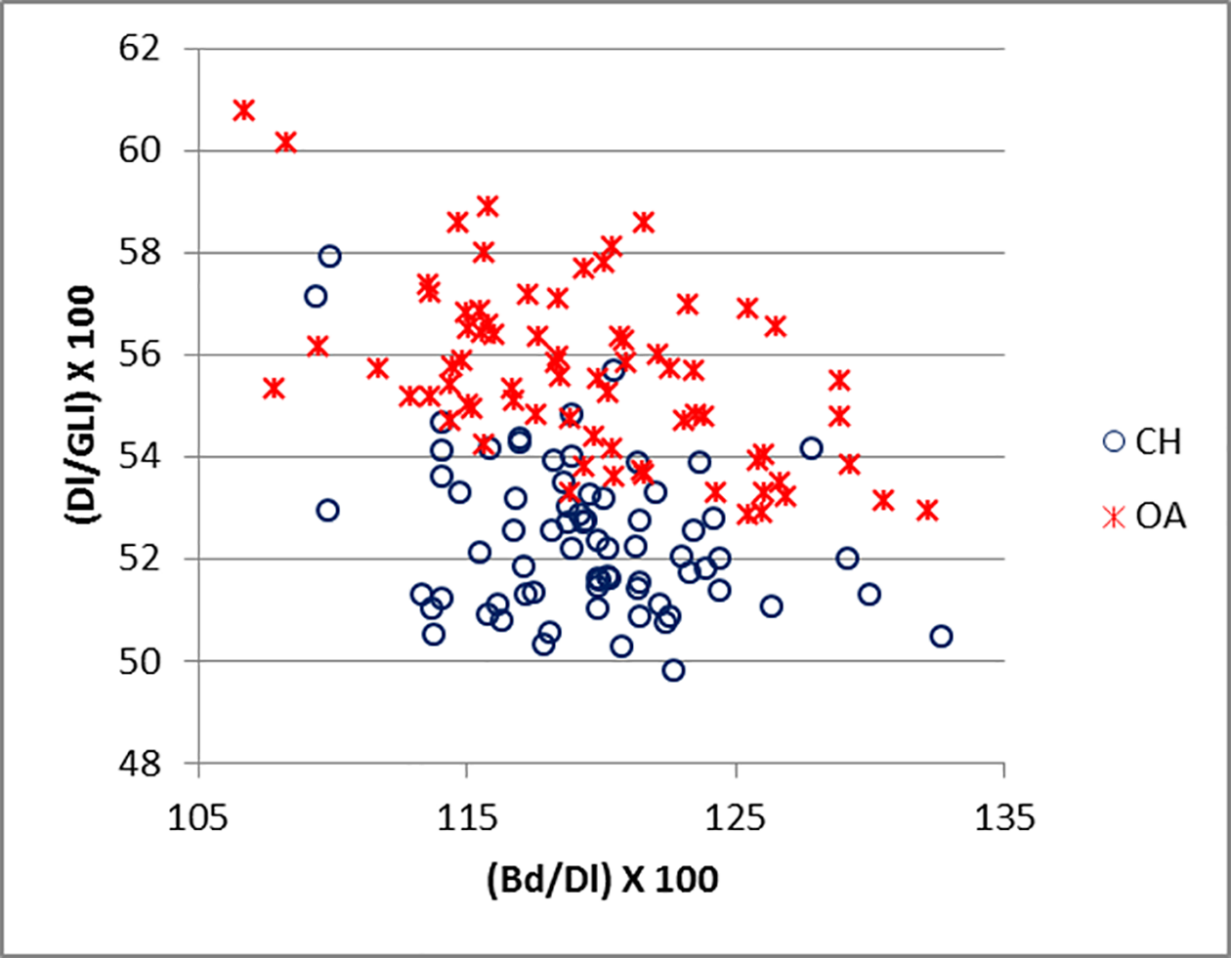
ASTRAGALUS: Ratio between breadth of the distal end (Bd) and greatest depth of the lateral half (Dl) and the ratio between greatest depth of the lateral half (Dl) and greatest length of the lateral half (GLl). CH = *Capra hircus*; OA = *Ovis aries*.

**Fig 34:**
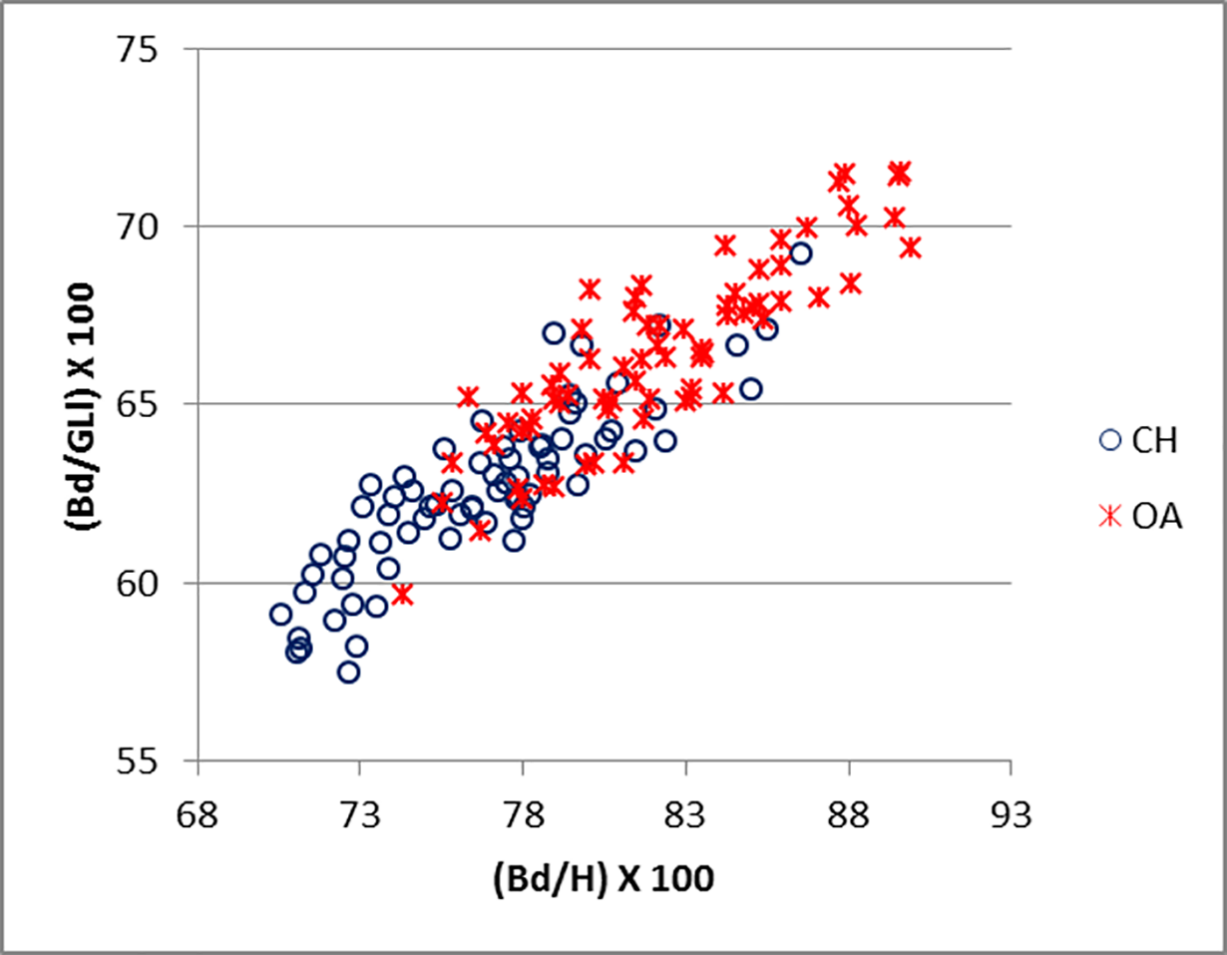
ASTRAGALUS: Ratio between breadth of the distal end (Bd) and height at the central constriction (H) plotted against the ratio between breadth of the distal end (Bd) and greatest length of the lateral half (GLl). CH = *Capra hircus*; OA = *Ovis aries*.

The most successful ratios for the astragalus include measurements (GLl, Dl and Bd) that have also been adopted by Davis [10] and form the basis of a metrical method for separating sheep and goat astragali. In addition, the new measurement H, has also been proven to have some diagnostic value. Fig 31 shows (H/Dl versus Bd/GLl) that the separation between the two species is determined by both axes, with a major influence exercised by H/Dl. This reflects the fact that the sulcus at the middle of the trochlea is usually deeper in sheep than goat [1]. In addition, Dl in goat is influenced by the presence of an articular ridge which projects more obliquely in a distal direction, while in sheep it is less pronounced and more horizontally oriented ([1]; [4]). On the other hand sheep show higher Bd/GLl scores, reflecting the more robust shape (wider in relation to the height) of the bone of this species. Fig 32 represents a modified version of Fig 31 where H replaces GLl. The pattern is similar, with the greater separation occurring on the horizontal axis. A description of the complete shape of the bone is given by Fig 33, which includes all three main dimensions (breadth, depth and length). The two groups fall into two different areas of the plot with only a few specimens overlapping. The distinction is almost entirely due to Dl/GLl, with the more robust astragali of sheep plotting in the upper part of the graph. A fair separation between the two groups is shown also in Fig 34 (ratio Bd/H against Bd/GLl) where, once again, sheep tend to plot towards the top and goats the bottom.

**Fig 35:**
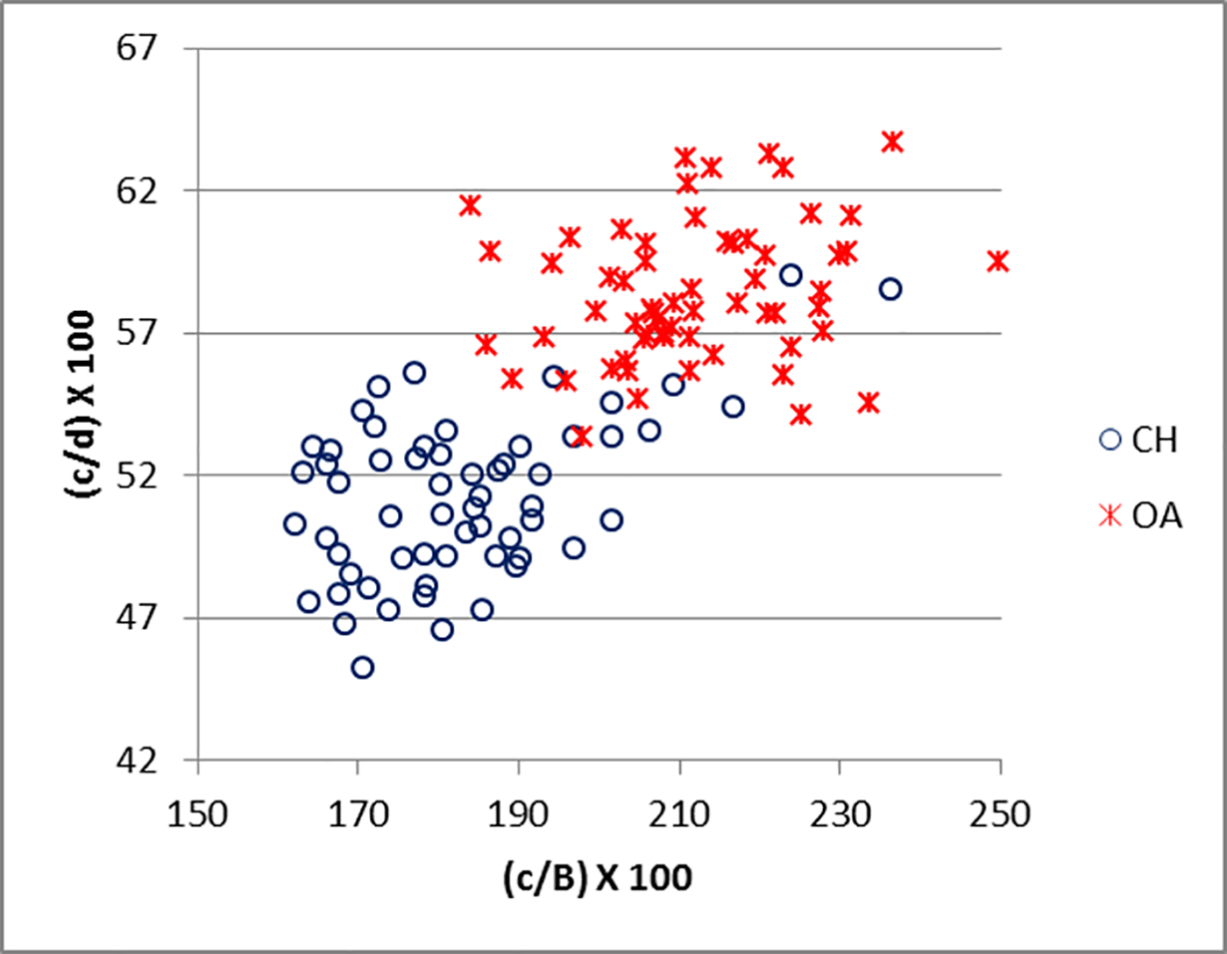
CALCANEUM: Ratio between length (c) and breadth (B) of the articular facet of the *os malleolare* plotted against the ratio between length of the articular facet of the *os malleolare* (c) and the length taken from the articular facet of the *os malleolare* to the end of the articulation-free part of the process (d). CH = *Capra hircus*; OA = *Ovis aries*.

**Fig 36:**
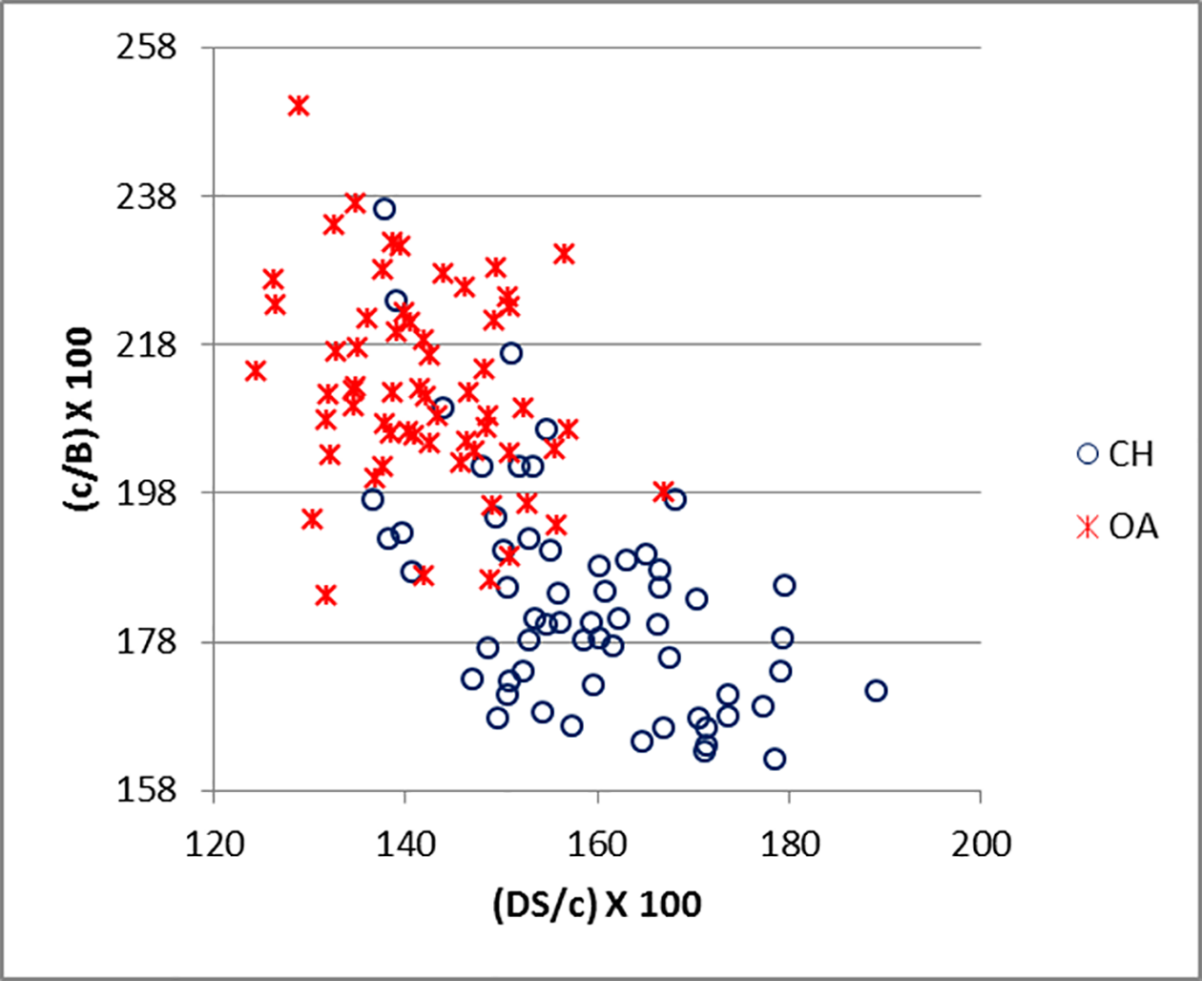
CALCANEUM: Ratio between greatest depth of the *substentaculum tali* (DS) and length of the articular facet of the *os malleolare* (c) plotted against the ratio between length (c) and the breadth (B) of the articular facet of the *os malleolare*. CH = *Capra hircus*; OA = *Ovis aries*.

**Fig 37:**
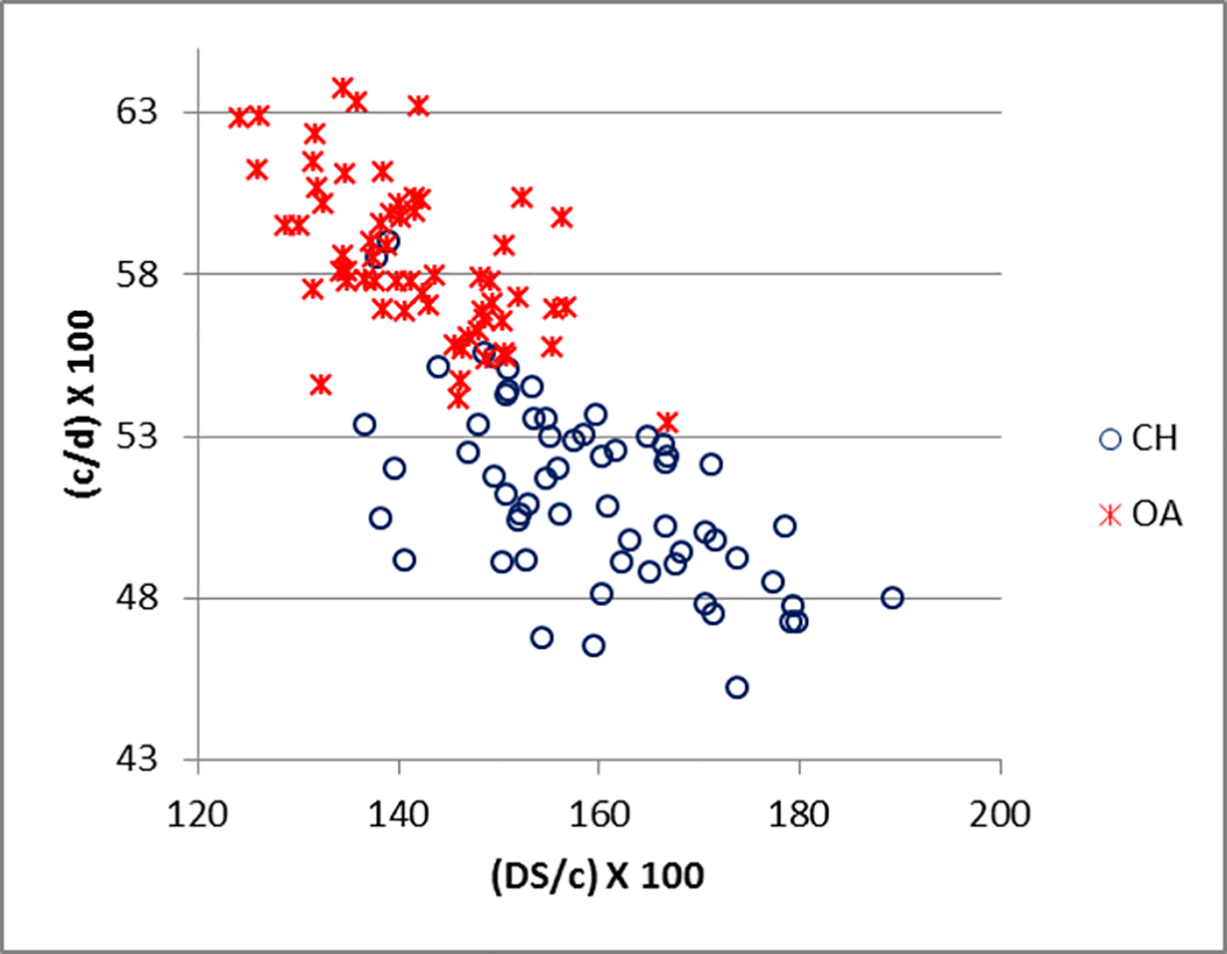
CALCANEUM: Ratio between greatest depth of the *substentaculum tali* (DS) and length of the articular facet of the *os malleolare* (c) plotted against the ratio between length of the articular facet of the *os malleolare* (c) and length taken from the articular facet of the *os malleolare* to the end of the articulation-free part of the process (d). CH = *Capra hircus*; OA = *Ovis aries*.

Fig 35 demonstrates how the measurements suggested by Boessneck [28] (in this study c, d and B) for the calcaneum can be useful and, when plotted together, they provide good separation. These ratios clearly mirror the fact that the length of the articular facet for the *os malleolare* on the lateral process is greater than half of the entire process (c/d) in sheep, while in goat it is smaller ([28]; [4]). Measurement B describes the difference between the articular facet of the *os malleolare*, which in sheep is of greater length than width, whereas in goat it is of greater width than length ([28]; [1]; [4]). A good degree of separation was also obtained when c and d were plotted against DS/c (Figs 36 and 37).

**Fig 38:**
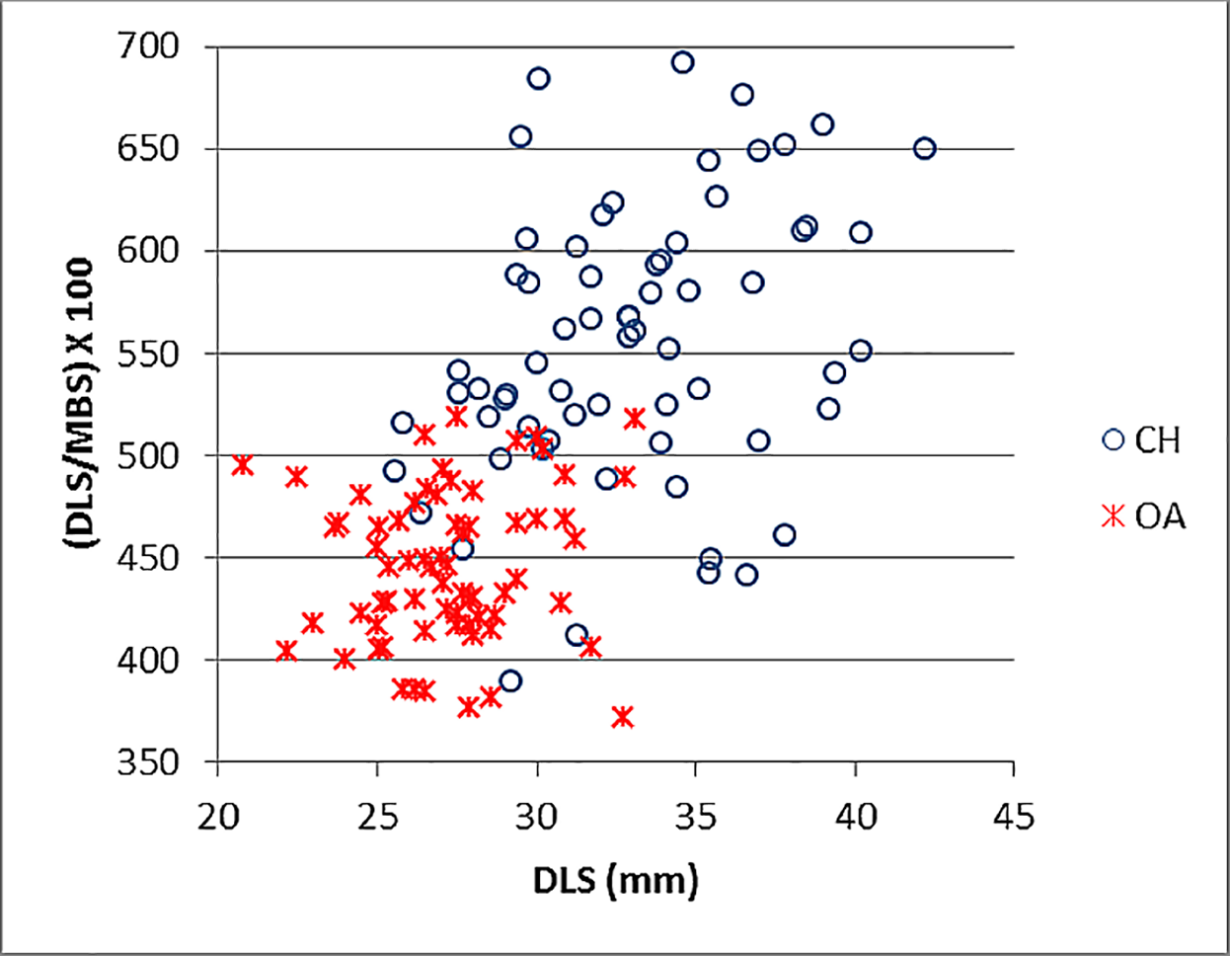
3^rd^ PHALANX: Diagonal length of the sole (DLS) plotted against the ratio between diagonal length of the sole (DLS) and the middle breadth (MBS). CH = *Capra hircus*; OA = *Ovis aries*.

Since one of the main morphological differences between sheep and goat for the third phalanx is represented by the shape of the sole (in sheep it is more curved and less triangular, while in goat it is shaped almost like an isosceles triangle), the ratio MBS/DLS was plotted against DLS. Fig 38 shows that these measurements are very effective as they provide good separation between the two groups.

Mann Whitney tests of significance were run on the adopted indices, using the taxa as a grouping variable. This non-parametric test is an equivalent to the more commonly used independent t-test (parametric test) but requires fewer assumptions ([31]: 540). It was carried out with the purpose of checking whether differences between the two groups were statistically significant. The results are provided in S3 Table, along with the Median (middle score of a set of ordered observations), a more appropriate value than the mean for non-parametric tests ([31]: 789), and Effect Size values (objective and standardized measure of the magnitude of an observed effect; [31]: 785). A Bonferroni adjustment was applied in order to avoid Type I Error (i.e. the running of many consecutive paired tests can lead to finding more significant differences than there actually are). S3 Table clearly shows that all metrical indices (apart from 4/b in the metatarsal and Bd/Dl in the astragalus. BE/BT and BE/Bd also provide no significant differences when a Bonferroni’s correction is applied) have highly significant scores (*p* value<0.001) thus confirming the significance of the separation between the two groups.

In order to test if statistical significant differences were present also when two biometrical ratios were compared simultaneously, a Manova test was carried out for every combination of ratios used. As S4 Table shows, all the F values are greater than 1 and the related *p* values are all significant, confirming that the differences between the modern sheep and goat samples, even when multiple ratios are combined, are statistically significant.

### 3.2 Discriminant analysis

Standard Discriminant Analysis ([32]: 395) was run in order to see if, by including all measurements at once, we could find a means of maximising the separation between the two species. In addition, this method runs a re-classification of the known cases, thus testing the validity of the discriminating criteria ([33]: 105).

The analysis was undertaken for each element individually, using species as the grouping variable and the chosen measurements as the independent variables. Output options were set to give case-by-case discriminant data, so that the identification result for each individual specimen was obtained as well as a summary table. A plot of all cases was also produced using the canonical discriminant individual scores as the vertical axis. Prior to the analysis, a method of standardisation was applied to the raw data (following Davis [34]: 523) in order to exclude the size factor, which was not relevant to this research and can sometimes cloud the results. This was done by dividing individual measurements by the total for that anatomical element and multiplying the result by 100.

S5 Table presents a summary of the results obtained. The percentage of correct re-attributions for each species is provided, along with the overall rate of successful re-attributions. S6 Table shows the Correlation Coefficients for each variable/measurement for each anatomical element considered. The Correlation Coefficient value represents the extent to which the variable participates in the function; in other words, it quantifies the extent to which a particular measurement contributes to the separation between the two taxa. Variables with higher positive/negative coefficients are those that contribute the most. The positive or negative coefficients attest that variables/measurements have the opposite effect on the function: this indicates that two different contributions are made to the differentiation process [31]).

**Fig 39:**
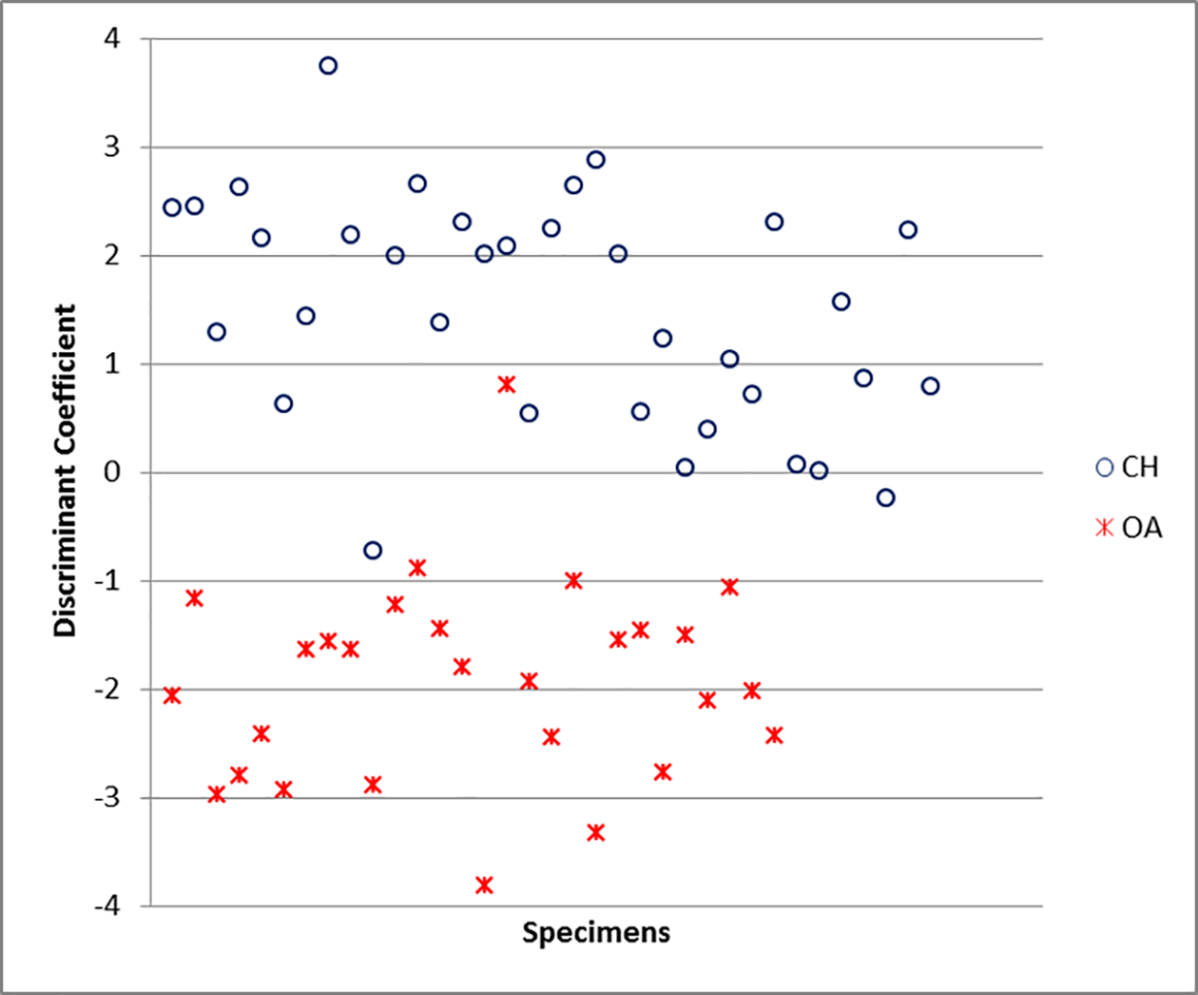
HORNCORE: Scatterplot of the individual discriminant scores. CH = *Capra hircus*; OA = *Ovis aries*.

For the horncores, the function is highly influenced by the length of the horncore (E) and the shape of the base taken either at the middle (C and D) or at the base of the bone (A and B). The variable F has been excluded by the program because it correlates too highly with one or more other variables (S6 Table). The percentage of originally grouped specimens correctly classified by the Discriminant Analysis for this element is 95.2% (S5 Table), a substantially high value as it means that, on a hypothetical sample of 100 unknown specimens, 95 would be correctly identified. When the Discriminant Individual Scores of each modern specimen are visually displayed (Fig 39), two almost completely distinct groups can be identified, confirming that DA can assign horncores to one of the two species with a high degree of success.

**Fig 40:**
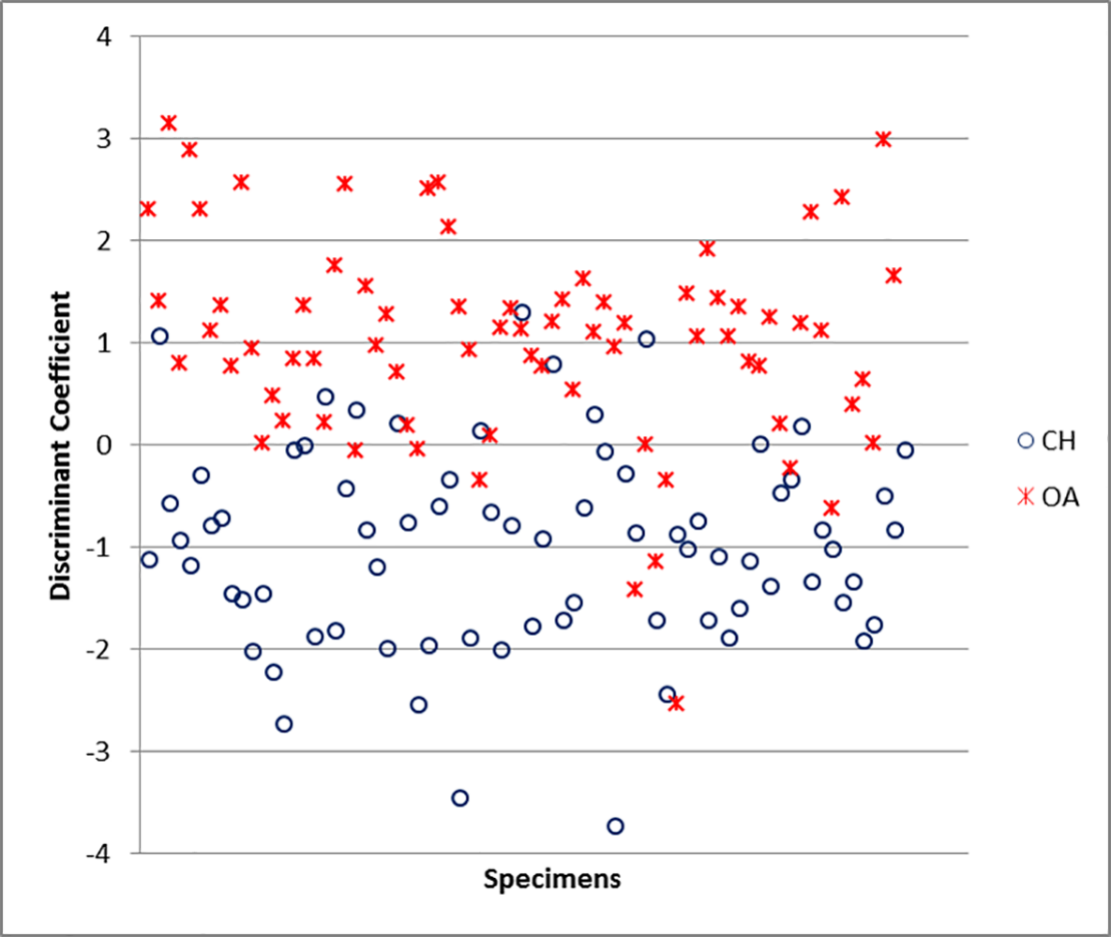
SCAPULA: Scatterplot of the individual discriminant scores. CH = *Capra hircus*; OA = *Ovis aries*.

For the scapula, the variables which mostly contribute to the separation are GLP and ASG (S6 Table). The percentage of correct identification is 86.4% (S5 Table), predictably lower than for horncores, but still encouraging. The relative success of the discriminant function becomes visually clear in Fig 40: despite an area of overlap, most of the plot area is mainly occupied by only one of the two species (sheep at the top, goats at the bottom).

**Fig 41:**
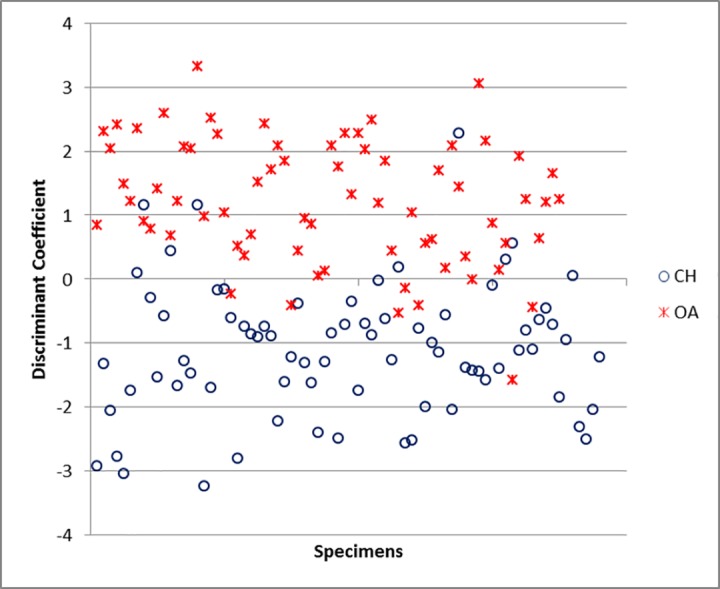
HUMERUS: Scatterplot of the individual discriminant scores. CH = *Capra hircus*; OA = *Ovis aries*.

For the humerus, the variables which contribute the most to the separation of sheep from goat are BEI, BE and BT (and, to a lesser degree, HTC and HT) (S6 Table).The percentage of cases correctly classified for the humerus is 88.4% (S5 Table), a good score considering that measurements taken on the distal humerus have previously been considered of no use for sheep/goat discrimination [11]. When the Individual Discriminant Scores are plotted (Fig 41) some area of overlap can be seen, but there are also areas of the graph in which, by and large, only one *taxon* can be found.

**Fig 42:**
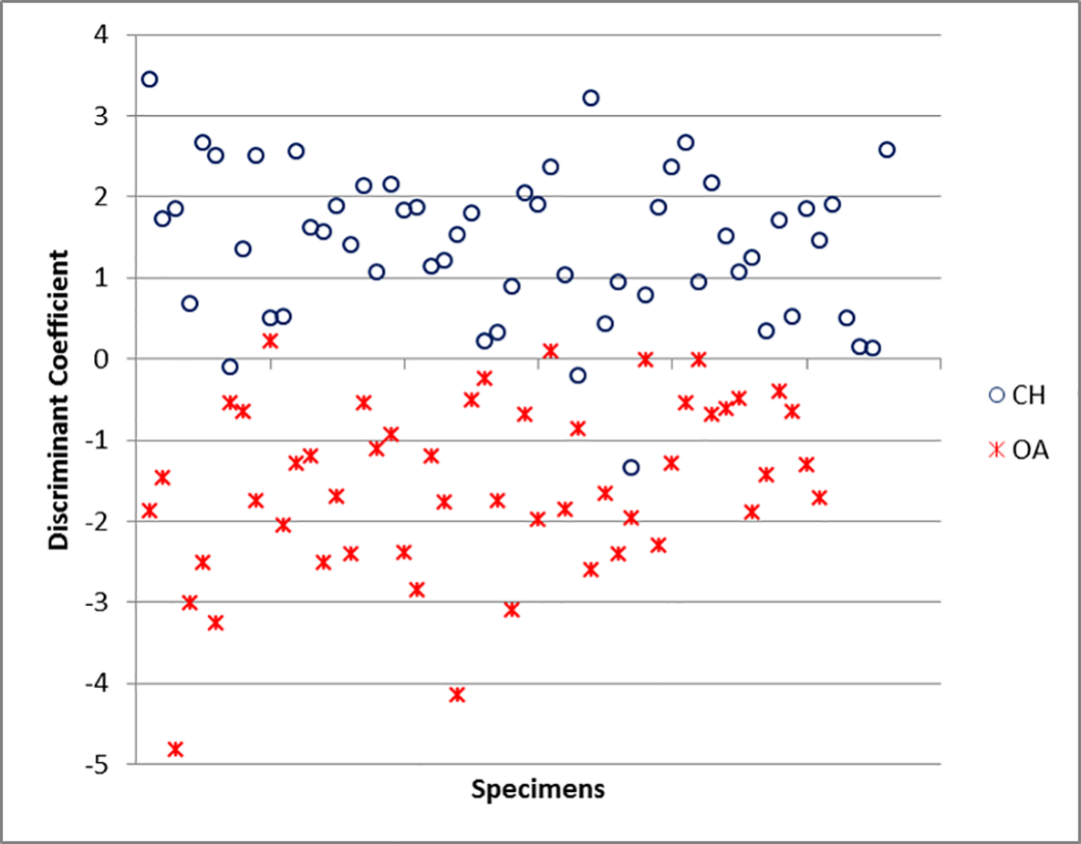
RADIUS: Scatterplot of the individual discriminant scores. CH = *Capra hircus*; OA = *Ovis aries*.

Bp and GL are the most powerful variables for the function when the radius is considered (S6 Table). The classification rate for this element is 93.5%, a promising score (S5 Table). The scatterplot of the Individual Discriminant Coefficients (Fig 42) shows that, despite the presence of a few overlapping specimens, two groups are clearly visible.

**Fig 43:**
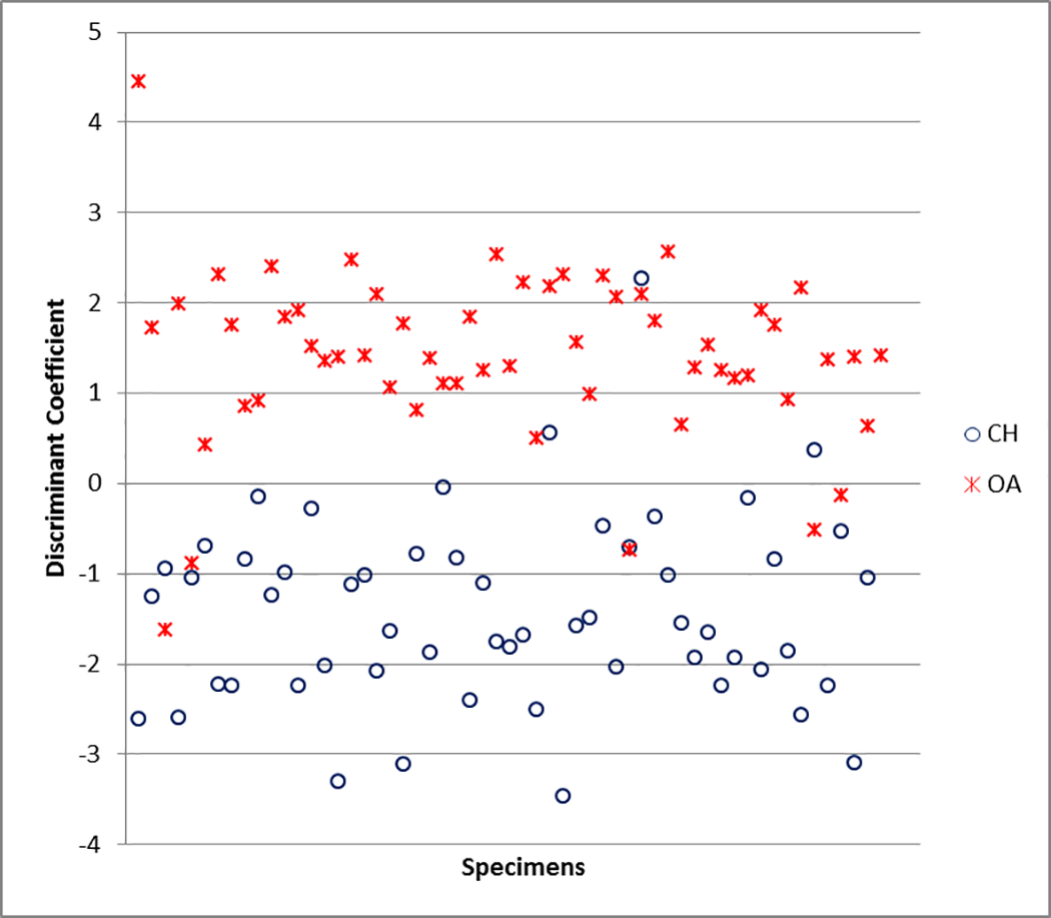
ULNA: Scatterplot of the individual discriminant scores. CH = *Capra hircus*; OA = *Ovis aries*.

The ulna has provided some of the best results. The measurements DPA and SDO along with BPC (S6 Table) are the most important, contributing heavily to the separation. The classification rate for this element is 92.9% (S5 Table). When the Individual Discriminant Coefficients are plotted (Fig 43), despite a few specimens falling in the ‘wrong’ area, the occurrence of two distinct groups is evident.

**Fig 44:**
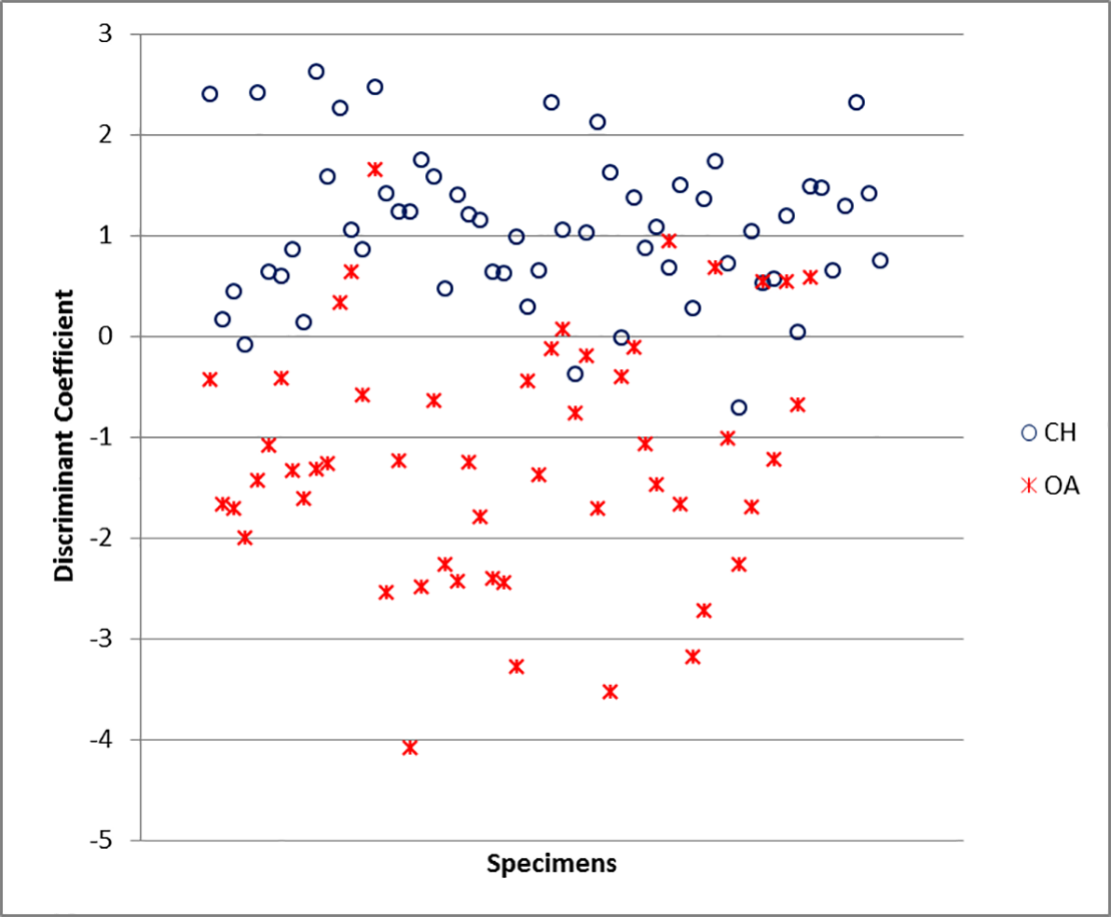
TIBIA: Scatterplot of the individual discriminant scores. CH = *Capra hircus*; OA = *Ovis aries*.

For the tibia, Dda is clearly the most important variable contributing to the separation (S6 Table). The re-classification result is 89.1% (S5 Table), a successful percentage when one considers that this element has scarcely been considered in previous studies ([28]; [15]; [4]).The scatterplot of the Individual Discriminant Coefficients (Fig 44) shows that, despite some overlap, most specimens fall into different areas of the graph.

**Fig 45:**
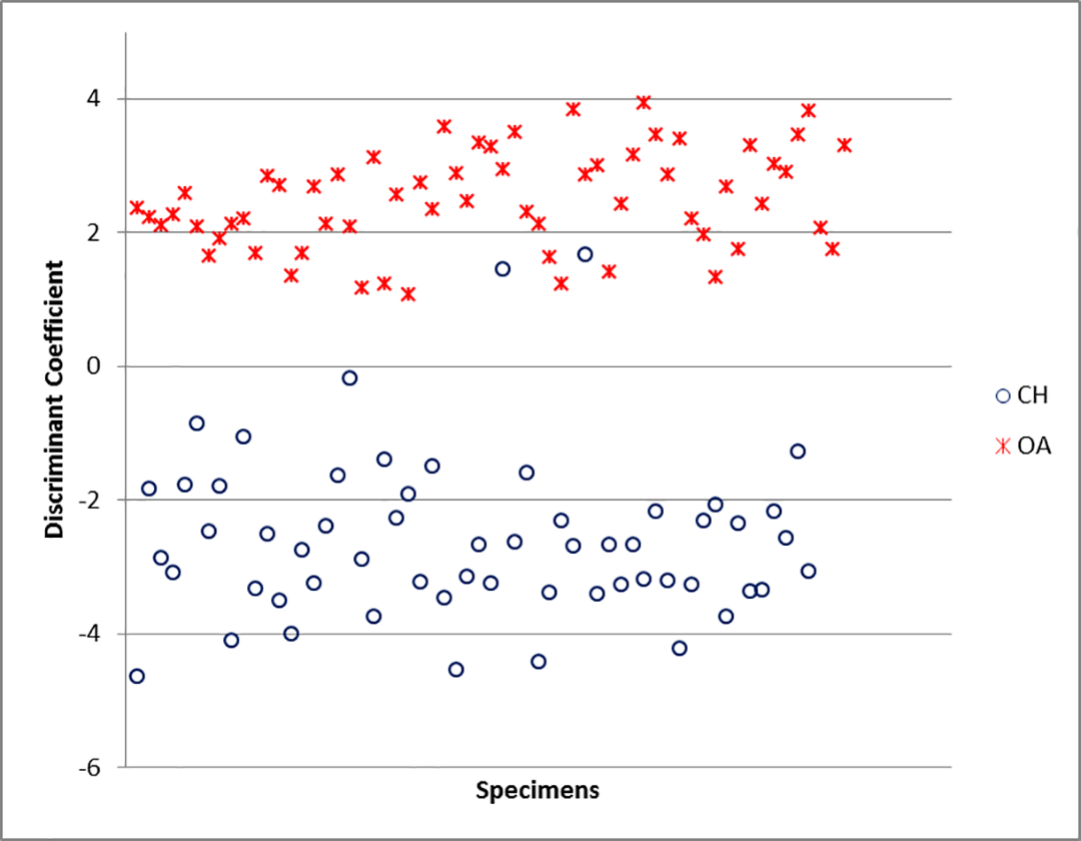
METACARPAL: Scatterplot of the individual discriminant scores. CH = *Capra hircus*; OA = *Ovis aries*.

On the basis of previous work, expectations were high for the taxonomic distinctiveness of the metacarpal, and these found confirmation in the DA analysis. GL and 1 along with BFd, a, 5, b and 2 contribute highly to the discrimination (S6 Table). The overall percentage of grouped cases correctly classified is 98.3% (S5 Table), a score which leaves a very low probability of erroneous attributions. Fig 45 displays two clearly distinct groups with just two goat specimens plotting in the sheep area.

**Fig 46:**
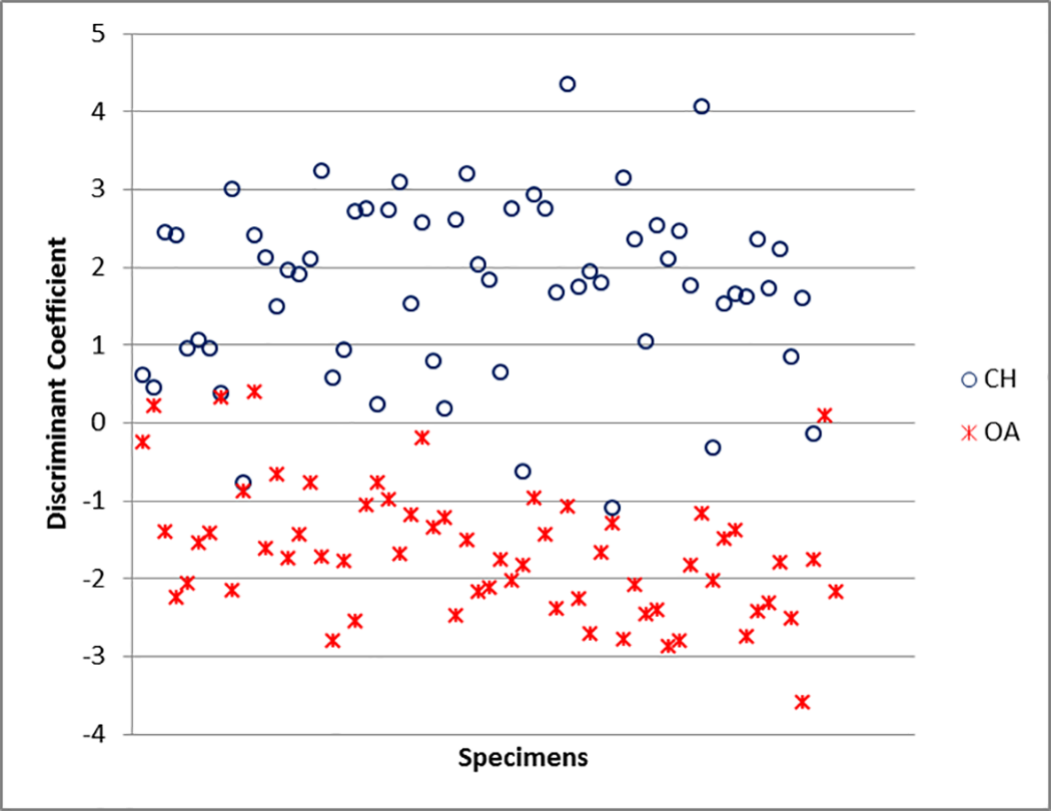
METATARSAL: Scatterplot of the individual discriminant scores. CH = *Capra hircus*; OA = *Ovis aries*.

Although not as clear-cut as for the metacarpal, the metatarsal also produced encouraging results. For this element GL, 5, 6 and 3 play a major role in discriminating between the two groups (S6 Table). The percentage of correct attributions for the metatarsal is 92.7% (S5 Table). When the Individual Discriminant Scores are plotted (Fig 46) the presence of two almost completely distinct groups is clearly visible; the separation between them is not as clear as for metacarpals, but the overlap is not particularly significant, attesting to the diagnostic potential of this element.

**Fig 47:**
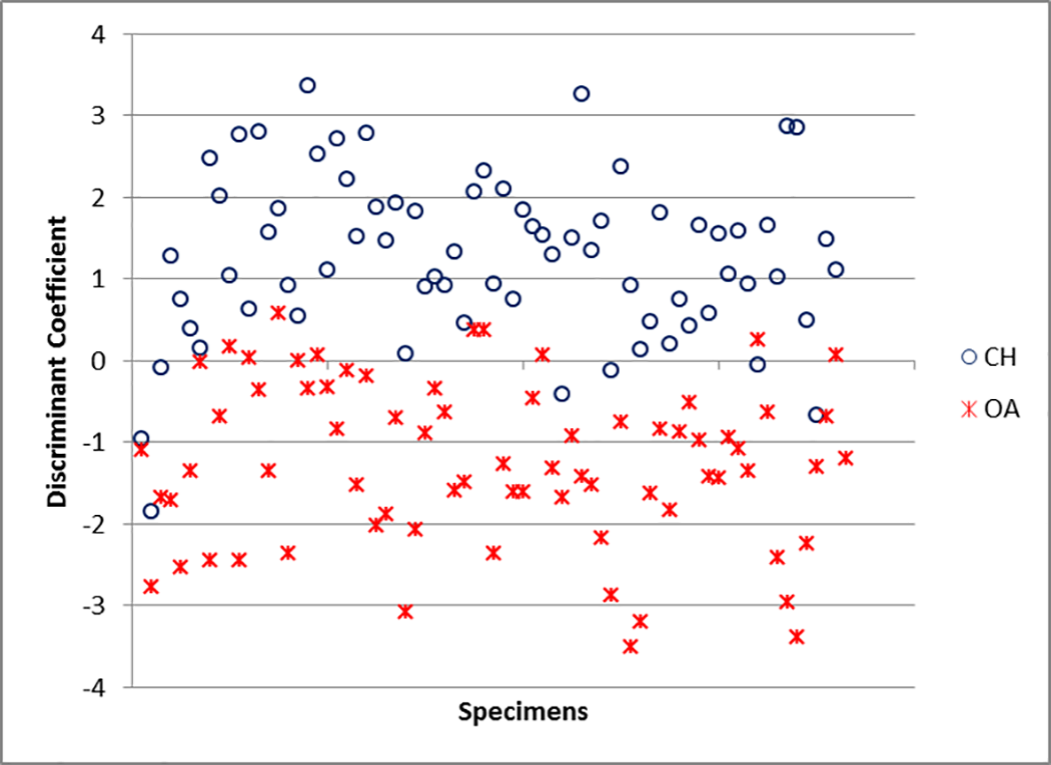
ASTRAGALUS: Scatterplot of the individual discriminant scores. CH = *Capra hircus*; OA = *Ovis aries*.

Of the measurements taken on the astragalus, H and GLl are the most important, along with Dl and Bd; these have a major impact on the discriminating power of the function (S6 Table). The correct attribution score is of 89.0% (S5 Table), a high percentage, although not the highest found so far. If the scatterplot of the Individual Discriminant Coefficients is considered (Fig 47), the distribution of the specimens attests that, despite some overlap, the astragalus is probably a useful bone to separate the two species.

**Fig 48:**
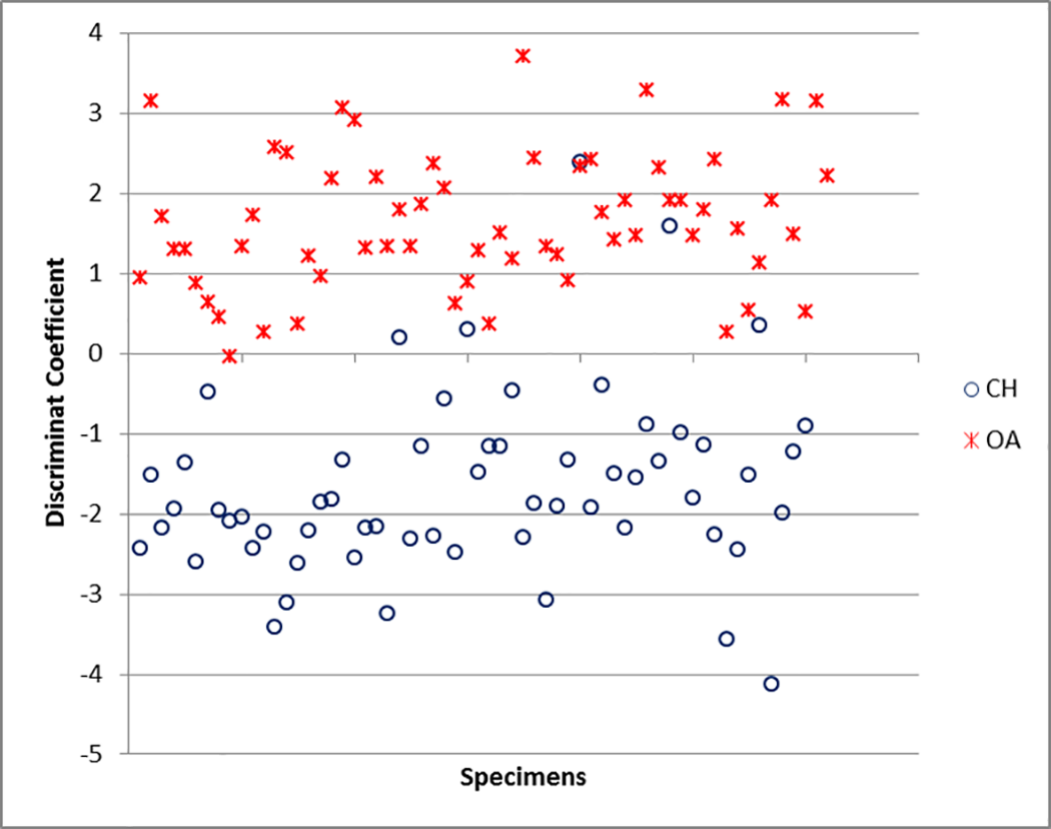
CALCANEUM: Scatterplot of the individual discriminant scores. CH = *Capra hircus*; OA = *Ovis aries*.

The importance of c and GL for the calcaneum is confirmed by S6 Table. The correct attribution rate for this element is very high at 95.1% (S5 Table). The scatterplot of the Individual Discriminant Scores (Fig 48) shows a good separation between the two groups with just a few goat specimens falling in the sheep area of the graph.

Discriminant Analysis was also run on the 3^rd^ phalanx, but the results revealed that the measurements for this element were affected by multicollinearity and, as such, the results cannot be considered to be reliable ([31]: 223).

## 1. Discussion and conclusions

The analysis of biometrical indices on modern sheep and goat collections has demonstrated the excellent potential of such approach in separating these two closely related species. Table 2 summarizes the most effective biometrical indices for each bone. Their purpose is to translate morphological differences between the two species into biometrical attributes.

**Table 2:** List of the most useful combinations of measurements for the separation of sheep and goat bones. * indicates the uncertainty of the discriminant power of the measurements as affected by multicollinearity.

Anatomical elements	Biometrical Indices	Discriminant Analysis
Horncore	E/F versus A/F	D and E
Scapula	ASG/SLC versus GLP/BG; GLP/LG versus GLP/BG;	ASG, GLP and to a lesser degree LG
Humerus	BE/HTC versus BE/BT; BEI/BT versus BEI/Bd	BE, BEI and to a lesser degree HTC
Radius	BFp/Bp versus Dp	Bp and GL
Ulna	BPC/DPA versus BPC/SDO	DPA, BPC and to a lesser degree SDO
Tibia	Bd versus Dda/Ddb	Dda, GL and to a lesser extent SD
Metacarpal	1/a versus 1/2; 4/b versus 4/5; BFd/GL versus SD/GL	a, b, 5, BFd and GL
Metatarsal	1/a versus 1/2; 4/b versus 4/5; BFd/GL versus SD/GL	b, 3, 5, 6, BFd, GL
Astragalus	H/Dl versus Bd/GLl; Bd/Dl versus Dl/GLl; Bd/H versus Bd/GLl	H, Dl, GLl and Bd
Calcaneum	c/B versus c/d; DS/c versus c/d; DS/c versus c/B	c and GL
3^rd^ Phalanx	DLS versus MBS/DLS	DLS and MBS*

The efficacy of the proposed measurements is confirmed and supported by the use of statistical tools. When DA was applied to evaluate whether the simultaneous use of several measurements provided a better separation between the two species, the results were generally consistent with those of the biometrical indices. Those measurements that had proven to be more successful in the use of biometrical indices were generally most significant in determining the discriminant power of a function (Table 2). Remarkably, no element provided an identification score lower than 83%, which means that, even in the worst case scenario, less than 17% of the specimens risked attribution to the wrong species. However, to misidentify one or two out of ten specimens in an archaeological assemblage would still, for many zooarchaeologists, represent an unacceptable degree of error. This is why this morphometric analysis needs to rely on multiple lines of evidence and must be used in combination with the more traditional morphological observations, rather than in place of them. It is also important to consider that the analysis presented above assumes that all specimens will be attributed to ‘sheep’ or ‘goat’, but zooarchaeologists are well used to also including a ‘sheep/goat’ category or ‘pseudo-taxon’ whenever specimens present ambiguous morphological characteristics and/or when the specimen is too damaged to provide useful distinguishing traits. Thus, using a cautious approach and a diversity of criteria and analytical approaches, it will be possible to further reduce the possibility of error to negligible levels.

The proposed methods and data can easily be used for the interpretation of archaeological specimens, whether they are fragmented or not (see [19]). The computer programme SPSS (or any other statistical package) attributes an individual score to each of the sheep/goat archaeological cases. This score represents the distance of that new specimen from the group centroid value calculated for each modern group (i.e. group means of the predictor variables; [31]: 620). As a consequence, the program itself reattributes to species level (prediction) the archaeological specimens on the basis of their individual scores; the group (i.e. sheep or goat) to which the new cases will be attributed is the one from which their distance is smallest [35].

The morphometric approach, particularly with the support of DA, represents an important step towards solving the sheep and goat identification issue in zooarchaeology. It can be used to validate or reject morphological identifications and, in some cases, can also help the specific identification of specimens morphologically attributed to the generic *Ovis/Capra* category. As mentioned above, however, it is important to suggest positive identifications only in those cases in which there is consistency between several lines of evidence. Thus a reattribution of a specimen initially left uncertain as ‘sheep/goat’ should only be proposed when the biometrical indices and the DA provide clear and consistent results. An example of how to apply the new methodology on archaeological material and its potential is briefly presented in Figs A-B and Table A in S2 File. The workflow used in the case presented consisted of:

- a first initial morphological identification, which was carried out along with the measuring of the bone during the recording phase;
- successively, biometrical indices were created for the archaeological material (and plotted alongside the modern sample data) in order to see if there was consistency between morphological and biometrical results;
- finally, DA was conducted with the inclusion of all measurements at once to classify the archaeological specimens. The results were then compared once again with both previous approaches to further check consistency between outcomes.

It is important to emphasise that, although this new approach can substantially enhance our ability to discriminate between postcranial bones of sheep and goats from archaeological sites, its main aim is to make identifications more objective. The publication of morphometric diagrams should become routine practice in zooarchaeological reports as this provides the opportunity for the proposed identifications to be scrutinised, thus avoiding the problem of highly questionable identification that has hitherto affected the literature. Even when only small datasets are available (e.g. n<10) this can provide significant results when compared with the baseline of modern measurements (S2 File), against which archaeological specimens can be plotted.

Regular applications of this approach to archaeological assemblages will also provide the opportunity to refine the methods, by incorporating new variables that can affect different caprine populations from different areas and periods. It is indeed well-known that identification criteria may be variably applicable to different populations and geographic types. Since the modern dataset used focuses on European breeds, the new methodology may have some limitations when applied to non-European animals. Satisfactory results were obtained when the method was applied to English medieval sheep and goat assemblages (S2 File), but applications to other geographic and historical contexts will be valuable and can contribute to refine the method and make it more globally applicable. The morphometric method should mark a new era in tackling the old issue of distinguishing between sheep and goat, providing much needed objectivity to the problem and developing a direction previously indicated only by a handful of other researchers ([10]; [11]; [12]; [13]; [14]). In the future it should also be possible to develop morphometry to a new level, for instance by adopting a geometric morphometric approach [36]. It is, however, important to understand firstly the full potential of linear measurements, also in order to provide a tool that is inclusive and feasible even when budget and time are severely limited, a common occurrence in today’s academia and commercial archaeology.

Although the distinction between sheep and goat may appear as a purely technical concern in archaeology, it is important to emphasise that these species have played a fundamental role in the history of human societies. Our inability to satisfactorily discriminate between them has detrimentally affected our understanding of human cultural and economic evolution and therefore an investment in improving methods to distinguish between these two species is a priority. In this paper we hope to have provided a substantial contribution to this quest, in particular recommending a method that will allow a more objective presentation and evaluation of the results.

## Supporting information

S1 Table Goat and sheep specimens included in the sample studied. (DOCX)

S2 Table List of measurements used for this study. (DOCX)

S3 Table Median, effect size, Mann-Whitney U test and Bonferroni adjustment results. (DOCX)

S4 Table Results from Manova for each combination of ratios used in the allometric shape analysis. (DOCX)

S5 Table Percentage of correct classifications by element and species from linear discriminant analysis. (DOCX)

S6 Table Structure matrix table with the correlation coefficients for each element and each variable/measurement for discriminant analysis. (DOCX)

S1 File Inter and intra observer error results. (DOCX)

S2 File Example of how to apply the new methodology on archaeological material: Sheep and goat scapulae found at the medieval site of Woolmonger/Kingswell street in Northampton (bones from phase 2, i.e. c. AD 1100–1400). (DOCX)

S1 Dataset Biometrical raw data of the modern material. (ACCDB)
