# Supplementary material for: A morphometric system to distinguish sheep and goat postcranial bones
Source: PLoS One. 2017 Jun 8;12(6):e0178543. doi: 10.1371/journal.pone.0178543 (PMC5464554; doi:10.1371/journal.pone.0178543)
Supplement: S1 Table — (DOCX) [file pone.0178543.s001.docx]

**S1A Table. Goat specimens included in the sample studied. The information given in this table (breed, sex and age) is as provided by the collection data-bases consulted.**

| **SPECIMENS FROM THE ZOOARCHAEOLOGY LABORATORY, UNIVERSITY OF SHEFFIELD, UK** | | | | | | | | |
| --- | --- | --- | --- | --- | --- | --- | --- | --- |
| **Species** | **ID Number** | **Location** | **Origin** | **Breed** | **Sex** | **Skeleton** | | **Age** |
| *Capra hircus* | 90 | Sheffield University | Halkidiki, Macedonia, Greece | - | ♀ | Almost Complete | | 11 years |
| *Capra hircus* | 91 | Sheffield University | Macedonia, Greece | - | ♂ | Complete | | 7 years |
| *Capra hircus* | 94 | Sheffield University | Halkidiki, Macedonia, Greece | - | ♀ | Complete | | - |
| *Capra hircus* | 502 | Sheffield University | Katerini, Greece | - | - | Almost Complete | | - |
| *Capra hircus* | 762 | Sheffield University | Assiros, Greece | - | - | Almost Complete | | - |
| *Capra hircus* | 784 | Sheffield University | Assiros,  Greece | - | ♀ | Almost Complete | | - |
| *Capra hircus* | 790 | Sheffield University | Assiros, Greece | - | - | Almost Complete | | - |
| *Capra hircus* | 808 | Sheffield University | Kartere, Greece | - | ♂ | Complete | | - |
| *Capra hircus* | 1053 | Sheffield University | Mystras, Greece | - | - | Almost Complete | | - |
| *Capra hircus* | 1581 | Sheffield University | Tony Legge Collection* | - | - | Almost Complete | | - |
| **TOTAL NUMBER 10** | | | | | | | | |
| **SPECIMENS FROM THE COLLECTION HOSTED AT HISTORIC ENGLAND, FORT CUMBERLAND, PORTSMOUTH, UK** | | | | | | | | |
| *Capra hircus* | 45dg | English Heritage,  Portsmouth | Scotland | - | - | Complete | | - |
| *Capra hircus* | 1315 | English Heritage,  Portsmouth | - | Toggenburg | ♂ | Almost Complete | | 3.5 years |
| *Capra hircus* | 1631 | English Heritage,  Portsmouth | Cyprus | Damascus | ♀ | Almost Complete | | 7 months |
| *Capra hircus* | 2199 | English Heritage,  Portsmouth | England | Old English | ♂ | Almost Complete | | 15 months |
| *Capra hircus* | 2774 | English Heritage,  Portsmouth | Durham | Bagot | ♂ | Almost Complete | | 2 years 7 months |
| *Capra hircus* | 3318 | English Heritage,  Portsmouth | Islay, Hebrides | Feral | ♂ | Almost Complete | | Adult |
| *Capra hircus* | 3323 | English Heritage,  Portsmouth | Islay, Hebrides | Feral | ♂ | Complete | | Adult |
| *Capra hircus* | 501 | English Heritage,  Portsmouth | Whipsnade Zoo, Bedfordshire | White goat | ♂ | Almost Complete | | 2 years |
| *Capra hircus* | 502 | English Heritage,  Portsmouth | - | White goat | ♂ | Complete | | Unknown |
| **TOTAL NUMBER 9** | | | | | | | | |
| **SPECIMENS FROM THE ZOOARCHAEOLOGY LABORATORY, UNIVERSITY OF YORK, UK** | | | | | | | | |
| *Capra hircus* | 511 | York University | - | Saanen | ♀ | | Almost Complete | 2 years |
| *Capra hircus* | 512 | York University | - | Saanen Anglo-Nubian | ♂ | | Almost Complete | 7 months |
| *Capra hircus* | 515 | York University | - | Unknown | ♀ | | Complete | 4 years |
| *Capra hircus* | 544 | York University | - | Saanen | ♀ | | Incomplete | Adult |
| *Capra hircus* | 700 | York University | - | - | ♂ | | Almost Complete | Adult |
| **TOTAL NUMBER 5** | | | | | | | | |
| **SPECIMENS FROM THE NATIONL MUSEUM OF CARDIFF (NODDLE COLLECTION) WALES, UK** | | | | | | | | |
| *Capra hircus* | 112004011 | National Museum Cardiff | Noddle Collection | Feral Rhum | - | Almost Complete | | - |
| *Capra hircus* | 112004012 | National Museum Cardiff | Noddle Collection | Feral Rhum | - | Almost Complete | | - |
| *Capra hircus* | 112004016 | National Museum Cardiff | Noddle Collection | - | - | Almost Complete | | - |
| *Capra hircus* | 112004019 | National Museum Cardiff | Noddle Collection | - | - | Almost Complete | | - |
| *Capra hircus* | 112004020 | National Museum Cardiff | Noddle Collection | Feral | - | Almost Complete | | - |
| *Capra hircus* | 112004021 | National Museum Cardiff | Noddle Collection | Feral | - | Almost Complete | | - |
| *Capra hircus* | 112004022 | National Museum Cardiff | Noddle Collection | Feral | - | Almost Complete | | - |
| *Capra hircus* | 112004032 | National Museum Cardiff | Noddle Collection | Welsh goat | ♀ | Almost Complete | | 8 months |
| *Capra hircus* | 112004033 | National Museum Cardiff | Noddle Collection | Feral Rhum | - | Almost Complete | | - |
| *Capra hircus* | 112004034 | National Museum Cardiff | Noddle Collection | Feral Rhum | - | Almost Complete | | - |
| *Capra hircus* | 112004035 | National Museum Cardiff | Noddle Collection | Feral Galloway | - | Almost Complete | | - |
| *Capra hircus* | 112004036 | National Museum Cardiff | Noddle Collection | Golden Guernsey | - | Almost Complete | | - |
| *Capra hircus* | 112004040 | National Museum Cardiff | Noddle Collection | Northumberland goat | - | Almost Complete | | - |
| *Capra hircus* | 1120040401 | National Museum Cardiff | Noddle Collection | Northumberland goat | - | Almost Complete | | - |
| *Capra hircus* | 112004080 | National Museum Cardiff | Noddle Collection | - | - | Almost Complete | | - |
| *Capra hircus* | 112004081 | National Museum Cardiff | Noddle Collection | - | - | Complete | | - |
| **TOTAL NUMBER 16** | | | | | | | | |
| **SPECIMENS FROM THE JULIUS KÜHN MUSEUM, HALLE, GERMANY** | | | | | | | | |
| *Capra hircus* | C igz 3 (82) | Julius Kühn Museum,  Halle | - | Langensalzaer | ♀ | Complete | | Adult |
| *Capra hircus* | C igz 1 (83) | Julius Kühn Museum,  Halle | - | Langensalzaer | ♂ | Complete | | Adult |
| *Capra hircus* | C swd 2 | Julius Kühn Museum,  Halle | - | Schwarzwalder | ♀ | Complete | | 8 years 11 months |
| *Capra hircus* | C frb 1 | Julius Kühn Museum,  Halle | - | Freiburger | ♀ | Complete | | Adult |
| *Capra hircus* | C 3 | Julius Kühn Museum,  Halle | - | Weiße | ♀ | Complete | | Adult |
| *Capra hircus* | C bdn 2 | Julius Kühn Museum,  Halle | - | Bundener | ♀ | Almost Complete | | Adult |
| *Capra hircus* | C bdn 3 | Julius Kühn Museum,  Halle | - | Bundener | ♀ | Complete | | Adult |
| *Capra hircus* | C bdn 4 | Julius Kühn Museum,  Halle | - | Bundener | ♀ | Complete | | (bought 25/10/1886 dead 06/05/1888 |
| *Capra hircus* | C19 | Julius Kühn Museum,  Halle | - | - | ♀ | Complete | | - |
| *Capra hircus* | Cd 1 | Julius Kühn Museum,  Halle | - | - | Hermaphrodite | Complete | | Adult |
| *Capra hircus* | C saa 3 | Julius Kühn Museum,  Halle | - | Saanen | ♀ | Complete | | 3 years 2 months |
| *Capra hircus* | C ggb 1 | Julius Kühn Museum,  Halle | - | Guggisberger | ♂ | Complete | | 2 years 7 months |
| *Capra hircus* | C appz 1 | Julius Kühn Museum,  Halle | - | Appenzeller | ♀ | Almost Complete | | Adult |
| *Capra hircus* | C saa 6 | Julius Kühn Museum,  Halle | - | Saanen | ♀ | Complete | | Adult |
| *Capra hircus* | C saa 7 | Julius Kühn Museum,  Halle | - | Saanen | ♀ | Complete | | 2 years 1 month |
| *Capra hircus* | C saa 1 | Julius Kühn Museum,  Halle | - | Saanen | ♂ | Complete | | 3 years 5 months |
| *Capra hircus* | C wal 6 | Julius Kühn Museum,  Halle | - | Walliser | ♂ | Almost Complete | | 4 years 2 months |
| *Capra hircus* | C wal 8 | Julius Kühn Museum,  Halle | - | Walliser | ♀ | Almost Complete | | 2 years 9 months |
| *Capra hircus* | C saa 2 | Julius Kühn Museum,  Halle | - | Saanen | ♀ | Complete | | 3 years 5 months |
| *Capra hircus* | C wal 7 | Julius Kühn Museum,  Halle | - | Walliser | ♂ | Incomplete | | 2 years 6 months |
| *Capra hircus* | C blk 2 | Julius Kühn Museum,  Halle | - | Balkan | ♀ | Complete | | (bought 1916-dead 1917) |
| **TOTAL NUMBER 21** | | | | | | | | |
| **SPECIMENS FROM THE ZOOLOGICAL MUSEUM, UNIVERSITY OF KIEL, GERMANY** | | | | | | | | |
| *Capra hircus* | 1912 | Zoologisches museum Kiel | - | Zwerg | ♀ | | Almost Complete | Adult |
| *Capra hircus* | 7176 | Zoologisches museum Kiel | - | Ziegenbock | ♂ | | Complete | Adult |
| *Capra hircus* | 7535 | Zoologisches museum Kiel | - | Saanan | ♂ | | Almost Complete | Adult |
| *Capra hircus* | 18719 | Zoologisches museum Kiel | - | Weiße Deutsche Edelziege | ♀ | | Almost Complete | Adult |
| *Capra hircus* | 19506 | Zoologisches museum Kiel | - | Damara | ♀ | | Complete | Adult |
| *Capra hircus* | 22221 | Zoologisches museum Kiel | - | - | - | | Almost Complete | Adult |
| *Capra hircus* | 22222 | Zoologisches museum Kiel | - | - | ♂ | | Incomplete | Adult |
| *Capra hircus* | 30447 | Zoologisches museum Kiel | - | Walliser Schwarzhals | ♀ | | Almost Complete | 11 years |
| *Capra hircus* | 33040 | Zoologisches museum Kiel | - | Weiße Deutsche Edelziege | ♂ | | Almost Complete | Adult |
| **TOTAL NUMBER 9** | | | | | | | | |
| **SPECIMENS FROM THE NATURAL HISTORY MUSEUM, BERLIN, GERMANY** | | | | | | | | |
| *Capra hircus* | 100 | Naturkunde museum, Berlin | - | Bezoar | ♂ | | Almost Complete | - |
| *Capra hircus* | 1556 | Naturkunde museum, Berlin | - | - | ♀ | | Almost Complete | 8 years |
| *Capra hircus* | 1854 | Naturkunde museum, Berlin | - | Angora | ♀ | | Almost Complete | - |
| *Capra hircus* | 3638 | Naturkunde museum, Berlin | - | - | ♂ | | Almost Complete | - |
| *Capra hircus* | 4487 | Naturkunde museum, Berlin | - | Beden | ♀ | | Almost Complete | - |
| *Capra hircus* | 6945 | Naturkunde museum, Berlin | - | Mamber | ♀ | | Almost Complete | - |
| *Capra hircus* | 6998 | Naturkunde museum, Berlin | - | Sardinische Heidschnucke | ♂ | | Complete | - |
| *Capra hircus* | 7555 | Naturkunde museum, Berlin | - | - | - | | Complete | - |
| *Capra hircus* | 94892 | Naturkunde museum, Berlin | - | - | - | | Incomplete | ¾ of a year |
| **TOTAL NUMBER 9** | | | | | | | | |
| **TOTAL NUMBER OF GOAT** | | | | **79** | | | | |

**S1B Table. Sheep specimens included in the sample studied. The information given in this table (breed, sex and age) is as provided by the collection data-bases consulted.**

| **SPECIMENS FROM THE ZOOARCHAEOLOGY LABORATORY, UNIVERSITY OF SHEFFIELD, UK** | | | | | | |
| --- | --- | --- | --- | --- | --- | --- |
| **Species** | **Location** | **Origin** | **Breed** | **Sex** | **Skeleton** | **Age** |
| *Ovis aries* | Sheffield University | - | - | - | Almost Complete | Sub-adult |
| *Ovis aries* | Sheffield University | Sheffield | Blackface | - | Almost Complete | - |
| *Ovis aries* | Sheffield University | Oaker farm | - | ♀ | Complete | Adult |
| *Ovis aries* | Sheffield University | Peak District, Derbyshire | - | - | Almost Complete | Adult |
| *Ovis aries* | Sheffield University | - | - | - | Almost Complete | - |
| *Ovis aries* | Sheffield University | - | - | - | Almost Complete | Adult |
| *Ovis aries* | Sheffield University | Sheffield | - | - | Almost Complete | Sub-adult |
| *Ovis aries* | Sheffield University | Flag Fen, Peterborough, Cambridgeshire | Soay | ♂ | Complete | Elderly |
| *Ovis aries* | Sheffield University | Flag Fen, Peterborough, Cambridgeshire | Soay | ♀ | Complete | Adult |
| *Ovis aries* | Sheffield University | Graves Park rare Breeds Centre, Sheffield | White-faced woodland | ♀ | Almost Complete | 6-7 years |
| *Ovis aries* | Sheffield University | Graves Park, Sheffield | Portland | ♀ | Complete | 5 years (born 1989) |
| *Ovis aries* | Sheffield University | - | - | - | Almost Complete | Adult |
| *Ovis aries* | Sheffield University | Flag Fen, Peterborough, Cambridgeshire | Soay | ♀ | Complete | More than 8 years |
| *Ovis aries* | Sheffield University | Flag Fen, Peterborough, Cambridgeshire | Soay | ♀ | Complete | More than 8 years |
| *Ovis aries* | Sheffield University | Langdale, Lake District | Herdwick | - | Almost Complete | - |
| *Ovis orientalis* | Sheffield University | Flag Fen, Peterborough, Cambridgeshire | - | ♀ | Almost Complete | Adult |
| *Ovis aries* | Sheffield University | 2km outside Krithia, on road to Assiros | - | - | Complete | - |
| *Ovis aries* | Sheffield University | Biggin Dale, Hartington, Derbyshire | - | - | Almost Complete | - |
| *Ovis aries* | Sheffield University | Quarry near Korinos, Katerini, Greece | - | - | Almost Complete | - |
| *Ovis aries* | Sheffield University | Quarry near Korinis, Katerini, Greece | - | - | Almost Complete | - |
| *Ovis aries* | Sheffield University | Quarry near Korinis, Katerini, Greece | - | ♀ | Almost Complete | - |
| *Ovis aries* | Sheffield University | Picos de Europa, Spain | - | - | Incomplete | - |
| *Ovis aries* | Sheffield University | Merv, Turkmenistan | Afghan Arabi? (local name) | - | Almost Complete | - |
| *Ovis aries* | Sheffield University | Beeley Moor, Chatsworth, Derbyshire | - | - | Almost Complete | Adult |
| *Ovis aries* | Sheffield University | Langdale, Lake District | Herdwick | - | Complete | - |
| *Ovis aries* | Sheffield University | Assiros, Greece | - | - | Almost Complete | Juvenile |
| **TOTAL NUMBER 26** | | | | | | |
| **SPECIMENS FROM THE COLLECTION HOSTED AT HISTORIC ENGLAND, FORT CUMBERLAND, PORTSMOUTH, UK** | | | | | | |
| *Ovis aries* | Historic England,  Portsmouth | - | Soay | ♀ | Complete | 12 years |
| *Ovis aries* | Historic England,  Portsmouth | - | Soay | ♀ | Almost Complete | 21-25 months |
| *Ovis aries* | Historic England, Portsmouth | - | Soay | ♀ | Complete | 10 years |
| *Ovis aries* | Historic England,  Portsmouth | ex. Woburn | Soay | ♀ | Complete | 54 months |
| *Ovis aries* | Historic England, Portsmouth | Hirta, St. Kilda | Soay | ♂ | Complete | Adult |
| *Ovis aries* | Historic England,  Portsmouth | Hoy, Orkney | Shetland | ♀ | Complete | 4 years and 7 months |
| *Ovis aries* | Historic England, Portsmouth | Hoy, Orkney | Shetland | ♀ | Complete | 79 months |
| *Ovis aries* | Historic England,  Portsmouth | Hoy, Orkney | Shetland | ♀ | Complete | 4 years and 7 months |
| *Ovis aries* | Historic England, Portsmouth | Hoy, Orkney | Shetland | ♀ | Complete | 6 years and 7 months |
| *Ovis aries* | Historic England, Portsmouth | Hoy, Orkney | Shetland | ♀ | Complete | 67 months |
| *Ovis aries* | Historic England, Portsmouth | - | Soay | ♂? | Almost Complete | - |
| *Ovis aries* | Historic England, Portsmouth | Hoy, Orkney | Shetland | Castrated | Almost Complete | 24 months |
| *Ovis aries* | Historic England, Portsmouth | Hoy, Orkney | Shetland | Castrated | Almost Complete | 39 months |
| *Ovis aries* | Historic England, Portsmouth | Hoy, Orkney | Shetland | Castrated | Almost Complete | 27 months |
| *Ovis aries* | Historic England, Portsmouth | Hoy, Orkney | Shetland | Castrated | Almost Complete | 30.5 months |
| *Ovis aries* | Historic England, Portsmouth | Hoy, Orkney | Shetland | Castrated | Almost Complete | 52.5 months |
| *Ovis aries* | Historic England, Portsmouth | Hoy, Orkney | Shetland | Castrated | Almost Complete | 45 months |
| *Ovis aries* | Historic England, Portsmouth | Hoy, Orkney | Shetland | Castrated | Almost Complete | 52.5 months |
| *Ovis aries* | Historic England, Portsmouth | Hoy, Orkney | Shetland | ♂ | Almost Complete | 22 months |
| *Ovis aries* | Historic England, Portsmouth | Hoy, Orkney | Shetland | ♂ | Almost Complete | 28 months |
| *Ovis aries* | Historic England, Portsmouth | Hoy, Orkney | Shetland | ♂ | Almost Complete | 24 months |
| *Ovis aries* | Historic England, Portsmouth | - | Soay | ♀ | Almost Complete | 42 months |
| *Ovis aries* | Historic England, Portsmouth | - | Soay | ♀ | Complete | 41 months |
| *Ovis aries* | Historic England, Portsmouth | - | Soay | ♀ | Complete | 41 months |
| *Ovis aries* | Historic England, Portsmouth | Hoy, Orkney | Shetland | ♂ | Complete | 23 months |
| *Ovis aries* | Historic England, Portsmouth | Cambridgeshire | Shetland | ♀ | Complete | 6.75 years |
| *Ovis aries* | Historic England, Portsmouth | - | Soay | ♀ | Complete | 45 months |
| *Ovis aries* | Historic England, Portsmouth | Durham | Soay | Castrated | Complete | 35 months |
| *Ovis aries* | Historic England, Portsmouth | Suffolk | Soay | ♀ | Complete | 10 years |
| *Ovis aries* | Historic England, Portsmouth | Cambridgeshire | Soay | Castrated | Complete | 13 years |
| *Ovis aries* | Historic England, Portsmouth | - | Shetland | Castrated | Almost Complete | 2 years and 8 months |
| *Ovis aries* | Historic England, Portsmouth | - | Shetland | Castrated | Almost Complete | 20 months |
| *Ovis aries* | Historic England, Portsmouth | - | Shetland | Castrated | Almost Complete | 18 months |
| *Ovis aries* | Historic England, Portsmouth | - | Shetland | Castrated | Almost Complete | 3 years and 7 months |
| *Ovis aries* | Historic England, Portsmouth | - | Shetland | Castrated | Almost Complete | 2 years and 7 months |
| *Ovis aries* | Historic England, Portsmouth | Hoy, Orkney | Shetland | Castrated | Almost Complete | 45 months |
| *Ovis aries* | Historic England, Portsmouth | Hoy, Orkney | Shetland | Castrated | Almost Complete | c. 941 days |
| *Ovis aries* | Historic England, Portsmouth | Hoy, Orkney | Shetland | Castrated | Almost Complete | 941 days |
| *Ovis aries* | Historic England, Portsmouth | St Kilda | Soay | ♂ | Complete | 31 months |
| *Ovis aries* | Historic England, Portsmouth | Hoy, Orkney | Shetland | ♂ | Complete | 2 years and 7 months |
| *Ovis aries* | Historic England, Portsmouth | Hoy, Orkney | Shetland | ♂ | Complete | 2 years and 7 months |
| *Ovis aries* | Historic England, Portsmouth | Hoy, Orkney | Shetland | ♂ | Complete | 31 months |
| *Ovis aries* | Historic England, Portsmouth | Hoy, Orkney | Shetland | ♂ | Complete | 2 years and 7 months |
| *Ovis aries* | Historic England, Portsmouth | Hoy, Orkney | Shetland | ♂ | Complete | 2 years and 7 months |
| *Ovis aries* | Historic England, Portsmouth | Butser Iron Age Farm | Soay | ♀ | Complete | Adult |
| **TOTAL NUMBER 45** | | | | | | |
| **SPECIMENS FROM THE ZOOLOGICAL MUSEUM, UNIVERSITY OF KIEL, GERMANY** | | | | | | |
| *Ovis aries* | Zoologisches museum Kiel | - | Heidschnucke | ♀ | Almost Complete | **-** |
| *Ovis aries* | Zoologisches museum Kiel | - | Ostfriesisches Milch | **♀** | Complete | **-** |
| *Ovis aries* | Zoologisches museum Kiel | - | Blu Domane | **♀** | Complete | **-** |
| *Ovis aries* | Zoologisches museum Kiel | - | Heidschnucke Romanow | **♂** | Almost Complete | 14/16 months |
| *Ovis aries* | Zoologisches museum Kiel | - | Heidschnucke | **♂** | Almost Complete | - |
| *Ovis aries* | Zoologisches museum Kiel | - | Deutsches Weißköpfiges Fleischschaf | ♀ | Complete | 2 years |
| *Ovis aries* | Zoologisches museum Kiel | - | Rotkopf | ♀ | Complete | - |
| **TOTAL NUMBER 7** | | | | | | |
| **TOTAL NUMBER OF SHEEP** | | | **78** | | | |
