# Supplementary material for: A morphometric system to distinguish sheep and goat postcranial bones
Source: PLoS One. 2017 Jun 8;12(6):e0178543. doi: 10.1371/journal.pone.0178543 (PMC5464554; doi:10.1371/journal.pone.0178543)
Supplement: S2 Table — (DOCX) [file pone.0178543.s002.docx]

**S2 Table. List of measurements used for this study. An asterisk near the author’s name indicates that the measurement has been slightly modified in the way it is taken; an asterisk in place of the author’s name indicates that this is a newly devised measurement by the authors.**

| **Element** | **Measurement** | **Reference** | **Description** |
| --- | --- | --- | --- |
| Horncores | A | von den Driesch 1976 | Maximum diameter of the horncore at the base |
|  | B | von den Driesch 1976 | Minimum diameter of the horncore at the base |
|  | C | * | Maximum diameter taken midway the horncore length |
|  | D | * | Minimum diameter taken midway the horncore length |
|  | E | * | Length of the horncore from the antero-medial edge of the base to the tip |
|  | F | von den Driesch 1976 | Length of the outer curvature of the horncore taken with a tape measure |
| Scapula | BG | von den Driesch 1976 | Breadth of the glenoid cavity |
|  | LG | von den Driesch 1976 | Length of the glenoid cavity |
|  | GLP | von den Driesch 1976 | Greatest length of the *processus articularis* |
|  | SLC | von den Driesch 1976 | Smallest length of the *collum scapulae* |
|  | ASG | English Heritage forthcoming  Fernandez 2001, * | Shortest distance from the base of the spine to edge of glenoid cavity |
| Humerus | BT | Von den Driesch 1976;  Payne & Bull 1988 | Breadth of the trochlea |
|  | Bd | von den Driesch 1976 | Breadth of the distal articulation |
|  | HT | Davis 1996 | Greatest height of the trochlea |
|  | HTC | Payne & Bull 1988 | Diameter of the trochlea central constriction |
|  | BE | * | Breadth of the *capitulum*, measured along the trochlear axis. |
|  | Dd | Fernandez 2001* | Depth of the distal end |
|  | BEI | * | Breadth of the epicondyle *lateralis* taken at a depth of 2-3 mm from the lateral margin |
| Radius | Bp | von den Driesch 1976 | Breadth of the proximal articulation |
|  | BFp | von den Driesch 1976 | Breadth of the *facies articularis proximalis* |
|  | Dp | Fernandez 2001; * | Depth of the proximal end |
|  | GL | von den Driesch 1976 | Greatest length |
|  | SD | von den Driesch 1976 | Smallest depth of the shaft |
| Ulna | B | Fernandez 2001; * | Breadth of the *olecranon* taken by keeping the arms of the callipers parallel to the medial face |
|  | L | Fernandez 2001; * | Length of the *olecranon* |
|  | DPA | von den Driesch 1976 | Depth across the *processus anconaeus* |
|  | BPC | von den Driesch 1976 | Greatest breadth across the coronoid process |
|  | SDO | von den Driesch 1976 | Smallest depth of the *olecranon* |
| Tibia | GL | von den Driesch 1976 | Greatest length |
|  | SD | von den Driesch 1976 | Smallest depth of the shaft |
|  | Bd | von den Driesch 1976 | Breadth of the distal articulation |
|  | Dda | von den Driesch 1976* | Depth of the distal end of the medial side |
|  | Ddb | * | Depth of the distal end of the lateral side |
| Metapodials | GL | von den Driesch 1976 | Greatest length |
|  | SD | von den Driesch 1976 | Smallest depth of the shaft |
|  | BatF | Davis 1996 | Breadth of the distal end in the point of fusion with the diaphysis |
|  | BFd | Davis 1996 | Breadth of the distal articulation |
|  | a | Payne 1969;  Davies 1996 | Medio-lateral width of the medial condyle |
|  | b | Payne 1969;  Davis 1996 | Medio-lateral width of the lateral condyle |
|  | 1 | Payne 1969*;  Davis 1996* | Diameter of the external trochlea of the medial condyle. Callipers need to be positioned at the external edge of the trochlea |
|  | 2 | Davis 1996 | Diameter of the *verticillus* on the medial condyle |
|  | 3 | Davis 1996 | Diameter of the internal trochlea of the medial condyle |
|  | 4 | Payne 1969*;  Davis 1996* | Diameter of the external trochlea of the lateral condyle. Callipers need to be positioned at the external edge of the trochlea |
|  | 5 | Davis 1996 | Diameter of the *verticillus* of the lateral condyle |
|  | 6 | Davis 1996 | Diameter of internal trochlea of the lateral condyle |
| Astragalus | Bd | von den Driesch 1976 | Breadth of the distal articulation |
|  | GLm | von den Driesch 1976 | Greatest length of the medial half |
|  | GLl | von den Driesch 1976 | Greatest length of the lateral half |
|  | Dm | von den Driesch 1976 | Greatest depth of the medial half |
|  | Dl | von den Driesch 1976 | Greatest depth of the lateral half |
|  | H | * | Height of the central constriction (i.e. minimum length) |
|  | BpT | * | Smallest breadth of the plantar trochlea |
| Calcaneum | BS | von den Driesch 1976* | Breadth taken at the height of the *substentaculum tali* |
|  | GL | von den Driesch 1976 | Greatest length |
|  | c | Fernandez 2001; * | Length of the articular facet on the calcaneum taken where the articular facet starts to project out |
|  | d | Fernandez 2001; * | Length from the articular facet to the articulation-free part of the process |
|  | B | Boessneck *et al.* 1964, 1967 | Breadth of the articular surface of the *os malleolare* |
|  | DS | English Heritage forthcoming | Greatest depth of the *substentaculum tali* |
|  | Gd | Albarella and Payne 2005 | Greatest breadth of the distal part (taken from the surface of the *os malleolare* to the plantar side in its maximum point of expansion) |
| 3^rd^ Phalanx | DLS | von den Driesch 1976 | Greatest diagonal length of the sole |
|  | MBS | von den Driesch 1976 | Middle breadth of the sole |
