# Supplementary material for: A morphometric system to distinguish sheep and goat postcranial bones
Source: PLoS One. 2017 Jun 8;12(6):e0178543. doi: 10.1371/journal.pone.0178543 (PMC5464554; doi:10.1371/journal.pone.0178543)
Supplement: S3 Table — (DOCX) [file pone.0178543.s003.docx]

S3 Table. Median, Effect Size, Mann-Whitney U test and Bonferroni adjustment results, calculated for each ratio index on each skeletal element included in the study ( *p* value significant at *p*<0.05=* and highly sgnificant at p<0.01=**).

| **Skeletal part** | **Index** | **N. Specimens** | **Median** | ***r***  **Effect size** | **Mann-Whitney U;**  ***z* approximation** | **Probability level (*p*)** | | **Bonferroni adjustment** | |
| --- | --- | --- | --- | --- | --- | --- | --- | --- | --- |
| Horncore | A:F | 35 CH  28 OA | 25.2 | -0.53 | U=182.0  ***z***= -4.261 | 0.000 | ** | 0.03 | ** |
|  | E:F |  | 88.5 | -0.65 | U= 114.0  ***z***= -5.202 | 0.000 | ** |  | ** |
| Scapula | ASG:BG | 74 CH  73 OA | 103.9 | -0.34 | U= 1609.5  ***z***= -4.229 | 0.000 | ** | 0.01 | ** |
|  | ASG:LG |  | 86.2 | -0.49 | U= 1139.0  ***z***= -6.052 | 0.000 | ** |  | ** |
|  | GLP:BG |  | 149.5 | -0.63 | U= 721.0  ***z***= -7.671 | 0.000 | ** |  | ** |
|  | GLP:LG |  | 125.9 | -0.56 | U= 1081.5  ***z***= -6.275 | 0.000 | ** |  | ** |
| Humerus | BT:HT | 76 CH  71 OA | 157.7 | -0.53 | U= 1012.5  ***z***= -6.534 | 0.000 | ** | 0.00 | ** |
|  | BT:HTC |  | 206 | -0.57 | U= 1030.5  ***z***= -6.464 | 0.000 | ** |  | ** |
|  | BE:BT |  | 31.1 | -0.37 | U= 1679.5  ***z***= -3.949 | 0.003 | ** |  | n.s. |
|  | BE:Bd |  | 29.6 | -0.30 | U= 1853.5  ***z***= -3.274 | 0.001 | ** |  | n.s. |
|  | BE:HTC |  | 63.7 | -0.54 | U= 990.5  ***z***= -6.619 | 0.000 | ** |  | ** |
|  | BEI:Bd |  | 20 | -0.65 | U= 652.0  ***z***= -7.932 | 0.000 | ** |  | ** |
|  | BEI:BT |  | 21.2 | -0.67 | U= 748.0  ***z***= -7.560 | 0.000 | ** |  | ** |
| Radius | BFp:Bp | 74 CH  71 OA | 93.3 | -0.77 | U= 266.5  ***z***= -9.337 | 0.000 | ** | 0.05 | ** |
| Ulna | BPC:DPA | 57 CH  57 OA | 79.6 | -0.76 | U= 187.5  ***z***= -8.144 | 0.000 | ** | 0.03 | ** |
|  | BPC:SDO |  | 94 | -0.77 | U= 298.0  ***z***= -7.518 | 0.000 | ** |  | ** |
| Tibia | Dda:Ddb | 71 CH  69 OA | 116.8 | -0.53 | U= 938.5  ***z***= -6.298 | 0.000 | ** | 0.05 | ** |
| Metacarpus | 1:a | 58 CH  62 OA | 91.5 | -0.82 | U= 69.0  ***z***= -9.081 | 0.000 | ** | 0.01 | ** |
|  | 1:2 |  | 67.3 | -0.80 | U= 115.0  ***z***= -8.839 | 0.000 | ** |  | ** |
|  | 4:b |  | 88.3 | -0.85 | U= 159.5  ***z***= -8.605 | 0.000 | ** |  | ** |
|  | 4:5 |  | 65 | -0.82 | U= 73.5  ***z***= -9.057 | 0.000 | ** |  | ** |
|  | SD:GL | 58 CH  61OA | 12.5 | -0.72 | U= 236.5  ***z***= -8.150 | 0.000 | ** |  | ** |
|  | BFd:GL |  | 22 | -0.84 | U= 129.5  ***z***= -8.718 | 0.000 | ** |  | ** |
| Metatarsus | 1:a | 62 CH  64 OA | 91.6 | -0.50 | U= 834.5  ***z***= -5.610 | 0.000 | ** | 0.01 | ** |
|  | 1:2 |  | 63.9 | -0.58 | U= 649.0  ***z***= -6.515 | 0.000 | ** |  | ** |
|  | 4:b |  | 92.9 | -0.25 | U= 1402.0  ***z***= -2.840 | 0.005 | * |  | ** |
|  | 4:5 |  | 62.9 | -0.48 | U= 876.5  ***z***= -5.406 | 0.000 | ** |  | ** |
|  | SD:GL | 62 CH  63 OA | 9.9 | -0.66 | U= 450.0  ***z***= -7.426 | 0.000 | ** |  | ** |
|  | BFd:GL |  | 18.8 | -0.69 | U= 378.0  ***z***= -7.779 | 0.000 | ** |  | ** |
| Astragalus | H:Dl | 72 CH  73 OA | 150.3 | -0.69 | U= 516.5  ***z***= -8.350 | 0.000 | ** | 0.01 | ** |
|  | Bd:GLl |  | 64.2 | -0.63 | U= 708.0  ***z***= -7.594 | 0.000 | ** |  | ** |
|  | Bd:H |  | 79.2 | -0.59 | U= 827.5  ***z***= -7.120 | 0.000 | ** |  | ** |
|  | Bd:Dl |  | 119.4 | -0.01 | U= 2582.0  ***z***= -0.170 | 0.865 | n.s. |  | n.s. |
|  | Dl:GL |  | 53.9 | -0.73 | U= 399.0  ***z***= -8.816 | 0.000 | ** |  | ** |
| Calcaneus | c:d | 60 CH  62 OA | 55.3 | -0.81 | U= 104.5  ***z***= -8.991 | 0.000 | ** | 0.02 | ** |
|  | c:B |  | 199 | -0.72 | U= 290.5  ***z***= -8.038 | 0.000 | ** |  | ** |
|  | DS:c |  | 149.5 | -0.62 | U= 518.0  ***z***= -6.873 | 0.000 | ** |  | ** |
| 3rd Phalanx | MBS:DLS | 72 CH  81 OA | 484.5 | -0.70 | U= 532.0  ***z***= -8.714 | 0.000 | ** | 0.05 | ** |
