# Supplementary material for: A morphometric system to distinguish sheep and goat postcranial bones
Source: PLoS One. 2017 Jun 8;12(6):e0178543. doi: 10.1371/journal.pone.0178543 (PMC5464554; doi:10.1371/journal.pone.0178543)
Supplement: S4 Table — (DOCX) [file pone.0178543.s004.docx]

S4 Table. Results from Manova for each combination of ratios used in the allometric shape analysis. *p* value significant at *p*<0.001=***.

| **Skeletal Part** | **Ratios** | **F** | **Wilk’s lambda** | ***p*** | **Significance** |
| --- | --- | --- | --- | --- | --- |
| Horncore | A/E:F | 23.41 | 0.5617 | 0.001  (3.059E-08) | *** |
|  | E:F/A:F | 60.44 | 0.3317 | 0.001  (4201E-15) | *** |
| Scapula | ASG:BG/ASG:LG | 24.78 | 0.744 | 0.001  (5.639E-10) | *** |
|  | GLP:LG/GLP:BG | 54.02 | 0.5713 | 0.001  (3.135E-18) | *** |
|  | ASG:SLC/GLP:BG | 47.92 | 0.6004 | 0.001  (1.116E-16) | *** |
| Humerus | BT:HT/BT:HTC | 37.58 | 0.6571 | 0.001  (7.383E-14) | *** |
|  | BE:Bd/BE:BT | 8.991 | 0.889 | 0.001  (0.0002091) | *** |
|  | BE:HTC/BE:BT | 36.13 | 0.6659 | 0.001  (1.928E-13) | *** |
|  | BEI:BT/BEI:Bd | 55.44 | 0.5649 | 0.001  (1.395E-18) | *** |
| Radius | BFp:Bp/Dp | 111.3 | 0.3895 | 0.001  (8.366E-30) | *** |
| Ulna | BPC:DPA/BPC:SDO | 102.4 | 0.3515 | 0.001  (6.266E-26) | *** |
| Tibia | Bd/Dda:Ddb | 31.33 | 0.6861 | 0.001  (6.226E-12) | *** |
| Metacarpal | 1:a/1:2 | 206.3 | 0.2209 | 0.001  (4.319E-39) | *** |
|  | 4:b/4:5 | 171.8 | 0.254 | 0.001  (1.524E-35) | *** |
|  | BFd:GL/SD:GL | 110.8 | 0.3436 | 0.001  (1.229E-27) | *** |
| Metatarsal | 1:a/1:2 | 31.38 | 0.6621 | 0.001  (9.733E-12) | *** |
|  | 4:b/4:5 | 18.15 | 0.7721 | 0.001  (1.237E-07) | *** |
|  | BFd:GL/SD:GL | 58.51 | 0.5104 | 0.001  (1.523E-18) | *** |
| Astragalus | H:Dl/Bd:GLl | 79.79 | 0.4709 | 0.001  (5.969E-24) | *** |
|  | H:Dl/Bd:H | 65.11 | 0.5216 | 0.001  (8.58E-21) | *** |
|  | Bd:Dl/Dl:GLl | 90.36 | 0.44 | 0.001  (4.848E-26) | *** |
|  | Bd:H/Bd:GLl | 42.48 | 0.6257 | 0.001  (3.472E-15) | *** |
| Calcaneum | c:B/c:d | 128.7 | 0.3162 | 0.001  (1.757E-30) | *** |
|  | DS:c/c:d | 152.1 | 0.2813 | 0.001  (1.666E-33) | *** |
|  | DS:c/c:B | 103.4 | 0.3653 | 0.001  (9.519E-27) | *** |
| 3^rd^ Phalanx | DLS/DLS:MBS | 95.53 | 0.4086 | 0.001  (2.215E-26) | *** |
