# Supplementary material for: A morphometric system to distinguish sheep and goat postcranial bones
Source: PLoS One. 2017 Jun 8;12(6):e0178543. doi: 10.1371/journal.pone.0178543 (PMC5464554; doi:10.1371/journal.pone.0178543)
Supplement: S5 Table — (DOCX) [file pone.0178543.s005.docx]

S5 Table. Percentage of correct classifications by element and species from Linear Discriminant Analysis.

| **Anatomical Element** | **% CH correctly identified** | **% OA correctly identified** | **Overall % of correct identifications** | **Overall % of correct identifications with cross-validation (leave one out)** |
| --- | --- | --- | --- | --- |
| Horncore | 94.3% | 96.4% | **95.2%** | 95.2% |
| Scapula | 86.5% | 86.3% | **86.4%** | 83% |
| Humerus | 89.5% | 87.1% | **88.4%** | 86.3% |
| Radius | 85.1% | 90.1% | **93.5%** | 93.5% |
| Ulna | 94.6% | 91.2% | **92.9%** | 92.0% |
| Tibia | 96.6% | 82.7% | **89.1%** | 86.4% |
| Metacarpal | 96.6% | 100% | **98.3%** | 97.5% |
| Metatarsal | 91.8% | 93.7% | **92.7%** | 91.1% |
| Astragalus | 90.3% | 87.7% | **89.0%** | 86.9% |
| Calcaneum | 91.7% | 98.4% | **95.1%** | 95.1% |
| 3^rd^ Phalanx | 83.1% | 89.9% | **85.8%** | 85.8% |
