# Supplementary material for: A morphometric system to distinguish sheep and goat postcranial bones
Source: PLoS One. 2017 Jun 8;12(6):e0178543. doi: 10.1371/journal.pone.0178543 (PMC5464554; doi:10.1371/journal.pone.0178543)
Supplement: S6 Table — (DOCX) [file pone.0178543.s006.docx]

S6 Table. Structure Matrix Table with the Correlation Coefficients for each element and each variable/measurement for Discriminant Analysis (^a=^ the variable was not used in the analysis because it correlated too highly with other variables causing multicollinearity).

|  | **Structure Matrix** | | | |
| --- | --- | --- | --- | --- |
|  | Function | | | |
|  | 1 | | | |
| **Horncore** | |  | **Metacarpal** | |
| E | .840 |  | BFd | -.528 |
| D | -.604 |  | a | -.509 |
| C | -.510 |  | GL | .488 |
| A | -.422 |  | 5 | -.481 |
| B | -.407 |  | b | -.454 |
| F^a^ | .220 |  | 2 | -.372 |
| **Scapula** | |  | SD | -.369 |
| GLP | .953 |  | 1 | .307 |
| ASG | -.589 |  | BatF | -.301 |
| LG | .298 |  | 6 | -.271 |
| BG | -.271 |  | 3 | -.253 |
| SLC | .097 |  | 4 | .212 |
| **Humerus** | |  | **Metatarsal** | |
| BEI | .627 |  | 5 | .690 |
| BE | -.409 |  | 6 | .678 |
| HTC | .406 |  | GL | -.622 |
| HT | .362 |  | 3 | .521 |
| BT | -.316 |  | b | .484 |
| Bd | -.285 |  | BFd | .466 |
| Dd | -.103 |  | 2 | .434 |
| **Radius** | |  | BatF | .406 |
| Bp | -.520 |  | SD | .369 |
| GL | .222 |  | a | .339 |
| Dp | -.178 |  | 4 | .292 |
| SD | .087 |  | 1 | -.028 |
| BFp | -.067 |  | **Tibia** | |
| **Ulna** | |  | Dda | -.682 |
| DPA | .883 |  | GL | .393 |
| BPC | -.739 |  | SD | .322 |
| SDO | .432 |  | Ddb | -.286 |
| L | .187 |  | Bd | -.250 |
| B | -.137 |  |  |  |
| **Astragalus** | |  | **Calcaneum** | |
| Dl | -.281 |  | c | .450 |
| H | .276 |  | GL | -.214 |
| Bd | -.244 |  | DS | .092 |
| GLl | .204 |  | B | -.031 |
| Dm | -.167 |  | Gd | -.030 |
| BpT | .047 |  | d | .028 |
| GLm | .031 |  | BS | -.012 |
