# Supplementary material for: A morphometric system to distinguish sheep and goat postcranial bones
Source: PLoS One. 2017 Jun 8;12(6):e0178543. doi: 10.1371/journal.pone.0178543 (PMC5464554; doi:10.1371/journal.pone.0178543)
Supplement: S1 File — (DOCX) [file pone.0178543.s007.docx]

Both inter and intra observer error tests were undertaken with the use of SPSS Statistics. For the inter-observer error, the ICC type applied (2,1) included a ‘Two-Way Random’ model, which was chosen because it is the model used when many raters, which are considered representative of a larger population, score each case only once (Landers 2011). ‘Absolute agreement’ was adopted as specificity.

For the intra-observer error, the ICC type (1,1) adopted included a ‘One Way Random’ model, which is the option to select when you have the same rater, considered as representative of a larger population, measuring each case in several occasions (Landers 2011).

As with other kinds of reliability coefficients, for ICC there is not a standard cut-off for establishing the acceptance of the level of reliability: it ranges usually from 0 to 1 where values closer to 1 are the most reliable.

Table A shows on the left side the results of the inter-observer error test for each measurement while on the right side there are the results for the intra-observer error test.

**S1A Table. Results of the inter and intra-observer error tests. ICC values for each measurement taken on different anatomical elements are given, along with 95% Confidence Interval values.**

| **Inter-Observer Error Test** | | | | **Intra-Observer Error Test** | | | |
| --- | --- | --- | --- | --- | --- | --- | --- |
| **Horncore** | | | | | | | |
| **ICC value** | | 95% Confidence Interval | | **ICC value** | | 95% Confidence Interval | |
|  |  | Lower Bound | Upper Bound |  |  | Lower Bound | Upper Bound |
| **Measurement A** | | | | | | | |
| Single Measures | .996 | .983 | 1.000 | Single Measures | 1.000 | .998 | 1.000 |
| **Measurement B** | | | | | | | |
| Single Measures | .995 | .982 | 1.000 | Single Measures | 1.000 | .999 | 1.000 |
| **Measurement C** | | | | | | | |
| Single Measures | .993 | .973 | .999 | Single Measures | 1.000 | .999 | 1.000 |
| **Measurement D** | | | | | | | |
| Single Measures | .959 | .860 | .997 | Single Measures | 1.000 | .999 | 1.000 |
| **Measurement E** | | | | | | | |
| Single Measures | .964 | .890 | .988 | Single Measures | 1.000 | 1.000 | 1.000 |
| **Measurement F** | | | | | | | |
| Single Measures | .949 | .864 | .982 | Single Measures | 1.000 | 1.000 | 1.000 |
| **Scapula** | | | | | | | |
| **Measurement BG** | | | | | | | |
| Single Measures | .982 | .936 | .999 | Single Measures | .995 | .978 | 1.000 |
| **Measurement GLP** | | | | | | | |
| Single Measures | .757 | .435 | .979 | Single Measures | .998 | .990 | 1.000 |
| **Measurement LG** | | | | | | | |
| Single Measures | .982 | .936 | .999 | Single Measures | .998 | .993 | 1.000 |
| **Measurement SLC** | | | | | | | |
| Single Measures | .962 | .868 | .997 | Single Measures | .975 | .893 | .998 |
| **Measurement ASG** | | | | | | | |
| Single Measures | .592 | .244 | .956 | Single Measures | .992 | .967 | .999 |
| **Humerus** | | | | | | | |
| **Measurement BT** | | | | | | | |
| Single Measures | .963 | .872 | .997 | Single Measures | .995 | .978 | 1.000 |
| **Measurement Bd** | | | | | | | |
| Single Measures | .935 | .793 | .995 | Single Measures | .999 | .994 | 1.000 |
| **Measurement Dd** | | | | | | | |
| Single Measures | .871 | .638 | .990 | Single Measures | .999 | .997 | 1.000 |
| **Measurement BE** | | | | | | | |
| Single Measures | .827 | .537 | .986 | Single Measures | .985 | .934 | .999 |
| **Measurement BEI** | | | | | | | |
| Single Measures | .586 | .231 | .954 | Single Measures | .975 | .895 | .998 |
| **Measurement HTC** | | | | | | | |
| Single Measures | .975 | .912 | .998 | Single Measures | .989 | .951 | .999 |
| **Measurement HT** | | | | | | | |
| Single Measures | .731 | .400 | .975 | Single Measures | .990 | .957 | .999 |
| **Radius** | | | | | | | |
| **Measurement Bp** | | | | | | | |
| Single Measures | .956 | .853 | .997 | Single Measures | .961 | .840 | .997 |
| **Measurement BFp** | | | | | | | |
| Single Measures | .905 | .717 | .993 | Single Measures | .996 | .982 | 1.000 |
| **Measurement Dp** | | | | | | | |
| Single Measures | .897 | .695 | .992 | Single Measures | .968 | .869 | .998 |
| **Measurement GL** | | | | | | | |
| Single Measures | .997 | .994 | .999 | Single Measures | .999 | .995 | 1.000 |
| **Measurement SD** | | | | | | | |
| Single Measures | .780 | .437 | .981 | Single Measures | .997 | .986 | 1.000 |
| **Ulna** | | | | | | | |
| **Measurement B** | | | | | | | |
| Single Measures | .684 | .290 | .989 | Single Measures | .989 | .942 | 1.000 |
| **Measurement L** | | | | | | | |
| Single Measures | .891 | .572 | .997 | Single Measures | .995 | .974 | 1.000 |
| **Measurement SDO** | | | | | | | |
| Single Measures | .942 | .783 | .998 | Single Measures | .985 | .935 | .999 |
| **Measurement BPC** | | | | | | | |
| Single Measures | .888 | .615 | .997 | Single Measures | .993 | .970 | 1.000 |
| **Measurement DPA** | | | | | | | |
| Single Measures | .990 | .956 | 1.000 | Single Measures | .993 | .971 | 1.000 |
| **Tibia** | | | | | | | |
| **Measurement Bd** | | | | | | | |
| Single Measures | .810 | .522 | .984 | Single Measures | .998 | .991 | 1.000 |
| **Measurement Dda** | | | | | | | |
| Single Measures | .919 | .746 | .994 | Single Measures | .995 | .977 | 1.000 |
| **Measurement Ddb** | | | | | | | |
| Single Measures | .825 | .544 | .985 | Single Measures | .991 | .959 | .999 |
| **Metacarpal** | | | | | | | |
| **Measurement GL** | | | | | | | |
| Single Measures | 1.000 | .999 | 1.000 | Single Measures | 1.000 | .999 | 1.000 |
| **Measurement SD** | | | | | | | |
| Single Measures | .876 | .636 | .990 | Single Measures | .995 | .978 | 1.000 |
| **Measurement b** | | | | | | | |
| Single Measures | .968 | .893 | .998 | Single Measures | .997 | .985 | 1.000 |
| **Measurement 1** | | | | | | | |
| Single Measures | .749 | .422 | .977 | Single Measures | .982 | .921 | .999 |
| **Measurement 2** | | | | | | | |
| Single Measures | .979 | .923 | .998 | Single Measures | .995 | .980 | 1.000 |
| **Measurement 3** | | | | | | | |
| Single Measures | .955 | .845 | .997 | Single Measures | .991 | .959 | .999 |
| **Measurement 4** | | | | | | | |
| Single Measures | .648 | .269 | .965 | Single Measures | .968 | .867 | .998 |
| **Measurement 5** | | | | | | | |
| Single Measures | .863 | .621 | .989 | Single Measures | .997 | .986 | 1.000 |
| **Measurement 6** | | | | | | | |
| Single Measures | .975 | .911 | .998 | Single Measures | .991 | .960 | .999 |
| **Metatarsal** | | | | | | | |
| **Measurement GL** | | | | | | | |
| Single Measures | .930 | .779 | .995 | Single Measures | .999 | .996 | 1.000 |
| **Measurement SD** | | | | | | | |
| Single Measures | .975 | .911 | .998 | Single Measures | .993 | .969 | 1.000 |
| **Measurement BatF** | | | | | | | |
| Single Measures | .995 | .983 | 1.000 | Single Measures | .999 | .994 | 1.000 |
| **Measurement BFd** | | | | | | | |
| Single Measures | .969 | .891 | .998 | Single Measures | .987 | .944 | .999 |
| **Measurement a** | | | | | | | |
| Single Measures | .939 | .804 | .995 | Single Measures | .993 | .970 | 1000 |
| **Measurement b** | | | | | | | |
| Single Measures | .975 | .909 | .998 | Single Measures | .991 | .959 | .999 |
| **Measurement 1** | | | | | | | |
| Single Measures | .780 | .447 | .981 | Single Measures | .985 | .935 | .999 |
| **Measurement 2** | | | | | | | |
| Single Measures | .980 | .930 | .999 | Single Measures | .995 | .979 | 1.000 |
| **Measurement 3** | | | | | | | |
| Single Measures | .957 | .856 | .997 | Single Measures | .998 | .991 | 1.000 |
| **Measurement 4** | | | | | | | |
| Single Measures | .697 | .342 | .972 | Single Measures | .946 | .789 | .996 |
| **Measurement 5** | | | | | | | |
| Single Measures | .959 | .862 | .997 | Single Measures | .996 | .981 | 1.000 |
| **Measurement 6** | | | | | | | |
| Single Measures | .896 | .689 | .992 | Single Measures | .997 | .985 | 1.000 |
| **Astragalus** | | | | | | | |
| **Measurement Bd** | | | | | | | |
| Single Measures | .991 | .968 | .999 | Single Measures | .988 | .949 | .999 |
| **Measurement GLl** | | | | | | | |
| Single Measures | .984 | .942 | .999 | Single Measures | .999 | .997 | 1.000 |
| **Measurement Dl** | | | | | | | |
| Single Measures | .577 | .230 | .954 | Single Measures | .993 | .968 | .999 |
| **Measurement GLm** | | | | | | | |
| Single Measures | .991 | .967 | .999 | Single Measures | .999 | .996 | 1.000 |
| **Measurement Dm** | | | | | | | |
| Single Measures | .336 | .057 | .896 | Single Measures | .992 | .963 | .999 |
| **Measurement H** | | | | | | | |
| Single Measures | .966 | .885 | .998 | Single Measures | .995 | .979 | 1.000 |
| **Measurement BpT** | | | | | | | |
| Single Measures | .860 | .617 | .989 | Single Measures | .992 | .964 | .999 |
| **Calcaneum** | | | | | | | |
| **Measurement GL** | | | | | | | |
| Single Measures | .462 | .189 | .687 | Single Measures | .999 | .997 | 1.000 |
| **Measurement SB** | | | | | | | |
| Single Measures | .995 | .983 | 1.000 | Single Measures | .994 | .973 | 1.000 |
| **Measurement c** | | | | | | | |
| Single Measures | .112 | -.010 | .720 | Single Measures | .984 | .930 | .999 |
| **Measurement d** | | | | | | | |
| Single Measures | .652 | .297 | .965 | Single Measures | .990 | .955 | .999 |
| **Measurement B** | | | | | | | |
| Single Measures | .757 | .418 | .978 | Single Measures | .971 | .880 | .998 |
| **Measurement DS** | | | | | | | |
| Single Measures | .923 | .756 | .994 | Single Measures | .997 | .985 | 1.000 |
| **Measurement Gd** | | | | | | | |
| Single Measures | .799 | .459 | .983 | Single Measures | .971 | .878 | .998 |
| **3^rd^ Phalanx** | | | | | | | |
| **Measurement DLS** | | | | | | | |
| Single Measures | .997 | .991 | 1.000 | Single Measures | 1.000 | .998 | 1.000 |
| **Measurement MBS** | | | | | | | |
| Single Measures | .771 | .445 | .980 | Single Measures | .989 | .953 | .999 |

All measurements taken on the horncore have provided very high ICC scores in both tests (close to 1). It is surprising to note that, despite the fact that E and F may be difficult measurements to take (i.e. no clear and constant landmarks are present on the bone, indicating where to position the callipers), they have given good results. Clearly, despite the degree of uncertainty in the positioning of the callipers, they can be taken in a relatively consistent way.

Similar results have been obtained for the scapula. Both tests have provided ICC scores which are closer to 1 than 0. Thus, measurements on this element have been taken consistently. ASG has provided a higher ICC score with the intra-observer error than with the inter-observer error, showing that - despite being a difficult measurement to take (as the area of the bone where the callipers should be placed is hard to define) - when is the same person to take the measurement repeatedly, consistency increases.

All the ICC values of the measurements taken on the humerus are high and close to 1 in both intra and inter-observer error tests. Measurement BEI in both tests has given the lowest score. Nevertheless, the results are acceptable, even in the case of the inter-observer error test, as the value is still closer to 1 than 0, indicating a certain degree of consistency. The lower consistency of BEI may have been caused by the difficulty of positioning the callipers in the right way; in fact there are no clear landmarks to take as fixed points on the lateral epicondyle.

Positive results have been also obtained by the measurements taken on the radius: in both tests they all have very high values, supporting the idea that all these measurements were taken consistently.

A high level of consistency has also been found for all measurements taken on the ulna. Measurement B has the lowest coefficient in the inter-observer error, attesting to the fact that it was taken less consistently than the others. This can partially be seen in the intra-observer error results where B produced one of the lowest scores. Nevertheless, the value is still very close to 1, which indicates a high degree of consistency. An explanation for the lower consistency of B can be found in the fact that the measurement is taken in an area which is rounded and bumpy, especially in sheep. It is therefore difficult to position the callipers in a consistent way.

Good results have been provided also by all measurements taken on the tibia, as attested by relatively high ICC scores in both the inter-observer error and the intra-observer error.

All of the measurements related to the metacarpal have provided high ICC values demonstrating that they were taken consistently. The measurements which have given the lowest ICC values are, in both tests, 1 and 4. These are, however, still very high values showing that the measurements were taken consistently. The reason behind the slightly lower degree of consistency of 1 and 4 could be related to the fact that the description regarding the position of the callipers on the external trochlea of the medial and lateral condyles was unclear. As a consequence, some colleagues have taken it more medially (as suggested by Davis 1996 and Payne 1969), rather than on the external edge, as we originally intended.

Unsurprisingly, very similar results have been obtained for the metatarsal. Measurements 1 and 4 have the lowest coefficients, suggesting that they were taken less consistently than all the other measurements. Nevertheless, since their ICC scores are far closer to 1 than 0, they are still acceptable.

For the astragalus, when the intra-observer error results are considered, all measurements have been taken consistently. The outcome is different when the inter-observer error test is considered. In fact, the results for Dl and Dm are low compared to the other measurements, revealing that they were taken less consistently by the raters. This can be explained by the shape of the lateral and medial side of the astragalus: as they are not regular surfaces (particularly the medial side in goat) they are difficult to measure in a consistent way.

A similar situation can be attested for the calcaneum. When the intra-observer error values are considered, it can be seen that all measurements have provided very high ICC scores. The results of the inter-observer error are less satisfactory. In fact, measurement GL and c have a very low ICC. The reason behind the low performance of GL - which is a straightforward and routinely taken measurement (von den Driesch 1976) - is unusual. This result is probably due to one or more errors by the raters which is probably not significant for our understanding of the replicability of that measurement. For c on the other hand, the problem could be in the shape of the articular facet. Boessneck himself (1969:353) defines this measurement as “imprecise”.

All measurements taken on the 3^rd^ phalanx have given satisfactory ICC values in both tests. In both intra and inter-observer error tests DLS seems to have been taken more consistently than MBS.
