# Supplementary material for: A morphometric system to distinguish sheep and goat postcranial bones
Source: PLoS One. 2017 Jun 8;12(6):e0178543. doi: 10.1371/journal.pone.0178543 (PMC5464554; doi:10.1371/journal.pone.0178543)
Supplement: S2 File — (DOCX) [file pone.0178543.s008.docx]

The data used are from the sheep and goat scapulae found at the medieval site of Woolmonger/Kingswell Street in Northampton (bones from phase 2, *i.e.* c. AD 1100-1400).

An initial identification of the archaeological specimens was conducted by using the morphological approach and no goats were identified. The biometrical results, as shown in Fig 2A, agree with the morphological outcome, as concerns the specimens positively identified to species. All archaeological specimens morphologically identified as sheep are consistent with the sheep modern group pattern. Two of the unidentified specimens (*Ovis/Capra*) plot in the area of overlap but are much closer to the centre of the sheep distribution. Another unidentified specimen is border-line in the sheep distribution and much more consistent with the goat range; it may indeed represent a rare occurrence of the goat at this site.


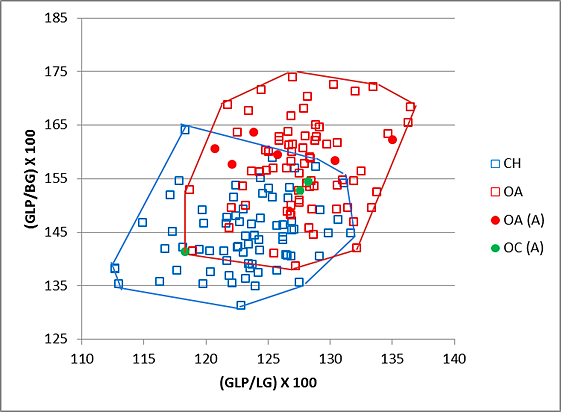


S2A Fig. Scapula. Ratio between the greatest length of the *processus articularis* and the length of the glenoid cavity plotted against the ratio between the greatest length of the *processus articularis* and the breadth of the glenoid cavity. The modern data are represented by the square empty symbol: blue for modern *Capra hircus*, red for modern *Ovis aries*. The archaeological material is represented by the full dot symbol: red for *Ovis aries* and green for *Ovis/Capra*.

Discriminant Analysis was then applied on all sheep and goat archaeological scapulae for the site. The results revealed that the degree of agreement between morphological and biometrical identifications for this element for the archaeological specimens is higher (100%) than that provided by the modern material (86.4%). No archaeological specimens have been ‘misattributed’ by the DA (i.e. not attributed to the group of their morphological identification). Of the six unidentified specimens, one has been identified as goat and five as sheep (Table A).

Fig 2B presents visually the data provided in Table A. All archaeological specimens morphologically identified as sheep gather around the group centroid of the sheep group. Most morphologically unidentified specimens also plot close to the sheep group centroid, while one coincides almost exactly with the goat centroid. This specimen is the one previously discussed as a probable goat in Fig S2A. Considering the separation between this specimen and the sheep group (Fig S2B), the DA identification is likely to be correct, confirming what was previously seen with the use of the biometrical indices: the unidentified specimen is almost certainly a goat.

**S2A Table. Results of the Discriminant Analysis when applied on all the archaeological scapulae from the site. CH= *Capra hircus*; OA= *Ovis aries*; OC= *Ovis/Capra.* Total= total number of specimens; a= percentage of correct reattributions on the modern material; b= percentage of correct reattributions on the archaeological material; d= percentage of correct reattributions on the archaeological material when cross validation is applied.**

| **Classification Results^a,b,d^** | | | | | | |
| --- | --- | --- | --- | --- | --- | --- |
|  |  |  | TAXA | Predicted Group Membership | | Total |
|  |  |  |  | CH | OA |  |
| Modern Material | Original | Count | CH | 64 | 10 | 74 |
|  |  |  | OA | 10 | 63 | 73 |
|  |  | % | CH | 86.5 | 13.5 | 100.0 |
|  |  |  | OA | 13.7 | 86.3 | 100.0 |
|  | Cross-validated^c^ | Count | CH | 61 | 13 | 74 |
|  |  |  | OA | 12 | 61 | 73 |
|  |  | % | CH | 82.4 | 17.6 | 100.0 |
|  |  |  | OA | 16.4 | 83.6 | 100.0 |
| Woolmonger/Kingswell Street Material | Original | Count | CH | 0 | 0 | 0 |
|  |  |  | OA | 0 | 16 | 16 |
|  |  |  | OC | 1 | 5 | 6 |
|  |  | % | CH | .0 | .0 | 100.0 |
|  |  |  | OA | .0 | 100.0 | 100.0 |
|  |  |  | OC | 16.7 | 83.3 | 100.0 |
| a. 86.4% of selected original grouped cases (modern material) correctly classified. | | | | | | |
| b. 100.0% of unselected original grouped cases (archaeological material) correctly classified. | | | | | | |
| d. 83.0% of selected cross-validated grouped cases correctly classified. | | | | | | |


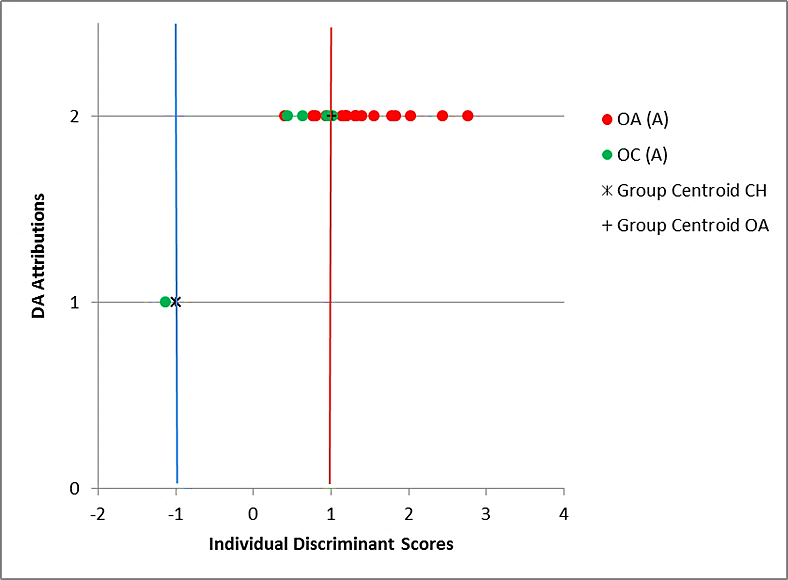


S2B Fig. Scapula. Diagram of the individual discriminant scores attributed to the archaeological scapulae from Woolmonger/Kingswell Street by DA. On the horizontal axis are the individual discriminant scores attributed to each archaeological specimen, on the vertical axis is the species attribution assigned by the program (1= *Capra hircus*; 2= *Ovis aries*). The red and blue vertical lines on the graph represent the group centroids for each species, red line for *Ovis aries* and blue line for *Capra hircus*. The full dots represent the morphological identification of the archaeological material: red dot= *Ovis aries*; green dot= *Ovis/Capra* (sheep/goat).
